# An Iridium Distannylidyne – Expanding the Scope of Tetrylidyne Transition Metal Coordination Chemistry

**DOI:** 10.1002/anie.1048718

**Published:** 2026-08-24

**Authors:** Lennart G. Holzapfel, Eric Hinssen, Stefan F. Miehe, Klaus Eichele, Hartmut Schubert, Christian P. Sindlinger, Lars Wesemann

**Affiliations:** ^1^ Institut für Anorganische Chemie Eberhard Karls Universität Tübingen Tübingen Germany; ^2^ Institut für Anorganische Chemie Universität Stuttgart Stuttgart Germany

**Keywords:** azide, carbon dioxide, iridium, main group element, tin

## Abstract

Although transition metal tetrylidynes have been known for several decades, analogous heavy ditetrylidynes have not been reported in the literature to this day. In this work we describe the synthesis and characterization of iridium distannylidyne [(Me_3_P)_2_Ir(SnAr*)_2_][WCA] (**1a**/**1b**) {[WCA] = [Al(O*^t^* Bu^F^)_4_]^–^ (**1a**), [BAr^F^_4_]^–^ (**1b**); Ar* = 2,6‐bis(2,4,6‐tri‐*iso*‐propylphenyl)phenyl, *^t^* Bu^F^ = C(CF_3_)_3_, Ar^F^ = 3,5‐bis(trifluoromethyl)phenyl} as the first transition metal ditetrylidyne. **1** reacts with two equivalents of CO_2_ to give [(Me_3_P)_2_Ir(SnAr*)_2_(*μ*_3_‐*η* ^3^‐*κ* ^3^‐*O*,*C*,*O’*‐CO_2_)_2_][Al(O*^t^* Bu^F^)_4_] (**2**). In the presence of excess trimethylphosphine, trimethylsilyl azide reacts with **1** to form imido‐bridged [(Me_3_P)_3_Ir(SnAr*)_2_(*μ*_2_‐N–TMS)][BAr^F^_4_]  (**3**) upon extrusion of N_2_.

## Graphical abstract

This work presents the synthesis, characterization, and reactivity of an iridium distannylidyne as the first heavy transition metal ditetrylidyne. Characterized by its short Ir─Sn bonds and linear bonding angles at the tin atoms, this compound displays versatile reactivity toward carbon dioxide and trimethylsilyl azide.

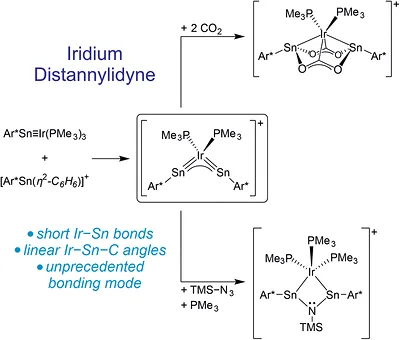

## 1 Introduction

The heavy homologues of transition metal carbynes, the transition metal tetrylidynes, have been extensively studied over the past three decades [1, 2, 3, 4, 5, 6, 7, 8, 9, 10, 11, 12], revealing their ability to activate small molecules through metal–ligand cooperativity [13, 14, 15, 16, 17, 18, 19, 20, 21, 22, 23]. These compounds are characterized by a short M–E triple bond and a near‐linear M–E–R unit (M = transition metal, E = Si–Pb, R = substituent) [13, 24, 25, 26]. Quantum chemical investigations suggest that the tetrel atom donates its lone pair to the transition metal, forming a *σ*‐bond, while the transition metal exerts two π‐bonds by backdonation from its d‐orbitals into the vacant p‐orbitals of the tetrylidyne fragment [R–E:]^+^, resulting in a triple bond between the metal and tetrel atoms [27, 28, 29, 30]. Surprisingly, there are only a few transition metal compounds with two independent [R–E:] fragments bound to the metal center. To the best of our knowledge, Power et al. reported [Fe(CO)_4_(PbAr*)_2_] as the first transition metal ditetrylene (Scheme 1A), synthesized by reacting diplumbyne Ar*PbPbAr* with Fe_2_(CO)_9_ [31]. Recently, Tilley and coworkers synthesized the iridodistannylene [Cp*Ir(H)_2_(SnAr’)_2_] [Ar’ = 2,6‐bis(2,4,6‐trimethylphenyl)phenyl] (Scheme 1A) by treating Cp*IrH_4_ with two equivalents of the amidostannylene Ar'SnN(TMS)_2_ [32]. The Wolf group reported the synthesis and characterization of [Fe(SnAr^dipp^)_2_] [Ar^dipp^ = 2,6‐bis(2,6‐di‐*iso*‐propylphenyl)phenyl] (Scheme 1B) featuring two asymmetrically bound Ar^dipp^Sn moieties at the iron atom, which is simultaneously *η* ^6^‐coordinated by an adjacent arene of one of the Ar^dipp^ ligands [33, 34]. With these compounds establishing the field of transition metal complexes featuring two tetrylidyne ligands, there have been no further reports of heavy transition metal ditetrylidynes. Building on our previous work allowing us to synthesize iridium tetrylidyne in relatively large quantities [Ar*Sn≡Ir(PMe_3_)_3_, 256 mg, 89%] [22], we sought to explore possible synthetic pathways to this group of compounds, given their potentially diverse reactivity that would result from such a bonding mode (Scheme 1C–E).

**SCHEME 1:**
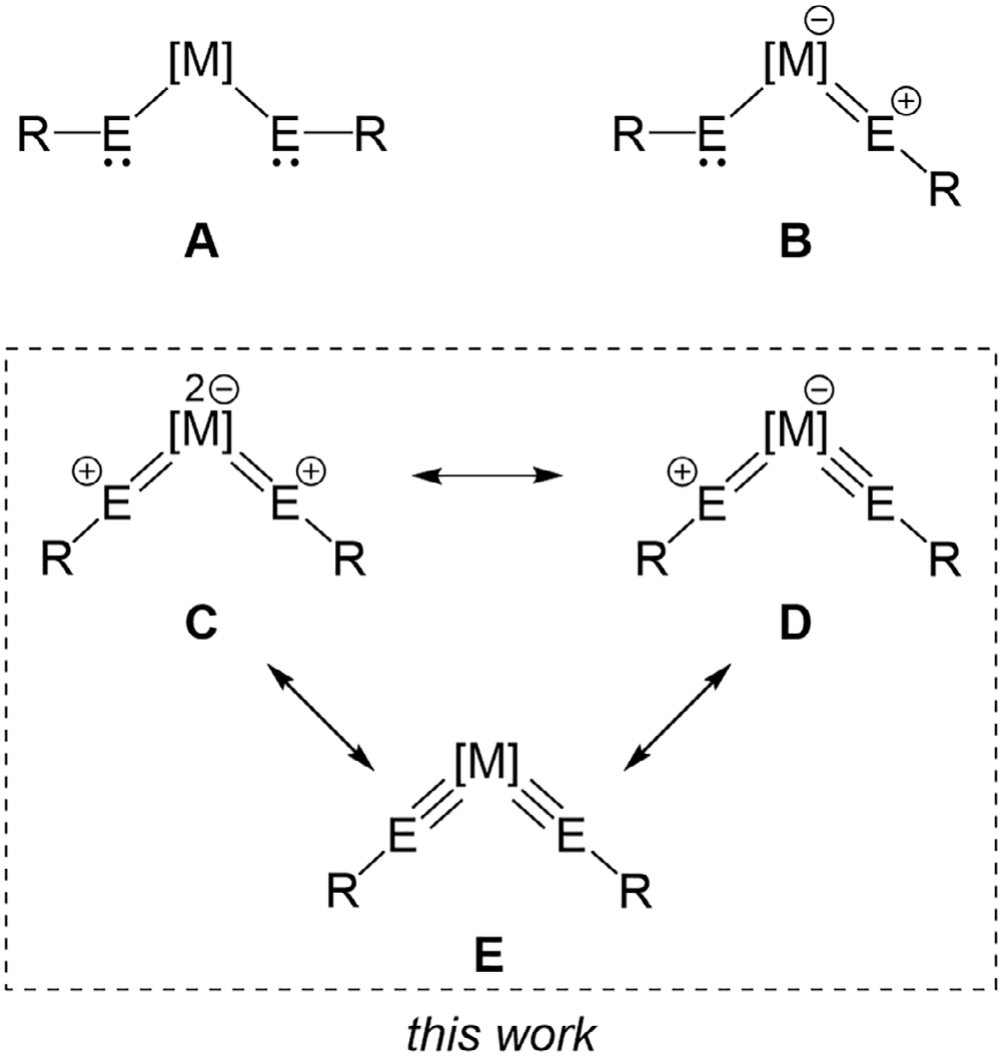
Schematic representation of different bonding modes between a transition metal and two tetrylidyne ligands ([M] = transition metal fragment with ligand sphere, E = Si–Pb, R = substituent).

## 2 Results and Discussion

The in‐situ generated stannylidinium ion [Ar*Sn(*η* ^2^‐C_6_H_6_)][WCA] [35, 36] ([WCA] = [BAr^F^_4_]^–^, [Al(O*^t^* Bu^F^)_4_]^–^) [37] instantaneously reacts with the iridium stannylidyne Ar*Sn≡Ir(PMe_3_)_3_ [22] to substitute a phosphine ligand forming [(Me_3_P)_2_Ir(SnAr*)_2_][WCA] (**1a**/**1b**) {[WCA] = [Al(O*^t^* Bu^F^)_4_]^–^ (**1a**), [BAr^F^_4_]^–^ (**1b**)} (Scheme 2). Monitoring of the reaction progress with ^31^P{^1^H} NMR spectroscopy indicates full conversion after ten minutes at rt. After workup and crystallization from *ortho*‐difluorobenzene layered with *n*‐pentane at rt, **1** is obtained as brown crystals in 71% yield. Distannylidyne **1** does not show any signs of decomposition after being heated to 80°C for several days in solution.

**SCHEME 2:**
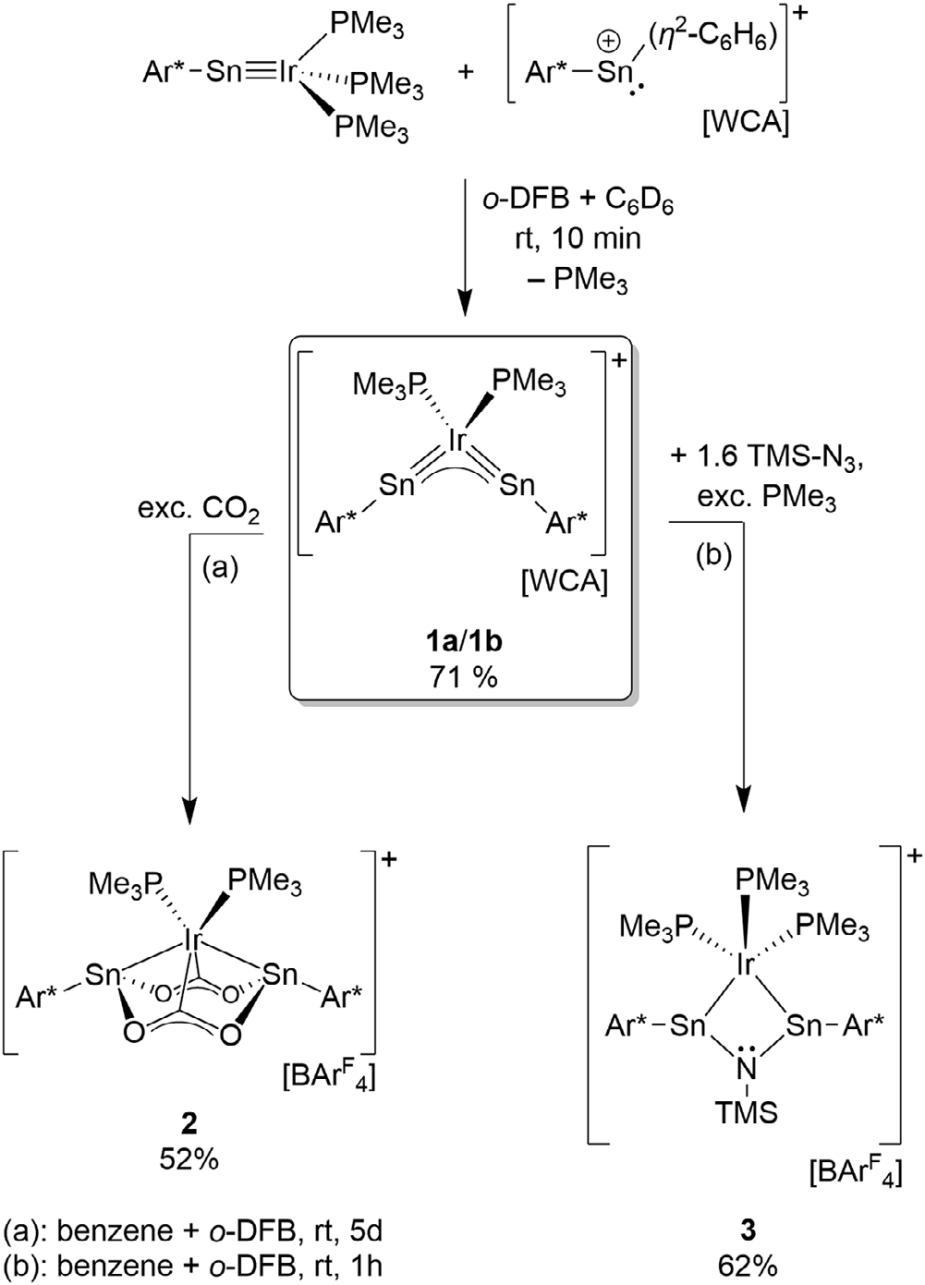
Synthesis of iridium distannylidyne **1** and its reaction with excess carbon dioxide to give the twofold addition product **2**, as well as the reaction with trimethylsilyl azide in the presence of excess trimethylphosphine to give the imido‐bridged **3** (*o*‐DFB = *ortho*‐difluorobenzene).

The ^1^H NMR spectrum of [(Me_3_P)_2_Ir(SnAr*)_2_][Al(O*^t^* Bu^F^)_4_] (**1a**) shows one set of signals for the terphenyl ligands as well as one signal for the trimethylphosphine ligands, suggesting high symmetry in the compound. Further underpinning this assumption, the ^31^P{^1^H} NMR spectrum shows one signal at −33.2 ppm exhibiting ^119/117^Sn satellites (^2^ *J*_Sn,P_ =  248.3 Hz) whose integrals relative to the main signal indicate the coupling of the phosphorus nuclei to two tin atoms [38, 39, 40]. In line with this observation, the ^119^Sn{^1^H} NMR spectrum shows a triplet at 1253 ppm (DFT: 1034 ppm averaged) [41, 42, 43] (^2^ *J*_Sn,P_ =  248 Hz) as well as a set of ^117^Sn satellites with a coupling constant of ^2^ *J*_Sn,Sn_ = 3625 Hz. The chemical shift lies slightly above previously reported transition metal stannylidynes (Rh≡Sn 1149 [18], Co≡Sn 1100 [19], Mo≡Sn 1040 [44] ppm) [45]. The Sn–Sn coupling constant of 3625 Hz lies in the range of other tetrahedral transition metal compounds: ^2^ *J*_119Sn,117Sn_ = 2500 Hz [Pt{bis(stannylene)}_2_] [46], 3200 Hz [Au{bis(stannylene)}_2_] [47], 4517 Hz [(Ph_3_P)_2_Pt{Sn(acac)_2_}_2_] [48].

The molecular structure of **1a** (Figure 1) was determined by single‐crystal x‐ray diffraction analysis and reveals two symmetrically bound SnAr* moieties at the iridium center. The Ir─Sn1/2 bond lengths [2.4031(2), 2.4074(2) Å] lie between those in the iridium stannylidyne Ar*Sn≡Ir(PMe_3_)_3_ [2.3909(3) Å] [22] and [(Me_3_P)_3_HIr═SnTbb]^+^ [2.4135(5) Å] [45]. Similarly, the Ir─Sn1/2–C*_ipso_* bond angles [175.6(1), 176.9(1)°] are comparable to the one in Ar*Sn≡Ir(PMe_3_)_3_ [177.79(10)°] [22] and close to linearity. Based on these structural features, compound **1** represents the first example of a transition‐metal complex featuring two linear MSnAr moieties. Related dicarbynes based on tungsten [49, 50, 51], tantalum [52], iron [53], and molybdenum [54] have been reported.

**FIGURE 1:**
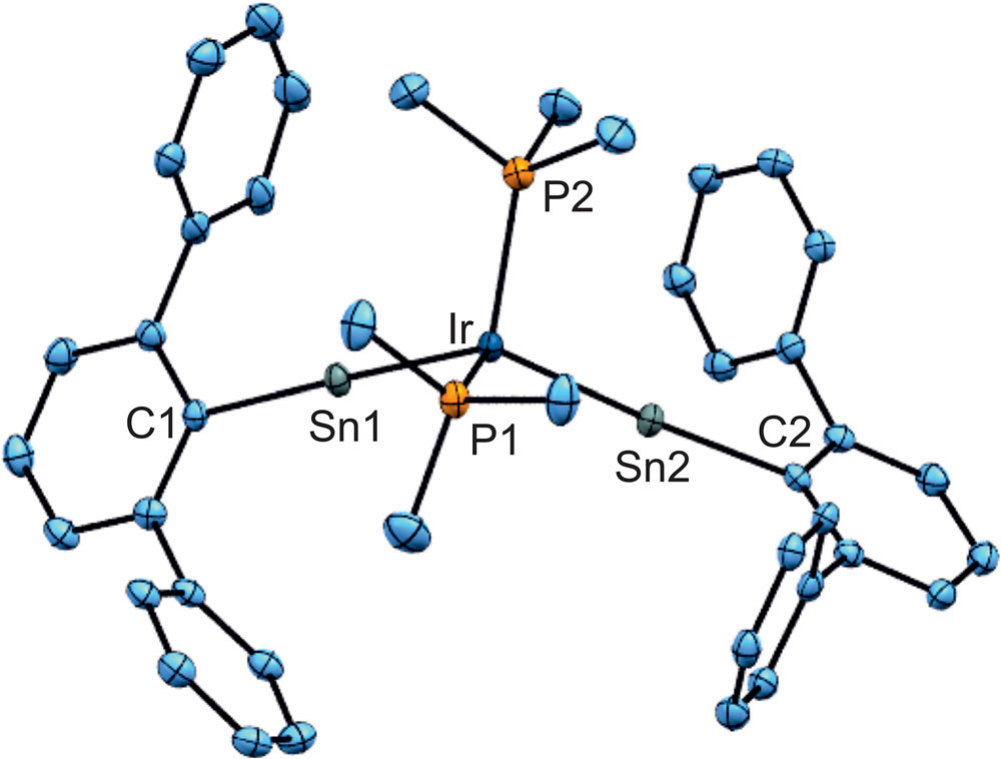
Molecular structure of cationic iridium distannylidyne [(Me_3_P)_2_Ir(SnAr*)_2_][Al(O*^t^* Bu^F^)_4_] (**1a)** with thermal ellipsoids set at 50% probability. Hydrogen atoms, *^i^* Pr substituents, and [Al(O*^t^* Bu^F^)_4_]^–^ are omitted for clarity. Selected bond lengths [Å] and angles [°]: Ir‐Sn1 2.4031(2), Ir‐Sn2 2.4074(2), Ir‐Sn1‐C1 175.6(1), Ir‐Sn2‐C2 176.9(1), Sn1‐Ir‐Sn2 116.4(1).

To gain further insights into the electronic structure of **1**, DFT [BP86 D3BJ/ def2SVP; Ir, Sn, P def2TZVP] [55, 56, 57, 58, 59, 60, 61, 62, 63, 64] studies on **1** were performed. The HOMO of **1**, as well as the HOMO–1, the HOMO–2, and the HOMO–16, each correspond to a three‐center‐two‐electron (3c2e) π‐bonding interaction within the Sn–Ir–Sn moiety (Figure 2) [53]. In conjunction with the two Ir─Sn σ‐bonds found in lower‐lying MOs (Figures S32–S34), a bond order of three can be tentatively assigned to both Ir─Sn bonds. The results of the intrinsic bond orbital analysis (IBO), two Ir–Sn π‐bonds and an Ir─Sn *σ*‐bond for each Ir–Sn unit, corroborate this interpretation (Figure 3) [65]. The NLMO analysis reveals five filled d‐orbitals at the iridium, giving the iridium atom a formal charge of Ir(–1), leading to a formal separation of **1** into a [Ir(PMe_3_)_2_]^–^ and two [Ar*Sn]^+^ fragments.

**FIGURE 2:**
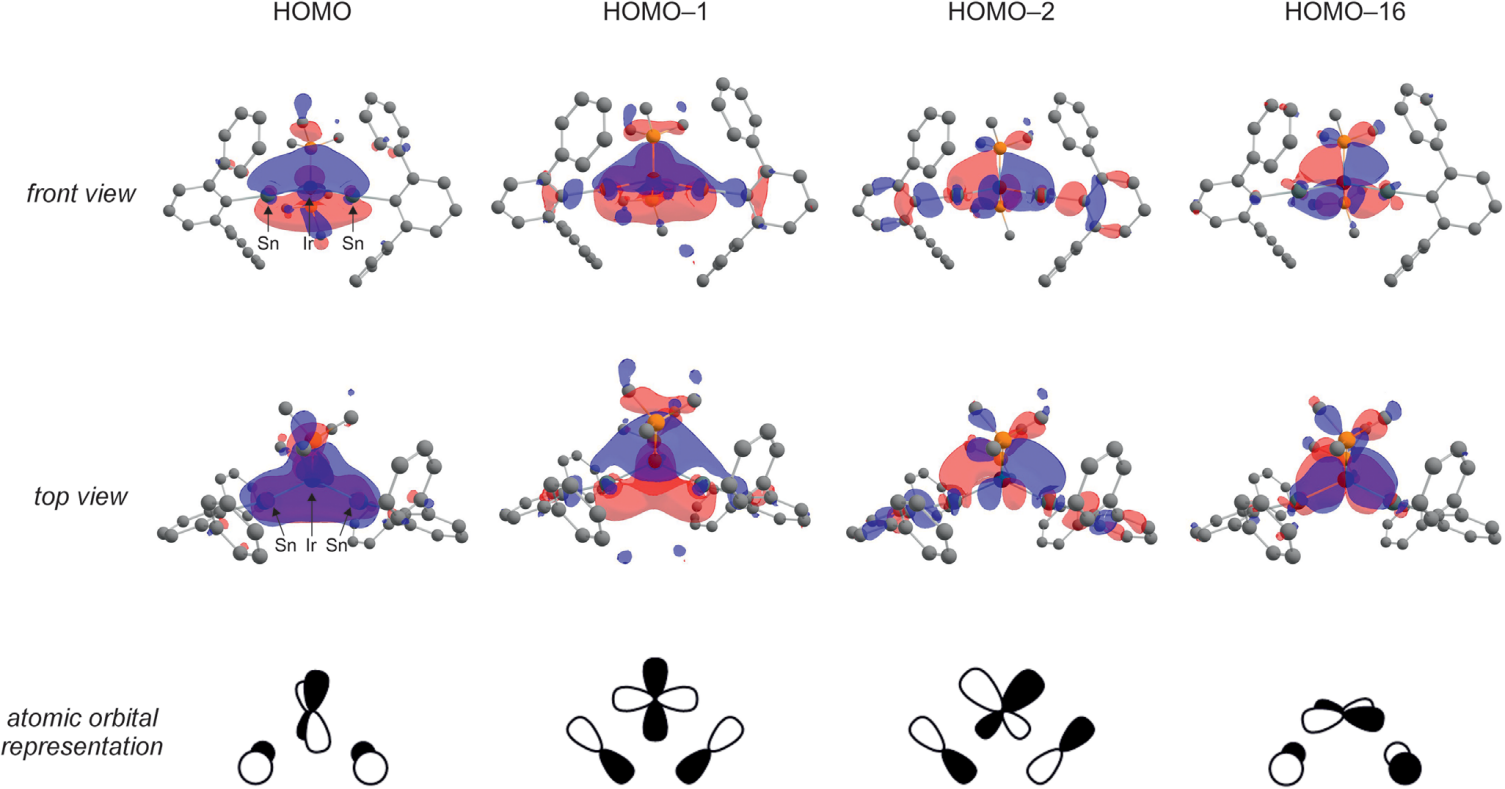
Selected molecular orbitals of **1** as well as the corresponding atomic orbital representation of the respective 3c2e bond (contour value = 0.03 a.u.) [53, 66].

**FIGURE 3:**
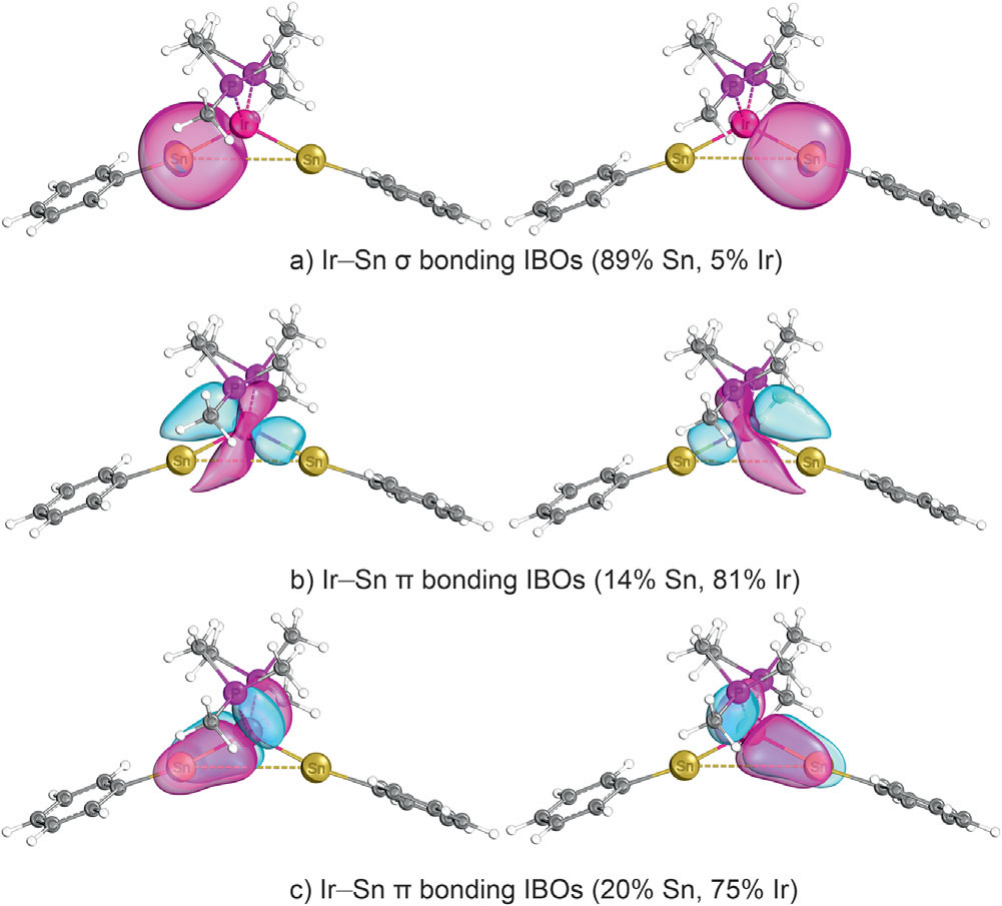
Selected IBOs of **1** and the atomic distributions.

However, the calculated Mayer bond orders (MBO) (Ir–Sn1/2: 1.48/1.50) are smaller than the corresponding value of 1.64 in Ar*Sn≡Ir(PMe_3_)_3_ [22] and, instead, comparable to the calculated MBO (1.50) for a [Fe = SnAr^dipp^] moiety reported by Wolf and coworkers indicating an average bond order of less than three in the Sn–Ir–Sn moiety [33]. To further analyze the bonding in **1**, an extended transition state‐natural orbitals for chemical valence (ETS‐NOCV) analysis on a model complex was performed using ADF (see Supporting Information for details) [67]. Individual bonding interactions between the [MeSn]^+^ and [Ir(PMe_3_)_2_]^–^ fragments were evaluated in ETS‐NOCV analysis resulting from the stepwise removal of a [MeSn]^+^ fragment from [(MeSn)_2_Ir(PMe_3_)_2_]^+^ and from [(MeSn)Ir(PMe_3_)_2_]. NOCV for [(MeSn)_2_Ir(PMe_3_)_2_]^+^: two similarly strong (−47.0 and −41.4 kcal mol^−1^) π‐type interactions from donation of the [(MeSn)Ir(PMe_3_)_2_] fragment into the perpendicular empty p‐orbitals of the stannylidinium fragment, along with a weaker (−20.0 kcal mol^−1^), yet clear *σ*‐type interaction arising from donation of the Sn‐lone pair to the [(MeSn)Ir(PMe_3_)_2_] fragment were found. We would argue that these three interactions allow for a description of a triple bond. However, the NOCV of the [(MeSn)Ir(PMe_3_)_2_] fragment indicates that here the [MeSn]^+^ fragment shows only two bonding interactions with the iridium in [Ir(PMe_3_)_2_]^−^—a π‐type and a *σ*‐type bond. Accordingly, the averaged bond order in the Sn–Ir–Sn moiety would be around 2.5. Thus, the mesomeric formulas C and D (Scheme 1) should be considered as significant contributions to the bonding mode in **1**.

To explore the reactivity of distannylidyne **1**, a sample was treated with excess carbon dioxide in solution at rt. After five days, the reaction was complete, giving compound **2** in a 52% yield after workup and crystallization (Scheme 2, left). The molecular structure of **2** (Figure 4) reveals a twofold addition of carbon dioxide onto the Sn–Ir–Sn moiety in a bridging *μ*_3_‐*η* ^3^‐*κ* ^3^‐*O*,*C*,*O’* manner [68, 69, 70, 71, 72, 73, 74, 75]. The C─O bond lengths in **2** [1.279(6)–1.304(6) Å] lie between single and double bond lengths between these elements and are comparable to the ones in the rhodium‐osmium compound [{(COD)Rh}_2_OsH_2_CO_2_(PMe_2_Ph)_3_] [1.300(21), 1.309(22) Å] [68, 69] and other similar complexes [70, 71, 72, 73, 74, 75]. The interatomic distances Ir–Sn1/2 [2.5605(3), 2.5460(3) Å] are in the range of single bonds between these elements [32, 45]. The C3⋯C4 distance (2.424 Å) is significantly longer than the longest comparable bonds in metal‐coordinated oxalate moieties [1.79(5) Å] [76].

**FIGURE 4:**
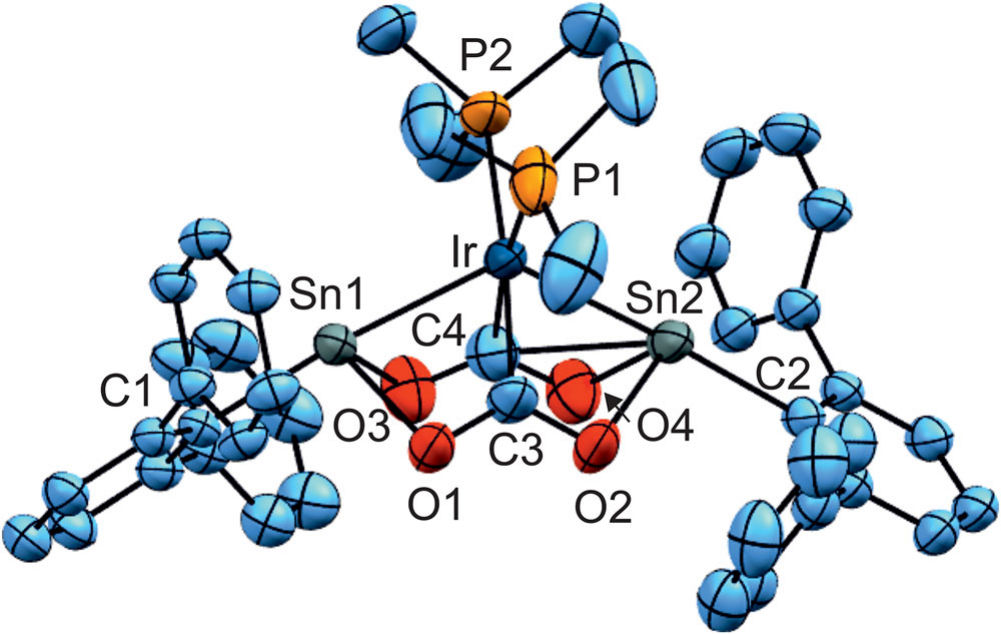
Molecular structure of [(Me_3_P)_2_Ir(SnAr*)_2_(*μ*_3_‐*η* ^3^‐*κ* ^3^‐*O*,*C*,*O’*‐CO_2_)_2_][Al(O*^t^* Bu^F^)_4_] (**2**) with thermal ellipsoids set at 50% probability. Hydrogen atoms, *^i^* Pr substituents, and [Al(O*^t^* Bu^F^)_4_]^–^ are omitted for clarity. Selected bond lengths [Å] and angles [°]: Ir‐Sn1 2.5605(3), Ir‐Sn2 2.5460(3), Ir‐C3 2.050(4), Ir‐C4 2.000(5), C3⋯C4 2.424, O1‐C3 1.285(5), O2‐C3 1.292(5), O3‐C4 1.279(6), O4‐C4 1.304(6), Ir‐Sn1‐C1 174.8(1), Ir‐Sn2‐C2 176.8(1), Sn1‐Ir‐Sn2 125.5(1), O1‐C3‐O2 120.1(4), O3‐C4‐O4 120.0(5), O1‐Sn1‐O3 87.6(2), O2‐Sn2‐O4 87.1(2).

Surprisingly, the Ir–Sn–C*_ipso_* angles [174.8(1), 176.8(1)°] are only negligibly smaller than the ones in distannylidyne **1** [175.6(1), 176.9(1)°] and remain nearly linear. To explain this geometry, we suggest that the tin centers retain their sp hybridization during the reaction while the π‐backdonation from the iridium is no longer present [45]. Instead, the resulting vacant p‐orbitals at the tin atoms are stabilized by donation from two adjacent oxygen atoms (Figure 5a). To substantiate this model, DFT [BP86 D3BJ/def2SVP, Ir,Sn,P def2TZVP] calculations and NBO analyses were performed [77]. The NLMOs reveal three lone pairs at each oxygen atom which all show similar interactions with the adjacent atoms: One is purely located at the oxygen atom (Figures 5c), one engages in π‐bonding (Figures 5d) toward the neighboring carbon atom (atomic contribution on C atom 12.6%–21.3%) while the last shows partial donation (atomic contribution on Sn atom 5.3%–5.7%) toward the respective tin atom (Figures 5b). These findings explain the observed C─O bond lengths (*vide supra*) and support our bonding model for **2**. The HOMO and LUMO of **2** are shown in the Supporting Information (Figure S35).

**FIGURE 5:**
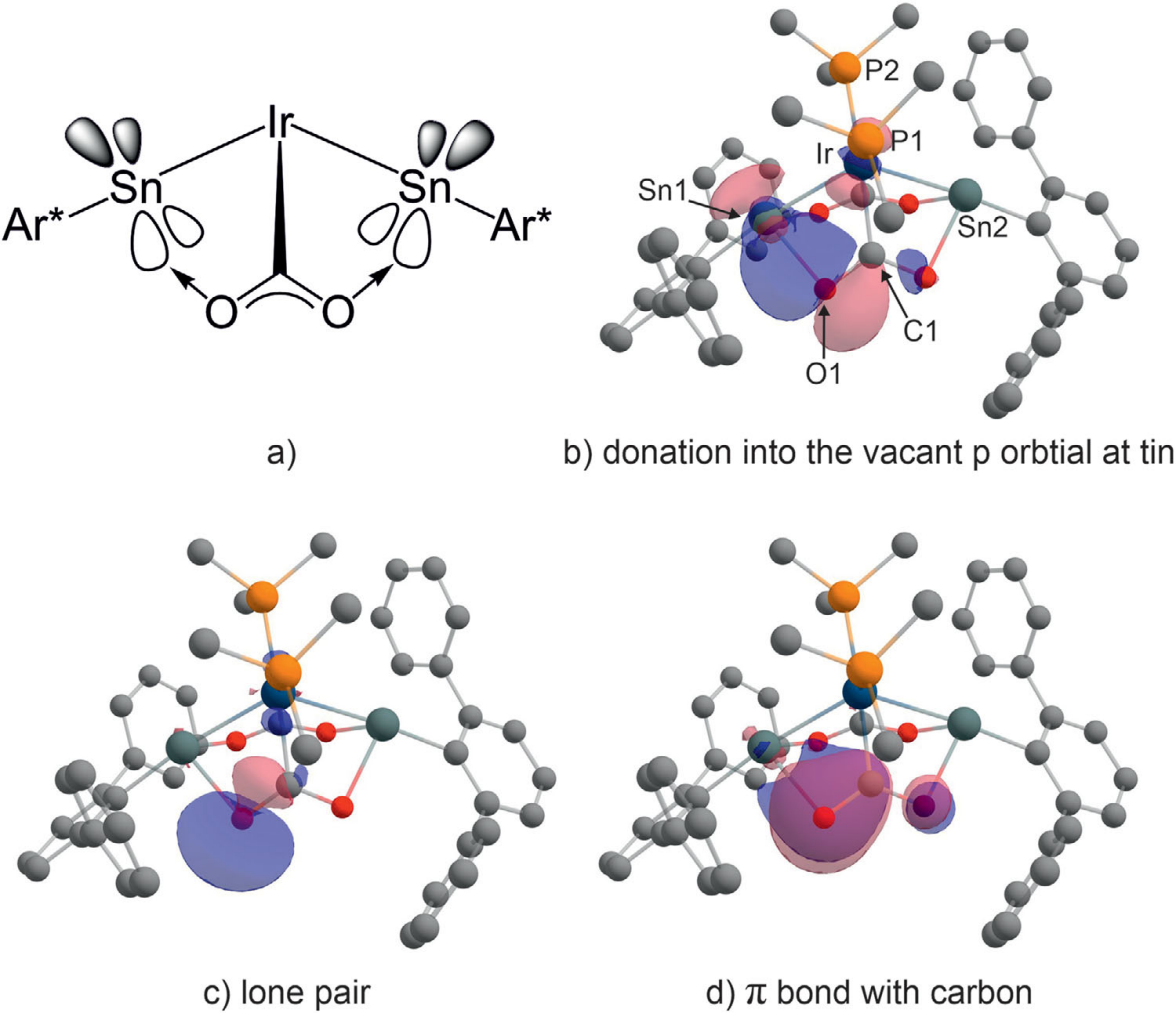
(a) Schematic representation of the bonding of one CO_2_ moiety in **2**, and (b–d) NLMOs showing one set of oxygen lone pair interactions (contour value = 0.03 a.u.).

The ^31^P{^1^H} NMR spectrum of **2** features one signal at −54.9 ppm exhibiting ^119/117^Sn satellites (^2^ *J*_Sn,P_ =  67.1 Hz) corresponding to two adjacent tin atoms (*vide supra*). The ^13^C{^1^H} NMR spectrum features a remarkable, second‐order signal from 199.2–200.0 ppm that is assigned to the CO_2_ carbon atoms. The signal corresponds to an AA'X spin system, suggesting two magnetically inequivalent phosphine ligands. The most astonishing NMR spectroscopic feature is the signal at −692 ppm in the ^119^Sn{^1^H} NMR spectrum, exhibiting ^117^Sn satellites with a coupling constant of 14466 Hz. The strong shift toward lower frequencies compared to the distannylidyne **1** (1253 ppm) may be rationalized by the donation of oxygen lone pairs into the formerly empty p‐orbitals at tin, removing low‐lying vacant orbitals from the paramagnetic contribution to the magnetic shielding. This is corroborated by the DFT calculations of magnetic shielding tensors (see SI) that show, in the case of **1**, that the two directions of least shielding are perpendicular to the Sn─Ir bond, where σ→π* mixing is active, whereas in **2** those directions are more shielded by about 2500 ppm. The ^119^Sn NMR chemical shift was confirmed by GIAO NMR calculations (*δ* = −521 ppm, averaged calc. chem. shift) [ADF, GGA/revPBE, TZP SO/ZORA] [41, 42, 43]. Similar shifts have been reported for four‐coordinate tin species [78, 79]. As previous studies have shown a positive dependence of ^2^ *J* coupling constants on the bonding angle of the coupling nuclei, the large ^2^ *J*_Sn,Sn_ coupling constant (14466 Hz) is attributed to the increased Sn─Ir─Sn bonding angle in **2** [125.5(1)°] compared to **1** [116.4(1)°] [40, 80, 81]. Transition metal compounds with two *trans* tin ligands have been shown to have ^2^ *J*_Sn,Sn_ coupling constants of 37164 Hz in *trans*‐[Pt(SnCl_3_)_3_(AsEt_3_)_2_] [40] and 40759 Hz in *cis*‐[IrCl_2_(SnCl_3_)_4_]^3–^ [80].

Distannylidyne **1** selectively reacts with an excess of trimethylsilyl azide in the presence of excess trimethylphosphine to give the imido‐bridged [(Me_3_P)_3_Ir(SnAr*)_2_(*μ*_2_‐N–TMS)][BAr^F^_4_] (**3**) as an olive‐green powder in 62% yield after workup and crystallization (Scheme 2, right). The molecular structure (Figure 6) reveals an almost symmetrical, planar four‐membered ring corroborated by the sum of angles at Sn1, Sn2, and N (360.0°, 359.6°, and 359.7°). One signal in the ^31^P{^1^H} NMR spectrum at –52.2 ppm featuring ^119/117^Sn satellites (^2^ *J*_Sn,P_ =  491.1 Hz) for three phosphine ligands indicates a dynamic process at the penta‐coordinated iridium center [45]. The signal in the ^119^Sn{^1^H} NMR spectrum was observed at 619 ppm, lying at smaller frequencies in comparison to the imido‐bridged distannylene [Ar^dipp^Sn{*μ* ^2^‐N(SiMe_3_)}SnAr^dipp^, ^119^Sn = 907 ppm] [82]. The ^119^Sn NMR chemical shift of **3** was confirmed by GIAO NMR calculations (*δ* = 729 ppm) [ADF, GGA/revPBE, TZP SO/ZORA].

**FIGURE 6:**
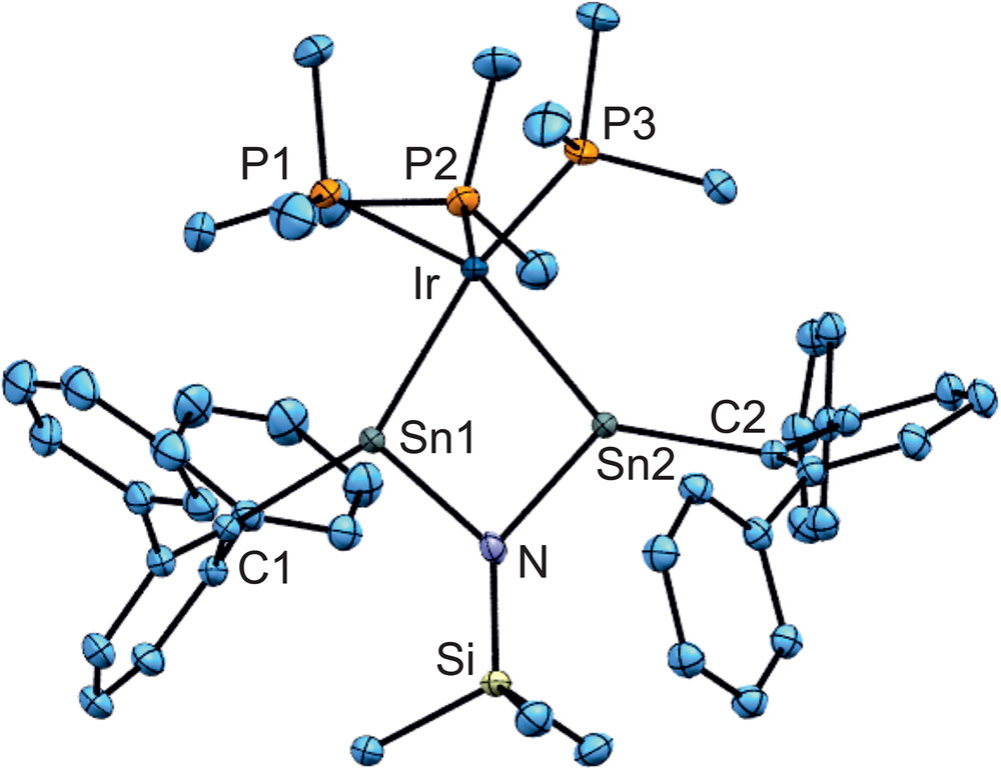
Molecular structure of [(Me_3_P)_3_Ir(SnAr*)_2_{*μ*_2_‐N(SiMe_3_)}][BAr^F^_4_]  (**3**) with thermal ellipsoids set at 50% probability. Hydrogen atoms, *^i^* Pr substituents, and [BAr^F^_4_]^–^ are omitted for clarity. Selected bond lengths [Å] and angles [°]: Ir‐Sn1 2.5415(2), Ir‐Sn2 2.6002(2), Sn1‐N 2.0875(17), Sn2‐N 2.1149(16), Ir‐Sn1‐C1 152.0(1), Ir‐Sn2‐C2 139.7(1), Ir‐Sn1‐N 100.0(1), Ir‐Sn2‐N 97.4(1), Sn1‐Ir‐Sn2 71.4(1), Sn1‐N‐Sn2 91.1(1).

The Sn1/Sn2–N bond lengths [2.0875(17), 2.1149(16) Å] lie in the range of tin(II) amides [Ar^dipp^Sn{*μ*_2_‐N(SiMe_3_)}SnAr^dipp^: Sn–N 2.111(6), 2.055(6) Å, and {(Me_3_Si)_2_N–Sn(*μ*‐O_2_CAr’)}_2_: N–Sn 2.090(2), 2.087(2) Å] [82, 83]. The interatomic distances Ir–Sn1/Sn2 [2.5415(2), 2.6002(2) Å] are comparable to single bonds between these elements (*vide supra*). Similar imido‐bridged compounds derived from the reaction of metal precursors with organic azides have been described in the literature [84, 85, 86, 87]. Figure 7 shows selected NLMOs of **3**. The nitrogen atom features two p‐orbitals—one lying in the plane of the four‐membered ring, one perpendicular to it. Both contain a lone pair and exhibit slight delocalization toward the adjacent tin centers, forming a polarized 3c2e‐σ and ‐π bond (a and b, respectively). One of the two remaining orbitals forms the Si–N σ‐bond (c), while the second holds a third lone pair that shows slight delocalization toward the two tin atoms as well (d). The HOMO and LUMO of **3** are displayed in the Supporting Information (Figure S36).

**FIGURE 7:**
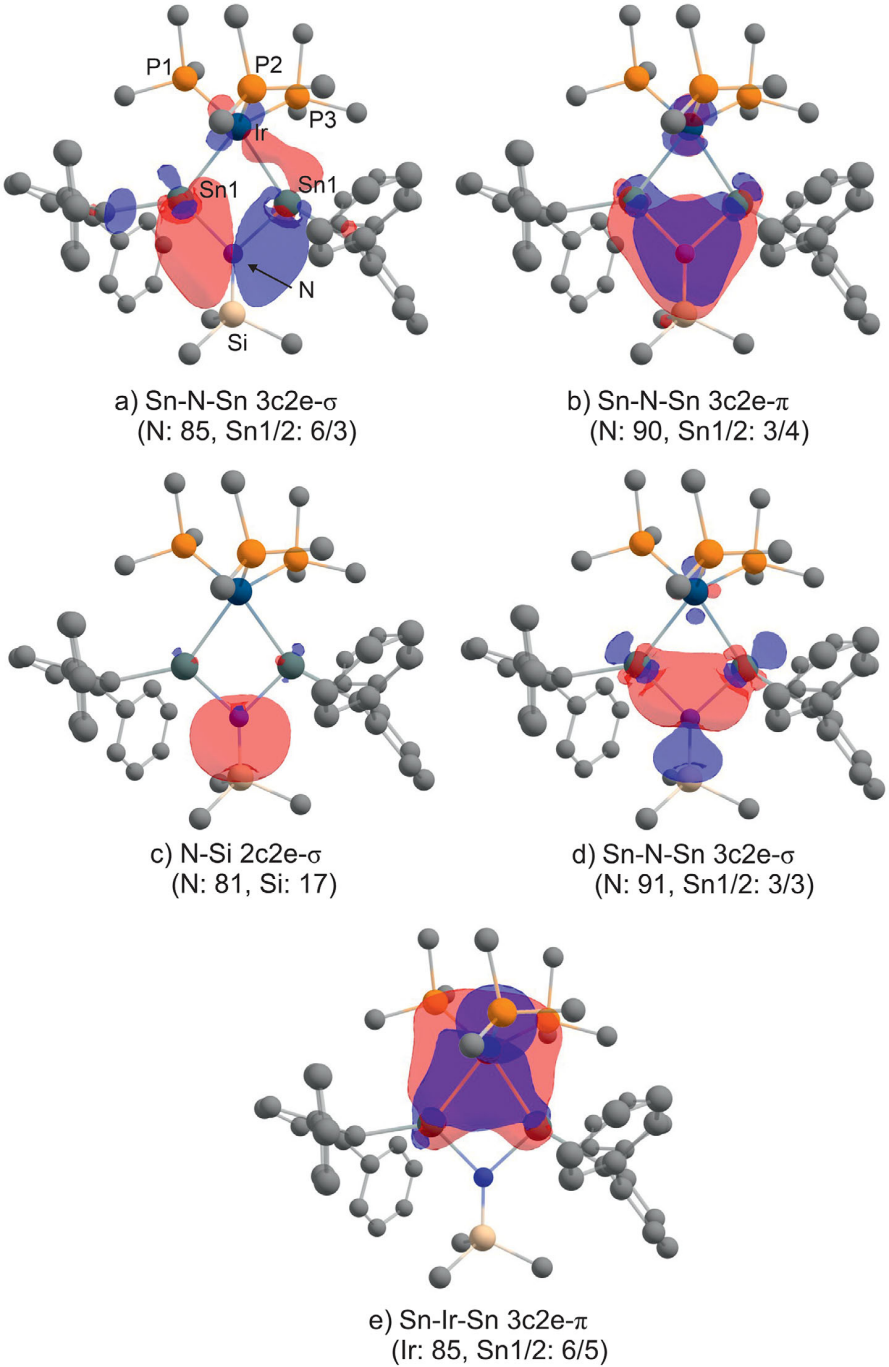
Selected NLMOs of compound **3** (contour value = 0.03 a.u.). The atomic distributions (%) are given in brackets. DFT [BP86 D3BJ/def2SVP; Ir, Sn, P def2TZVP]. For orbital contributions, see Table S2.

Mechanistic studies using the nudge elastic band (NEB) method [88, 89, 90, 91, 92, 93] suggest that **1** initially reacts with Me_3_Si–N_3_ in a (3+3)‐cycloaddition reaction upon PMe_3_ coordination, yielding a six‐membered ring. Subsequently, molecular nitrogen is extruded to form a transient stanna imine that finally attacks the second tin center to form the four‐membered ring in **3**. Further information is given in the Supporting Information.

## 3 Conclusion

Herein, we present the facile synthesis and characterization of [(Me_3_P)_2_Ir(SnAr*)_2_][WCA] (**1a**/**1b**) as the first heavy transition metal ditetrylidyne. The two Ir─Sn bonds are characterized by short bond lengths, near‐linear bonding angles at the tin atoms, and an average bond order of 2.5. The unprecedented coordination mode of the two stannylidyne ligands enables reactivity with carbon dioxide in a twofold *μ*_3_‐*η* ^3^‐*κ* ^3^‐*O*,*C*,*O’* manner onto the Sn–Ir–Sn moiety as well as the formation of an imido bridge between the two tin centers in the reaction with trimethylsilyl azide and excess trimethylphosphine. This work expands the scope of low‐valent group 14 coordination chemistry at transition metals by establishing heavy ditetrylidynes as a distinct bonding motif and highlights their potential for cooperative and unconventional small molecule activation.

## Author Contributions

**Lennart G. Holzapfel**: investigation, validation, formal analysis, writing – original draft. **Eric Hinssen**: investigation. **Stefan F. Miehe**: investigation, writing – original draft, validation. **Klaus Eichele**: formal analysis. **Hartmut Schubert**: formal analysis. **Christian P. Sindlinger**: writing – review and editing, investigation. **Lars Wesemann**: supervision, writing – review and editing.

## Conflicts of Interest

The authors declare no conflicts of interest.

## Supporting information



Additional Supporting Information can be found online in the Supporting Information section. Further supporting information, including detailed synthetic procedures, spectroscopic and crystallographic data on the compounds, as well as computational details, is provided in the Supporting Information file. CIF files of the structures reported have been deposited with the CCDC database (deposition numbers 2562465–2562467) [94]. The authors have cited additional references within the Supporting Information [95, 96, 97, 98, 99, 100, 101, 102, 103, 104, 105, 106, 107, 108, 109, 110, 111, 112, 113, 114, 115, 116, 117]. **Supporting File**: anie74273‐sup‐0001‐SuppMat.pdf.

## Data Availability

The data that support the findings of this study are available in the Supporting Information of this article.
