# Supplementary material for: An Iridium Distannylidyne – Expanding the Scope of Tetrylidyne Transition Metal Coordination Chemistry
Source: Angew Chem Int Ed Engl. 2026 Aug 24;65(41):e1048718. doi: 10.1002/anie.1048718 (PMC13638086; doi:10.1002/anie.1048718)
Supplement: Supplementary file 1 [file ANIE-65-e1048718-s001.pdf]

Supporting Information

An Iridium Distannylidyne – Expanding the Scope of Tetrylidyne Transition Metal Coordination Chemistry

Lennart G. Holzapfel, Eric Hinssen, Stefan F. Miehe, Klaus Eichele, Hartmut Schubert, Christian Sindlinger*, Lars Wesemann*

Content

Experimental 2

Syntheses 4

Data of crystal structure determinations 8

NMR-Spectroscopy 9

NMR spectra of compound **1a** 9

NMR spectra of compound **2** 16

NMR spectra of compound **3** 24

UV-Vis spectroscopy 34

IR-Spectroscopy 38

Quantum chemistry 39

ETS-NOCV analysis 45

NMR chemical shift calculations 47

Minimum energy path computations 51

References 72

Experimental

General information. All manipulations were performed under argon (99.999 %) atmosphere using standard Schlenk techniques or an *MBraun* Glovebox. Additionally, the argon for Schlenk techniques is dried through SICAPENT®. All solvents were degassed via three "freeze-pump-thaw" cycles and dried with diverse methods. *n*-Hexane and *n*-pentane were obtained from an *MBraun* solvent purification system (SPS). Diethyl ether, tetrahydrofuran and toluene were obtained from an *MBraun* solvent purification system (SPS) and analogous to benzene-d_6 and toluene-d_8 distilled from a Na/K alloy. Benzene, benzene-d_6 and *o*-difluorobenzene were dried over activated aluminium oxide. $[Ar^*SnH]_2$ (Ar* = $(2,6\text{-Trip})_2C_6H_3$, Trip = 2,4,6- $iPr_3C_6H_2$),[1] and $[Ar^*Sn{\equiv}Ir(PMe_3)_3]$[2] were synthesized following the respective literature. $[C_7H_7][Al(O^tBu^F{}_4)_4]$ was synthesized following a literature procedure starting with $[Ph_3C][Al(O^tBu^F{}_4)_4]$.[3, 4] Carbon dioxide and trimethylsilylazide were commercially purchased (*Sigma Aldrich*, *ABCR, Fisher Scientific, TCI Chemicals, Westfahlen AG*) and were used as received. All air and moisture sensitive chemicals were stored under argon atmosphere.

NMR spectroscopy. The NMR spectra were recorded with one of the following spectrometers with the respective equipment:

- *Bruker* Avance III HD 300 NanoBay, 5 mm BBFO probe head operating at 121.49 (^{31}P), 111.92 (^{119}Sn) MHz.
- *Bruker* AVII+400, 5 mm QNP (quad nucleus probe) head (for ^{1}H, ^{13}C, ^{31}P spectra) or a 5 mm H-1,X+F-19 BBFO ATM head (Quad Systems), operating at 400.11 (^{1}H), 128.37 (^{11}B), 100.62 (^{13}C), 104.26 (^{27}Al), 161.97 (^{31}P), 149.20 (^{119}Sn), 376.43 (^{19}F) MHz.
- *Bruker* Avance III HDX 600, variable temperature set up, 5 mm Prodigy BBO Cryo probe head, operating at 600.13 (^{1}H), 150.92 (^{13}C), 242.94 (^{31}P), 223.79 (^{119}Sn), 564.63 (^{19}F) MHz.

The chemical shifts are reported as δ in ppm relative to the following external standards: tetramethylsilane (^{1}H, ^{13}C), 85% phosphoric acid (^{31}P) and tetramethyl tin (^{119}Sn).[5] The chemical shifts were referenced using the chemical shift of the solvents ^{2}H resonance frequency as followed: Ξ = 32.083974 % for ^{11}B, Ξ = 25.145020 % for ^{13}C, Ξ = 94.094011 % for ^{19}F, Ξ = 26.056 859 % for ^{27}Al, Ξ = 40.480742 for ^{31}P, and Ξ = 37.290632 % for ^{119}Sn.[5] The multiplicity of the signals is abbreviated as s = singlet, d = doublet, t = triplet, q = quartet, sept = septet and m = multiplet or br. = broad/unresolved. The proton and carbon signals were assigned via detailed analysis of ^{1}H, ^{13}C{^{1}H}-(UDEFT), ^{13}C{^{1}H}-DEPT135, ^{1}H-^{1}H-COSY, ^{1}H-^{13}C-HSQC and ^{1}H-^{13}C-HMBC NMR spectra. Selected 1D-NMR spectra of the compounds can be found in the Supporting Information. Simulated spectra were produced using the Daisy module of Bruker TopSpin.

IR spectroscopy. The IR spectra were recorded with a Bruker VERTEX 70 IR spectrometer using an ATR unit or in KBr.

Crystal structure determination. X-ray data were collected with a *Bruker* Smart APEX II diffractometer with graphite monochromated Mo-K_α radiation. The used programs were Bruker APEX2 v2011.8-0 including SADABS for absorption correction, SAINT for data reduction, SHELXS for structure solution and SHELXLE or WinGX suite of programs v1.70.01 including SHELXL[6] for structure refinement.[7-14] All details of the structure refinement and solution can be found in the Supporting Information.

Data were collected on a XtaLAB Synergy, Dualflex, HyPix diffractometer using ω scans with Cu K_α radiation. The crystal was kept at a steady temperature during data collection. The diffraction pattern was indexed, and the total number of runs and images was based on the strategy calculation from the program CrysAlisPro 1.171.44.115a (Rigaku Oxford Diffraction, 2025), which was also used for refining the unit cell and data reduction. Structure solution was performed with SHELXT[15] and refined[16] using SHELXL[6] along the graphical user interphase of SHELXLE[10] (Olex2 1.5-ac5-024) as the graphical interface.

UV/Vis Spectroscopy. Visible UV/Vis absorption spectra were recorded on PerkinElmer Lambda 35 spectrophotometer in gas tight 1 cm quartz cuvettes sealed by Teflon stoppers or Teflon lined screw caps.

Elemental Analysis. The elemental analysis was performed at the Department for Inorganic
Chemistry at the University of Tübingen using a vario MICRO CUBE or UNICUBE by *elementar*.

Syntheses

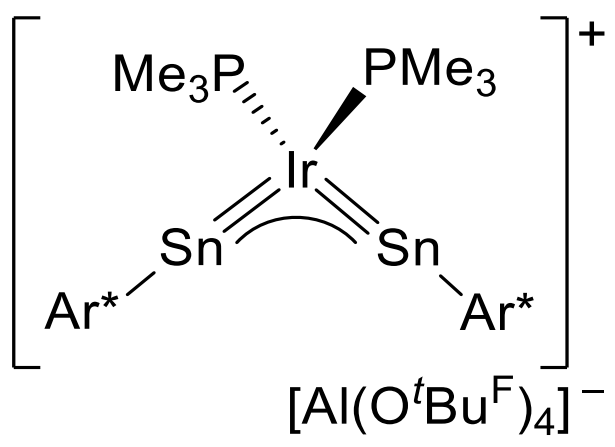

Compound **1a**: $[Ar*SnH]_2$ (38.7 mg, 32.2 µmol, 0.50 equiv.) and $[C_7H_7][Al(O^tBu^F{}_4)_4]$ (68.1 mg, 64.4 µmol, 1.00 equiv.) are weighed in together, suspended in benzene (0.1 ml) and *o*-difluorobenzene (0.5 ml) and stirred until all solids are dissolved and the mixture has turned orange. The solution of the *in situ* generated $[Ar*Sn(\eta^2\text{-}C_6H_6)][Al(O^tBu^F{}_4)_4]$ is added dropwise to a stirred solution of $Ar*Sn{\equiv}Ir(PMe_3)_3$ (65.7 mg, 64.4 µmol, 1.00 equiv.) in *o*-difluorobenzene (0.4 ml) at room temperature. After ten minutes, all volatiles are removed under reduced pressure, the brown residue is co-evaporated with *n*-pentane (2x1.0 ml) and afterwards washed with *n*-pentane (3x1.0 ml). The crude product is crystallized from a concentrated *o*-difluorobenzene solution layered with *n*-pentane at room temperature to yield $[(PMe_3)_2Ir(SnAr*)_2][Al(O^tBu^F{}_4)_4]$ (114 mg, 45.6 µmol, 71%) as dark brown crystals suitable for x-ray diffraction. *Analytical data*: **^{1}H-NMR** [600.13 MHz, 298.0 K, benzene-d_6 + *o*-difluorobenzene]: δ (ppm) 1.07 (d, 24H, $^3J_{H\text{-}H}$ = 6.8 Hz, *o*-^{i}Pr-C$\underline{H}_3$), 1.18–1.22 (m, 42H, 24x*o*-^{i}Pr-C$\underline{H}_3$ + 18xP(C$\underline{H}_3$)$_3$), 1.31 (d, 24H, $^3J_{H\text{-}H}$ = 6.9 Hz, *p*-^{i}Pr-C$\underline{H}_3$), 2.76 (sept, 8H, $^3J_{H\text{-}H}$ = 6.9 Hz, *o*-^{i}Pr-C$\underline{H}$), 2.88 (sept, 4H, $^3J_{H\text{-}H}$ = 6.9 Hz, *p*-^{i}Pr-C$\underline{H}$), 7.09–7.11 (m, 4H, *m*-$C_6\underline{H}_3$), 7.11 (m, 8H, *m*-$\underline{H}_{trip}$), 7.19–7.23 (m, 2H, *p*-$C_6\underline{H}_3$). **^{13}C{^{1}H}-NMR** [150.92 MHz, 298.0 K, benzene-d_6 + *o*-difluorobenzene]: δ (ppm) 23.8 (s, *p*-^{i}Pr-$\underline{C}H_3$), 24.3 (s, *o*-^{i}Pr-$\underline{C}H_3$), 24.6 (s, *o*-^{i}Pr-$\underline{C}H_3$), 30.7 (s, *o*-^{i}Pr-$\underline{C}H$), 34.3 (s, *p*-^{i}Pr-$\underline{C}H$), 34.9–35.2 (m, P($\underline{C}H_3$)$_3$), 79.7 (br. m, Al{O$\underline{C}$(CF$_3$)$_3$}$_4$), 121.9 (s, *m*-$\underline{C}_{trip}$), 121.9 (q, $^1J_{F\text{-}C}$ = 292.9 Hz, Al{OC($\underline{C}F_3$)$_3$}$_4$), 130.4 (s, *p*-$\underline{C}_6H_3$), 131.6 (s, *m*-$\underline{C}_6H_3$), 133.8 (s, *i*-$\underline{C}_{trip}$), 142.6 (s, *o*-$\underline{C}_6H_3$), 147.3 (s, *o*-$\underline{C}_{trip}$), 150.3 (s, *p*-$\underline{C}_{trip}$), 191.2 (s, *i*-$\underline{C}_6H_3$). **^{19}F-NMR** [564.63 MHz, 298.0 K, benzene-d_6 + *o*-difluorobenzene]: δ (ppm) −74.9 (s, C$\underline{F}_3$). **^{27}Al{^{1}H}-NMR** [156.38 MHz, 298.0 K, benzene-d_6 + *o*-difluorobenzene]: δ (ppm) 35.3 (s, $\underline{Al}$(OC(CF$_3$)$_3$}$_4$). **^{31}P{^{1}H}-NMR** [121.49 MHz, 298.0 K, benzene-d_6 + *o*-difluorobenzene]: δ (ppm) −33.2 (s + sat. (Sn$_2$), 2P, $^2J_{119/117Sn\text{-}P}$ = 248.3 Hz, $\underline{P}$(CH$_3$)$_3$). **^{119}Sn{^{1}H}-NMR** [224.04 MHz, 298.0 K, benzene-d_6 + *o*-difluorobenzene]: δ (ppm) 1253.2 (br. t + sat., 2Sn, $^2J_{119Sn\text{-}117Sn}$ = ca. 3625 Hz, $^2J_{Sn\text{-}P}$ = ca. 250 Hz). **UV/Vis** (*o*-difluorobenzene, c = 0.07 mmol·L^{-1}): λ (nm) (ε (L·mol^{-1}·cm^{-1})): 481 (4490). **Elemental analysis** calculated for $C_{94}H_{116}AlF_{36}IrO_4P_2Sn_2$ (%): C 44.94, H 4.65. Found: C 44.93, H 4.42.

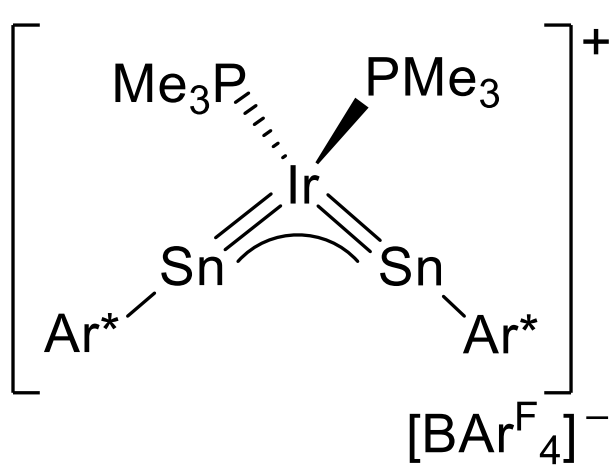

Compound **1b**: $[Ar^*SnH]_2$ (15.9 mg, 13.2 μmol, 0.50 equiv.) and $[C_7H_7][BAr^F_4]$ (25.2 mg, 26.4 μmol, 1.00 equiv.) are weighed in together, suspended in benzene (0.1 ml) and *o*-difluorobenzene (0.1 ml) and stirred until all solids are dissolved and the mixture has turned orange. The solution of the *in situ* generated $[Ar^*Sn(\eta^2\text{-}C_6H_6)][BAr^F_4]$ is added dropwise to a solution of $Ar^*Sn{\equiv}Ir(PMe_3)_3$ (27.0 mg, 26.4 μmol, 1.00 equiv.) in *o*-difluorobenzene (0.4 ml) at room temperature whereafter the reaction mixture is stirred under stationary reduced pressure. After one hour, all volatiles are removed under reduced pressure, the brown residue is co-evaporated with *n*-pentane (2x1.0 ml) and afterwards washed with *n*-pentane (3x1.0 ml). The crude product is crystallized from a concentrated *o*-difluorobenzene solution layered with *n*-hexane at –35 °C to yield $[(PMe_3)_2Ir(SnAr^*)_2][BAr^F_4]$ (33.7 mg, 14.0 μmol, 53%) as dark brown crystals. *Analytical data*: **^{1}H-NMR** [400.11 MHz, 299.2 K, benzene-d_6 + *o*-difluorobenzene]: δ (ppm) 1.06 (d, 24H, $^3J_{H\text{-}H}$ = 6.8 Hz, *o*-^{i}Pr-C$\underline{H}_3$), 1.15 (d, 18H, $^2J_{P\text{-}H}$ = 10.5 Hz, P(C$\underline{H}_3$)$_3$), 1.19 (d, 24H, $^3J_{H\text{-}H}$ = 7.0 Hz, *o*-^{i}Pr-C$\underline{H}_3$), 1.29 (d, 24H, $^3J_{H\text{-}H}$ = 7.0 Hz, *p*-^{i}Pr-C$\underline{H}_3$), 2.76 (sept, 8H, $^3J_{H\text{-}H}$ = 6.8 Hz, *o*-^{i}Pr-C$\underline{H}$), 2.87 (sept, 4H, $^3J_{H\text{-}H}$ = 6.9 Hz, *p*-^{i}Pr-C$\underline{H}$), 7.08–7.13 (m, 12H, 8x*m*-$\underline{H}_{trip}$ + 4x*m*-$C_6\underline{H}_3$), 7.19–7.23 (m, 2H, *p*-$C_6\underline{H}_3$), 7.63 (br. s, 4H, *p*-BAr^F_4-$\underline{H}$), 8.31–8.36 (br. m, 8H, *o*-BAr^F_4-$\underline{H}$). **^{11}B-NMR** [128.37 MHz, 299.2 K, benzene-d_6 + *o*-difluorobenzene]: δ (ppm) −6.0 (s, $\underline{B}Ar^F_4$). **^{13}C{^{1}H}-NMR** [100.62 MHz, 299.2 K, benzene-d_6 + *o*-difluorobenzene]: δ (ppm) 23.8 (s, *p*-^{i}Pr-$\underline{C}H_3$), 24.3 (s, *o*-^{i}Pr-$\underline{C}H_3$), 24.6 (s, *o*-^{i}Pr-$\underline{C}H_3$), 30.7 (s, *o*-^{i}Pr-$\underline{C}$H), 34.3 (s, *p*-^{i}Pr-$\underline{C}$H), 34.9–35.2 (m, P($\underline{C}H_3$)$_3$), 117.5 (br. s, *p*-BAr^F_4-$\underline{C}$), 121.8 (s, *m*-$\underline{C}_{trip}$), 124.8 (q, $^1J_{F\text{-}C}$ = 272.4 Hz, $\underline{C}F_3$), 129.5 (q, $^2J_{F\text{-}C}$ = 32.2 Hz, *m*-BAr^F_4-$\underline{C}$), 130.4 (s, *p*-$\underline{C}_6H_3$), 131.6 (s, *m*-$\underline{C}_6H_3$), 133.8 (s, *i*-$\underline{C}_{trip}$), 135.0 (s, *o*-BAr^F_4-$\underline{C}$), 142.6 (s, *o*-$\underline{C}_6H_3$), 147.3 (s, *o*-$\underline{C}_{trip}$), 150.3 (s, *p*-$\underline{C}_{trip}$), 162.4 (q, $^1J_{C\text{-}B}$ = 49.8 Hz, *i*-BAr^F_4-$\underline{C}$), 191.1 (*i*-$\underline{C}_6H_3$–Sn). **^{19}F{^{1}H}-NMR** [376.43 MHz, 299.2 K, benzene-d_6 + *o*-difluorobenzene]: δ (ppm) −62.4 (s, C$\underline{F}_3$). **^{31}P{^{1}H}-NMR** (161.97 MHz, 299.2 K, benzene-d_6 + *o*-difluorbenzene): δ (ppm) = −33.2 (s + sat. (Sn_2), 2P, $^2J_{119/117Sn\text{-}P}$ = 237.9 Hz, $\underline{P}(CH_3)_3$). **^{119}Sn{^{1}H}-NMR** [224.07 MHz, 298.0 K, benzene-d_6 + *o*-difluorobenzene]: δ (ppm) 1255.1 (br. s, FWHH = 736 Hz. **UV/Vis** (*o*-difluorobenzene, c = 0.077 mmol·L^{-1}): λ (nm) (ε (L·mol^{-1}·cm^{-1})): 598 (2567), 465 (4169).

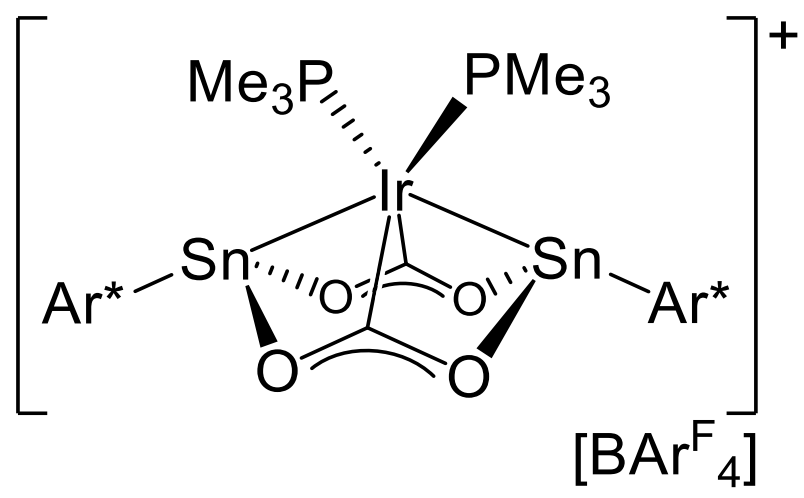

Compound **2:** $[(PMe_3)_2Ir(SnAr^*)_2][BAr^F_4]$ (65.1 mg, 27.0 µmol, 1.00 equiv.) is weighed into a *J. Young* NMR tube and soluded in benzene-d_6 (0.4 ml) and *o*-difluorobenzene (0.1 ml). The brown solution is degassed with one "freeze-pump-thaw" cycle after which the atmosphere is exchanged with carbon dioxide. After five days during which the previously brown reaction mixture has turned red the solution is transferred to a vial and all volatiles are removed under reduced pressure. The red crude product is co-evaporated with diethylether (2x0.4 ml) and extracted with a 1:10 mixture of *o*-difluorobenzene and *n*-heptane. The filtered extract is layered with additional *n*-heptane and stored at room temperature to yield red crystals of $[(Me_3P)_2Ir(SnAr^*)_2(\mu_3\text{-}\eta^3\text{-}\kappa^3\text{-}O,C,O'\text{-}CO_2)_2][BAr^F_4]$ (35.2 mg, 14.1 µmol, 52%) suitable for X-ray diffraction. *Analytical data*: **^{1}H-NMR** [600.13 MHz, 298.0 K, benzene-d_6 + *o*-difluorobenzene]: δ (ppm) 0.97 (d, 24H, $^3J_{H\text{-}H}$ = 6.8 Hz, *o*-^{i}Pr-C<u>H</u>$_3$), 1.19–1.21 (d, 18H, P(C<u>H</u>$_3$)$_3$), 1.22 (d, 24H, $^3J_{H\text{-}H}$ = 6.9 Hz, *o*-^{i}Pr-C<u>H</u>$_3$), 1.28 (s, 24H, $^3J_{H\text{-}H}$ = 6.9 Hz, *p*-^{i}Pr-C<u>H</u>$_3$), 2.65 (sept, 8H, $^3J_{H\text{-}H}$ = 6.8 Hz, *o*-^{i}Pr-C<u>H</u>), 2.86 (sept, 4H, $^3J_{H\text{-}H}$ = 6.8 Hz, *p*-^{i}Pr-C<u>H</u>), 7.07–7.10 (m, 12H, 8x*m*-<u>H</u>$_{trip}$ + 4x*m*-C$_6$<u>H</u>$_3$), 7.29–7.32 (m, 2H, *p*-C$_6$<u>H</u>$_3$), 7.56 (br. s, 4H, *p*-BAr$^F_4$-<u>H</u>), 8.22–8.24 (br. m, 8H, *o*-BAr$^F_4$-<u>H</u>). **^{11}B-NMR** [96.29 MHz, 298.0 K, benzene-d_6 + *o*-difluorobenzene]: δ (ppm) −6.1 (s, <u>B</u>Ar$^F_4$). **^{13}C{^{1}H}-NMR** [150.93 MHz, 298.0 K, benzene-d_6 + *o*-difluorobenzene]: δ (ppm) 22.7–22.9 (m, P(<u>C</u>H$_3$)$_3$), 23.7 (s, *o*-^{i}Pr-<u>C</u>H$_3$), 24.0 (s, *p*-^{i}Pr-<u>C</u>H$_3$), 25.5 (s, *o*-^{i}Pr-<u>C</u>H$_3$), 31.5 (s, *o*-^{i}Pr-<u>C</u>H), 34.8 (s, *p*-^{i}Pr-<u>C</u>H), 117.9 (br. s, *p*-BAr$^F_4$-<u>C</u>), 121.9 (s, *m*-<u>C</u>$_{trip}$), 125.3 (q, $^1J_{F\text{-}C}$ = 272.0 Hz, <u>C</u>F$_3$), 130.0 (q, $^2J_{F\text{-}C}$ = 31.5 Hz, *m*-BAr$^F_4$-<u>C</u>), 130.7 (s, *m*-<u>C</u>$_6$H$_3$), 131.4 (s, *p*-<u>C</u>$_6$H$_3$), 135.4–135.5 (m, *i*-<u>C</u>$_{trip}$ + *o*-BAr$^F_4$-<u>C</u>), 142.0 (*i*-<u>C</u>$_6$H$_3$), 147.3 (s, *o*-<u>C</u>$_6$H$_3$), 148.4 (s, *o*-<u>C</u>$_{trip}$), 150.8 (s, *p*-<u>C</u>$_{trip}$), 162.9 (q, $^1J_{C\text{-}B}$ = 49.8 Hz, *i*-BAr$^F_4$-<u>C</u>), 199.2–200.0 (m, AA'X spin system, $^2J_{P\text{-}C}$ = 91.0 Hz, $^2J_{P\text{-}P'}$ = 23.6 Hz, $^2J_{P'\text{-}C}$ = −8.4 Hz, Ir–<u>C</u>). **^{19}F{^{1}H}-NMR** [282.37 MHz, 298.0 K, benzene-d_6 + *o*-difluorobenzene]: δ (ppm) −62.8 (s, C<u>F</u>$_3$). **^{31}P{^{1}H}-NMR** [121.49 MHz, 298.0 K, benzene-d_6 + *o*-difluorobenzene]: δ (ppm) −54.9 (s + sat. (Sn$_2$), 2P, $^2J_{119/117Sn\text{-}P}$ = 67.1 Hz, <u>P</u>(CH$_3$)$_3$). **^{119}Sn{^{1}H}-NMR** [223.68 MHz, 298.0 K, benzene-d_6 + *o*-difluorobenzene]: δ (ppm) −692.0 (br. t + sat., 2Sn, $^2J_{119Sn\text{-}117Sn}$ = 14466 Hz, $^2J_{Sn\text{-}P}$ = ca. 63 Hz). **IR (ATR, cm^{-1}):** 1221, 1242, 1279, 1301 (ν(C–O)). **UV/Vis** (*o*-difluorobenzene, c = 0.046 mmol·L^{-1}): λ (nm) (ε (L·mol^{-1}·cm^{-1})): 443 (11728), 372 (15491). **Elemental analysis** calculated for $C_{112}H_{128}BF_{24}IrO_4P_2Sn_2$ (%): C 53.88, H 5.17. Found: C 53.77, H 4.96.

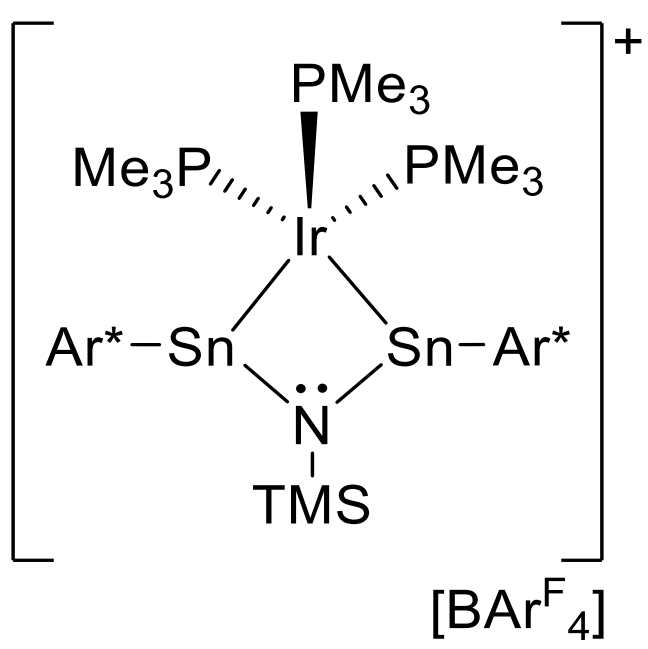

Compound **3**: $[(PMe_3)_2Ir(SnAr^*)_2][BAr^F_4]$ (38.1 mg, 15.8 µmol, 1.00 equiv.) is soluded in benzene (0.4 ml) and *o*-difluorobenzene (0.1 ml). The solution is treated with trimethylphosphine (9.78 µl, 94.9 mmol, 6.00 equiv.) and afterwards with trimethylsilyl azide (33.6 µl of a 10 vol-% solution in benzene, 25.3 µmol, 1.60 equiv.) at room temperature. After one hour during which the previously brown reaction mixture turns olive green all volatiles are removed under reduced pressure and the residue is co-evaporated and afterwards washed with *n*-pentane (2 x 0.3 ml + 3 x 0.3 ml). The dried, olive green crude product is crystallized from a saturated *o*-difluorobenzene solution containing one drop of diethyl ether, layered with *n*-hexane at −35 °C to yield olive green crystals of $[(Me_3P)_3Ir(SnAr^*)_2(\mu_2\text{-N–TMS})][BAr^F_4] \cdot C_6H_{14}$ (26.0 mg, 9.78 µmol, 62%) suitable for X-ray. *Analytical data*: **^{1}H-NMR** [600.13 MHz, 273.0 K, toluene-d_8 + *o*-difluorobenzene]: δ (ppm) −0.63 (br. s, 9H, Si(CH$_3$)$_3$), 0.83–0.91 (br. m, 12H, *o*-^{i}Pr-C$\underline{H}_3$), 0.91–1.00 (br. m, 39H, 27 x P(CH$_3$)$_3$ + 12 x *o*-^{i}Pr-C$\underline{H}_3$), 1.19 (br. d, 12H, $^3J_{H\text{-}H}$ = 6.3 Hz, *o*-^{i}Pr-C$\underline{H}_3$), 1.26 (d, 24H, $^3J_{H\text{-}H}$ = 6.9 Hz, *p*-^{i}Pr-C$\underline{H}_3$), 1.31 (br. d, 12H, $^3J_{H\text{-}H}$ = 6.6 Hz, *o*-^{i}Pr-C$\underline{H}_3$), 2.31–2.75 (br. m, 4H, *o*-^{i}Pr-C$\underline{H}$), 2.78–2.92 (m, 8H, 4 x *o*-^{i}Pr-C$\underline{H}$ + 4 x *p*-^{i}Pr-C$\underline{H}$), 6.98–7.01 (m, 2H, *p*-C$_6\underline{H}_3$), 7.03–7.06 (br. m, 4H, *m*-C$_6\underline{H}_3$, partially overlapping with toluene-d_8, found in 2D NMR), 7.09–7.14 (br. m, 8H, *m*-$\underline{H}_{trip}$), 7.52 (br. s, 4H, *p*-BAr$^F_4$-$\underline{H}$), 8.23–8.25 (br. m, 8H, *o*-BAr$^F_4$-$\underline{H}$). **^{11}B{^{1}H}-NMR** [192.55 MHz, 273.0 K, toluene-d_8 + *o*-difluorobenzene]: δ (ppm) −6.0 (s, $\underline{B}$Ar$^F_4$). **^{13}C{^{1}H}-NMR** [150.92 MHz, 273.0 K, toluene-d_8 + *o*-difluorobenzene]: δ (ppm) 4.8 (br. s, Si(CH$_3$)$_3$), 23.7 (s, *p*-^{i}Pr-$\underline{C}$H$_3$), 23.8 (br. s, *o*-^{i}Pr-$\underline{C}$H$_3$), 24.0 (s, *p*-^{i}Pr-$\underline{C}$H$_3$), 25.6 (s, *o*-^{i}Pr-$\underline{C}$H$_3$), 26.1 (s, *o*-^{i}Pr-$\underline{C}$H$_3$), 29.3–29.5 (br. m, P($\underline{C}$H$_3$)$_3$), 30.4 (s, *o*-^{i}Pr-$\underline{C}$H), 30.7 (s, *o*-^{i}Pr-$\underline{C}$H), 34.5 (s, *p*-^{i}Pr-$\underline{C}$H), 117.5 (s, *p*-BAr$^F_4$-$\underline{C}$), 122.1 (s, *m*-$\underline{C}_{trip}$), 122.3 (s, *m*-$\underline{C}_{trip}$), 124.8 (q, $^1J_{F\text{-}C}$ = 272.9 Hz, $\underline{C}$F$_3$), 127.5 (br. s, *p*-$\underline{C}_6$H$_3$, overlapping with toluene-d_8, found in 2D NMR), 129.5 (q, $^2J_{F\text{-}C}$ = 30.9 Hz, *m*-BAr$^F_4$-$\underline{C}$), 135.0 (s, *o*-BAr$^F_4$-$\underline{C}$), 135.3 (s, *m*-$\underline{C}_6$H$_3$), 137.2 (s, *i*-$\underline{C}_{trip}$, overlapping with toluene-d_8, found in 2D NMR), 144.6 (s, *o*-$\underline{C}_6$H$_3$), 147.1 (s, *o*-$\underline{C}_{trip}$), 149.7 (s, *p*-$\underline{C}_{trip}$, partially overlapping with toluene-d_8, found in 2D NMR), 162.4 (q, $^1J_{C\text{-}B}$ = 49.4 Hz, *i*-BAr$^F_4$-$\underline{C}$), 196.1 (br. s, *i*-$\underline{C}_6$H$_3$). **^{19}F-NMR** [564.63 MHz, 273.0 K, toluene-d_8 + *o*-difluorobenzene]: δ (ppm) −62.4 (s, C$\underline{F}_3$). **^{29}Si{^{1}H}-NMR** [119.23 MHz, 273.0 K, toluene-d_8 + *o*-difluorobenzene]: δ (ppm) 1.2 (s, $\underline{Si}$(CH$_3$)$_3$). **^{31}P{^{1}H}-NMR** [121.49 MHz, 298.0 K, toluene-d_8 + *o*-difluorobenzene]: δ (ppm) −52.2 (s + sat. (Sn$_2$), 3P, $^2J_{119/117Sn\text{-}P}$ = 491.1 Hz, $\underline{P}$(CH$_3$)$_3$). **^{119}Sn{^{1}H}-NMR**: [112.03 MHz, 298.0 K, benzene-d_6 + *o*-difluorobenzene]: δ (ppm) 619.2 (br. s). **UV/Vis** (*o*-difluorobenzene, c = 0.055 mmol·L^{-1}): λ (nm) (ε (L·mol^{-1}·cm^{-1})): 680 (2899), 448 (7475). **Elemental analysis** calculated for $C_{116}H_{146}BF_{24}IrNP_3SiSn_2$ + C_6H_{14} (%): C 55.13, H 6.07, N 0.53. Found: C 55.22, H 5.76, N 0.56.

Data of crystal structure determinations

Table SI1. Crystal structure refinement data of **1a**, **2** and **3**.

	**1a**	**2**	**3**
Empirical formula	$C_{101.5}H_{134}AlF_{36}IrOP_2Sn_2$	$C_{96}H_{116}AlF_{36}IrO_8P_2Sn_2$	$C_{122}H_{160}BF_{24}IrNP_3SiSn_2$
M_r / g mol^{-1}	2620.58	2600.38	2657.89
l / Å	0.71073	1.54184	0.71073
T / K	100(2)	150(2)	100(2)
Crystal system	triclinic	triclinic	triclinic
Space group	$P\bar{1}$	$P\bar{1}$	$P\bar{1}$
Z	2	2	2
a / Å	15.1347(3)	15.35331(9)	14.8154(9)
b / Å	18.2776(4)	20.39448(17)	15.3530(9)
c / Å	22.6026(5)	20.99026(13)	27.6676(16)
a / °	81.6930(10)	78.3043(6)	87.187(3)
b / °	72.7950(10)	80.1934(5)	79.761(3)
g / °	81.0720(10)	84.8547(6)	85.538(3)
V / Å^3	5868.0(2)	6331.66(8)	6170.3(6)
D_c / g cm^{-3}	1.483	1.364	1.431
m / mm^{-1}	1.691	6.275	1.609
F(000)	2638	2600	2712
Crystal size / mm	0.31 x 0.27 x 0.25	0.22 x 0.21 x 0.19	0.29 x 0.27 x 0.26
q range / °	1.469 - 26.373	2.176 - 79.728	1.400 - 32.275
Limiting indices	−18 ≤ h ≤ 18<br>−22 ≤ k ≤ 22<br>−28 ≤ l ≤ 28	−19 ≤ h ≤ 17<br>−25 ≤ k ≤ 26<br>−26 ≤ l ≤ 26	−22 ≤ h ≤ 22<br>−23 ≤ k ≤ 22<br>−41 ≤ l ≤ 41
Reflections collect.	133890	163335	447471
Indepdnt. Reflections	23932	27177	43282
R_{int}	0.0289	0.0427	0.0382
Completeness	0.998	0.986	0.987
Absorp. Corr.	multi-scan	multi-scan	multi-scan
Trans. (max., min.)	0.7461, 0.6806	1.00000, 0.63129	0.7463, 0.6676
Parameters/restraints	1506/1066	1691/6306	1491/249
R_1, wR_2 [I > 2s(I)]	0.0271, 0.0628	0.0500, 0.1282	0.0313, 0.0676
R_1, wR_2 (all data)	0.0350, 0.0670	0.0545, 0.1326	0.0423, 0.0740
GooF on F^2	1.042	1.020	1.093
$Dr_{max,min}$ / e · Å^{-3}	1.894, −1.309	2.694, −1.883	2.931, −1.397
CCDC	2562466	2562465	2562467

NMR-Spectroscopy

NMR spectra of compound **1a**

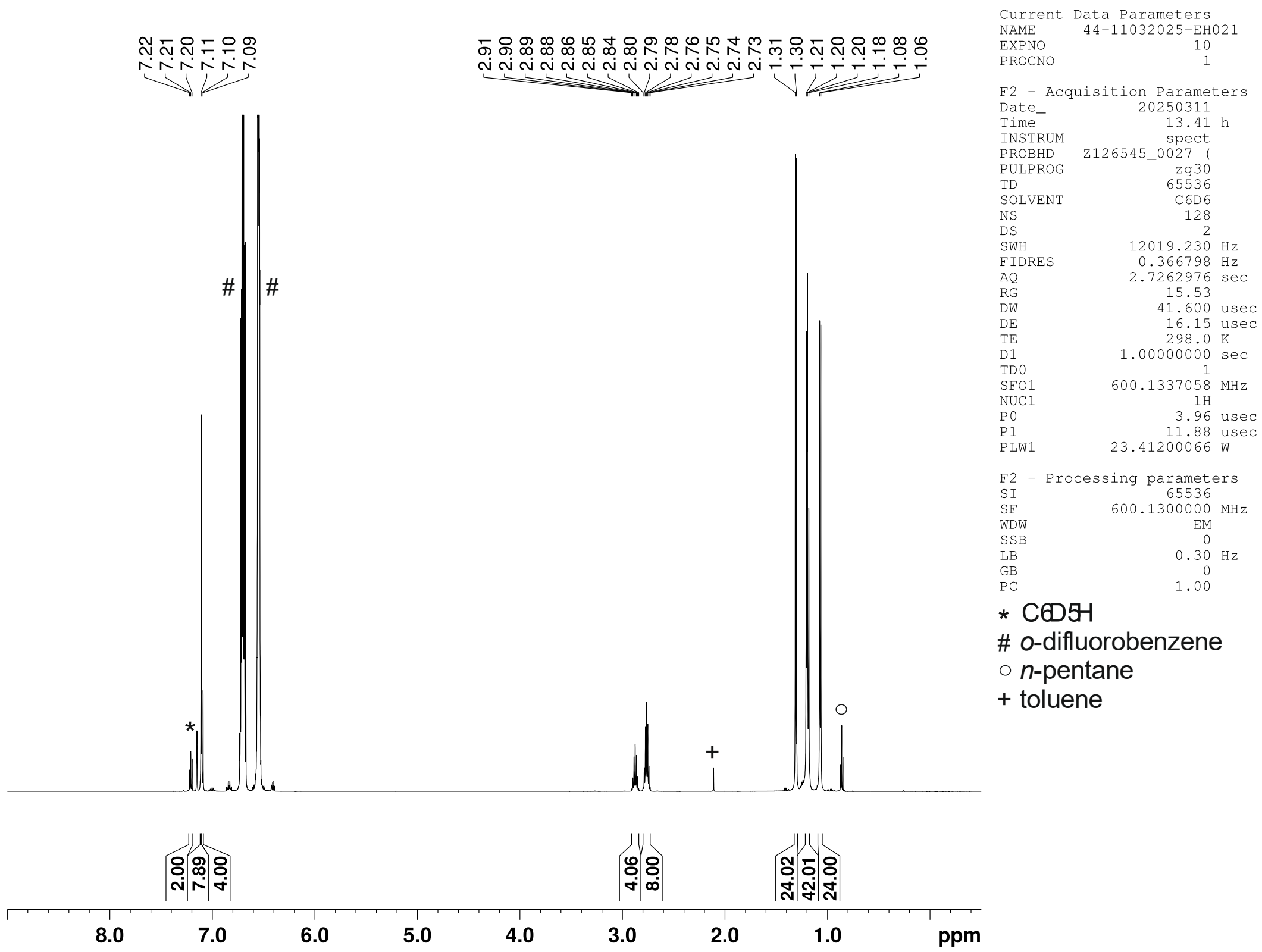

Figure SI1. ^{1}H NMR of compound **1a** (0.5 – 9 ppm).

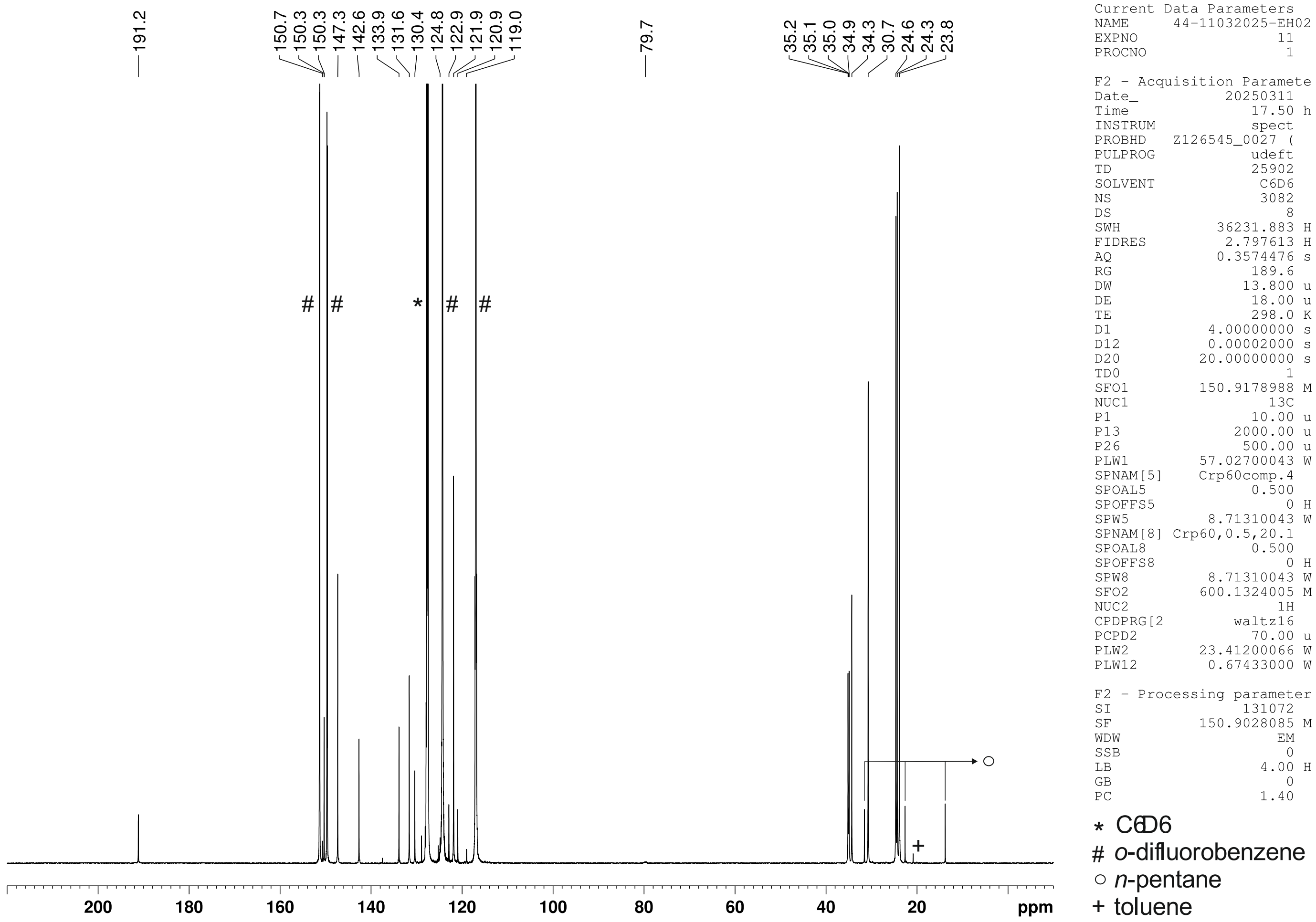

Figure SI2. $^{13}C\{^1H\}$ NMR of compound **1a**.

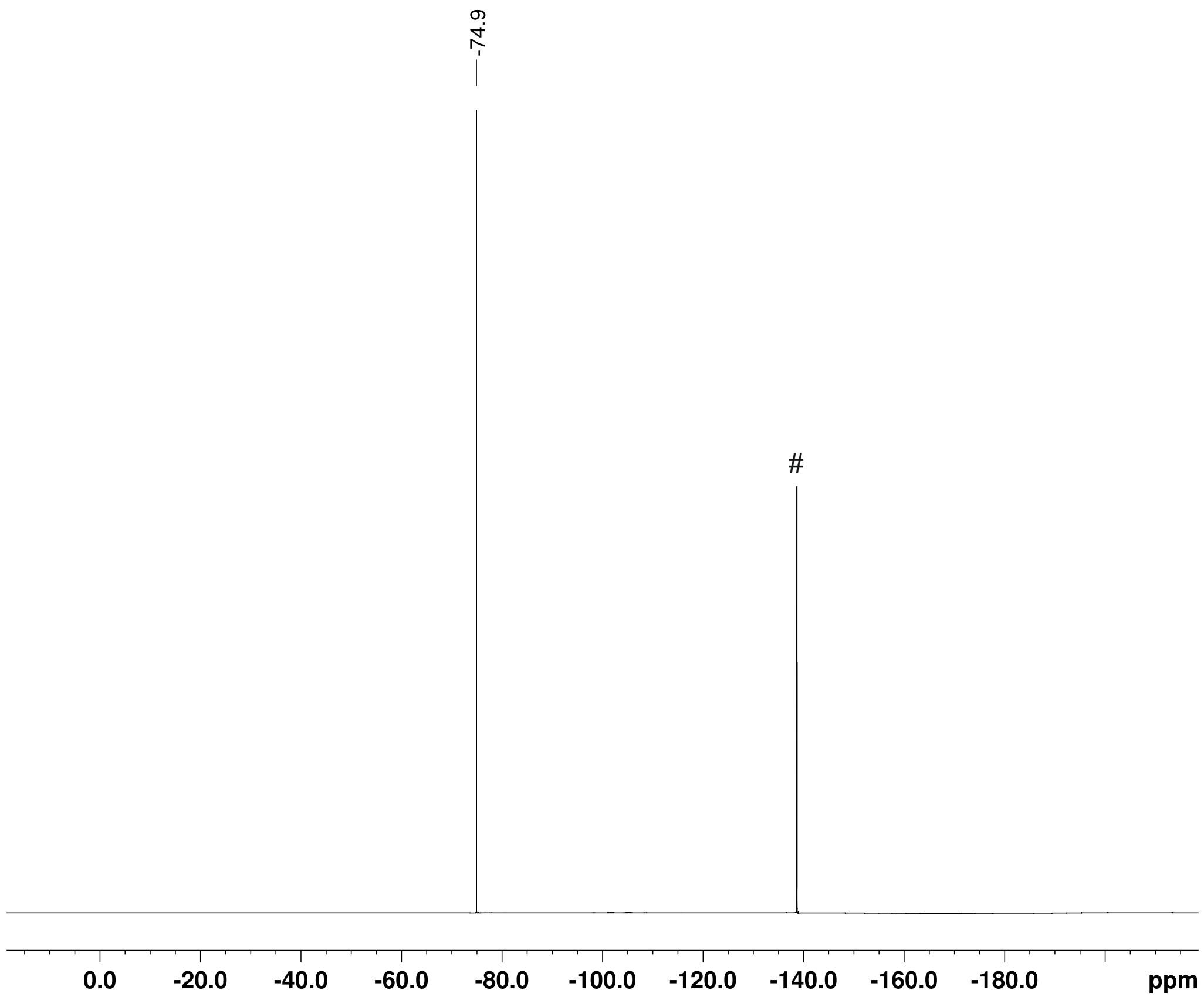


```
Current Data Parameters
NAME     44-11032025-EH021
EXPNO                   16
PROCNO                   1

F2 - Acquisition Parameters
Date_          20250312
Time               1.56 h
INSTRUM           spect
PROBHD   Z126545_0027 (
PULPROG          zgflqn
TD               131072
SOLVENT            C6D6
NS                  128
DS                    4
SWH          133928.578 Hz
FIDRES         2.043588 Hz
AQ            0.4893355 sec
RG                37.92
DW                3.733 usec
DE                18.00 usec
TE                298.0 K
D1           1.00000000 sec
TD0                   1
SFO1        564.6299196 MHz
NUC1                19F
P1                16.00 usec
PLW1        25.98800087 W

F2 - Processing parameters
SI                65536
SF          564.6863882 MHz
WDW                  EM
SSB                   0
LB                 0.30 Hz
GB                    0
PC                 1.00
```

o-difluorobenzene

Figure SI3. $^{19}F\{^1H\}$ NMR of compound **1a**.

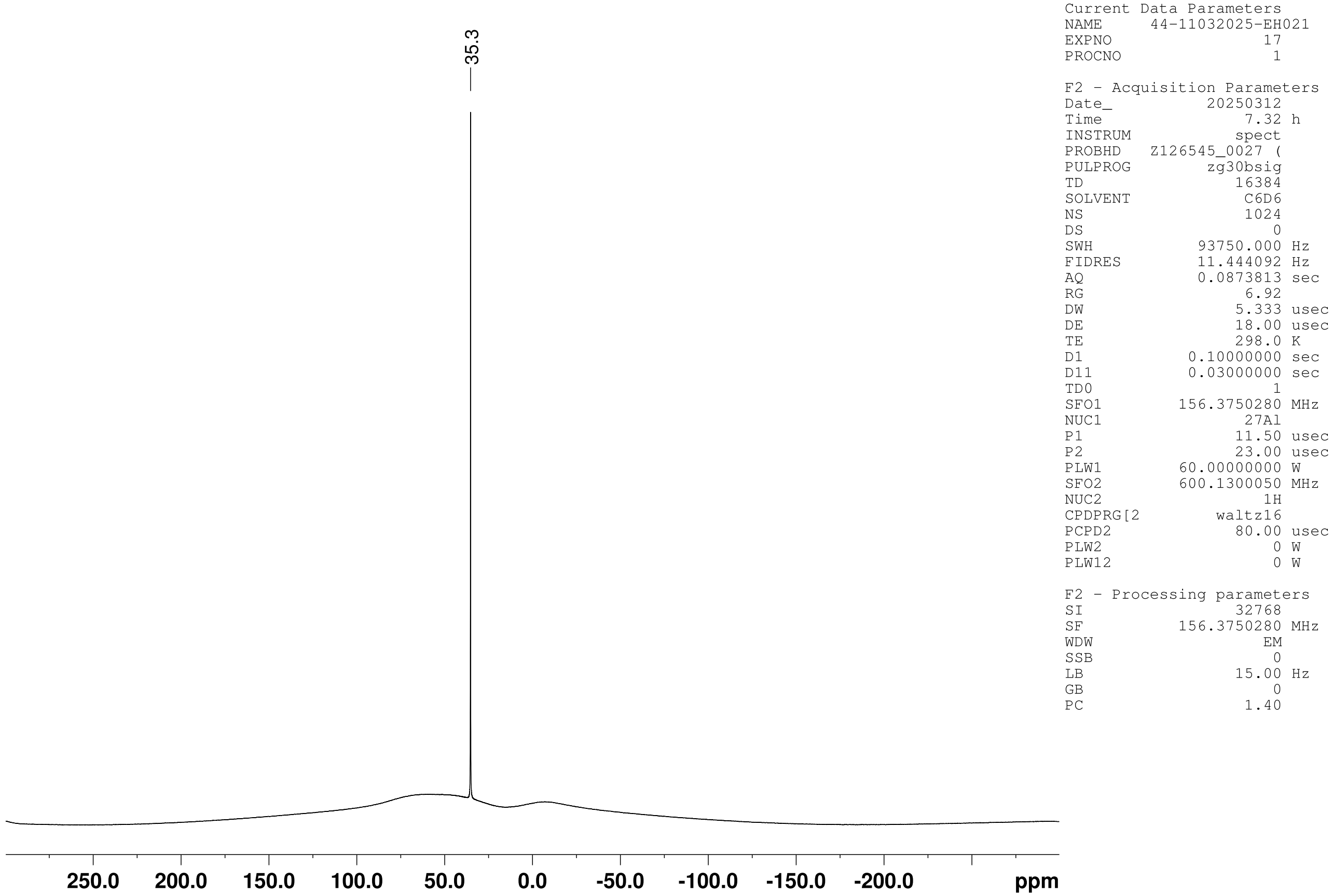

Figure SI4. $^{27}Al\{^{1}H\}$ NMR of compound **1a**.

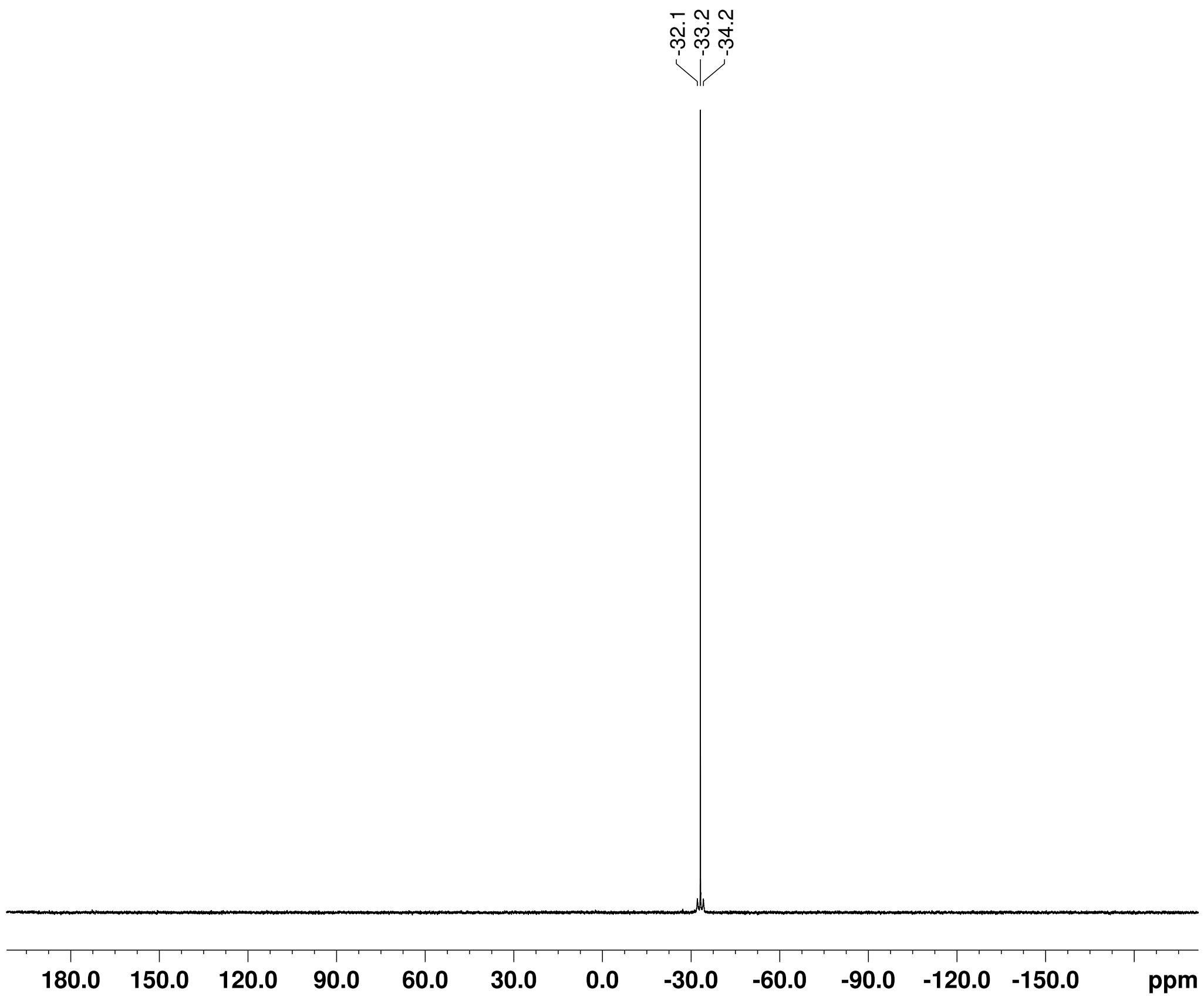


```
Current Data Parameters
NAME            EH021_300
EXPNO                  31
PROCNO                  1

F2 - Acquisition Parameters
Date_            20250310
Time                16.04 h
INSTRUM             spect
PROBHD   Z104275_0338 (
PULPROG           zgig30
TD                 78426
SOLVENT             C6D6
NS                   256
DS                     0
SWH            49019.609 Hz
FIDRES          1.250086 Hz
AQ             0.7999452 sec
RG                204.67
DW                10.200 usec
DE                  6.50 usec
TE                 298.0 K
D1            1.00000000 sec
D11           0.03000000 sec
TD0                    1
SFO1         121.4948510 MHz
NUC1                 31P
P0                  4.00 usec
P1                 12.00 usec
PLW1         11.36400032 W
SFO2         300.1314106 MHz
NUC2                  1H
CPDPRG[2         waltz16
PCPD2              90.00 usec
PLW2          8.26509953 W
PLW12         0.20000000 W

F2 - Processing parameters
SI                 65536
SF           121.4948510 MHz
WDW                   EM
SSB                    0
LB                  3.00 Hz
GB                     0
PC                  1.40
```

Figure SI5. $^{31}P\{^{1}H\}$ NMR of compound **1a**.

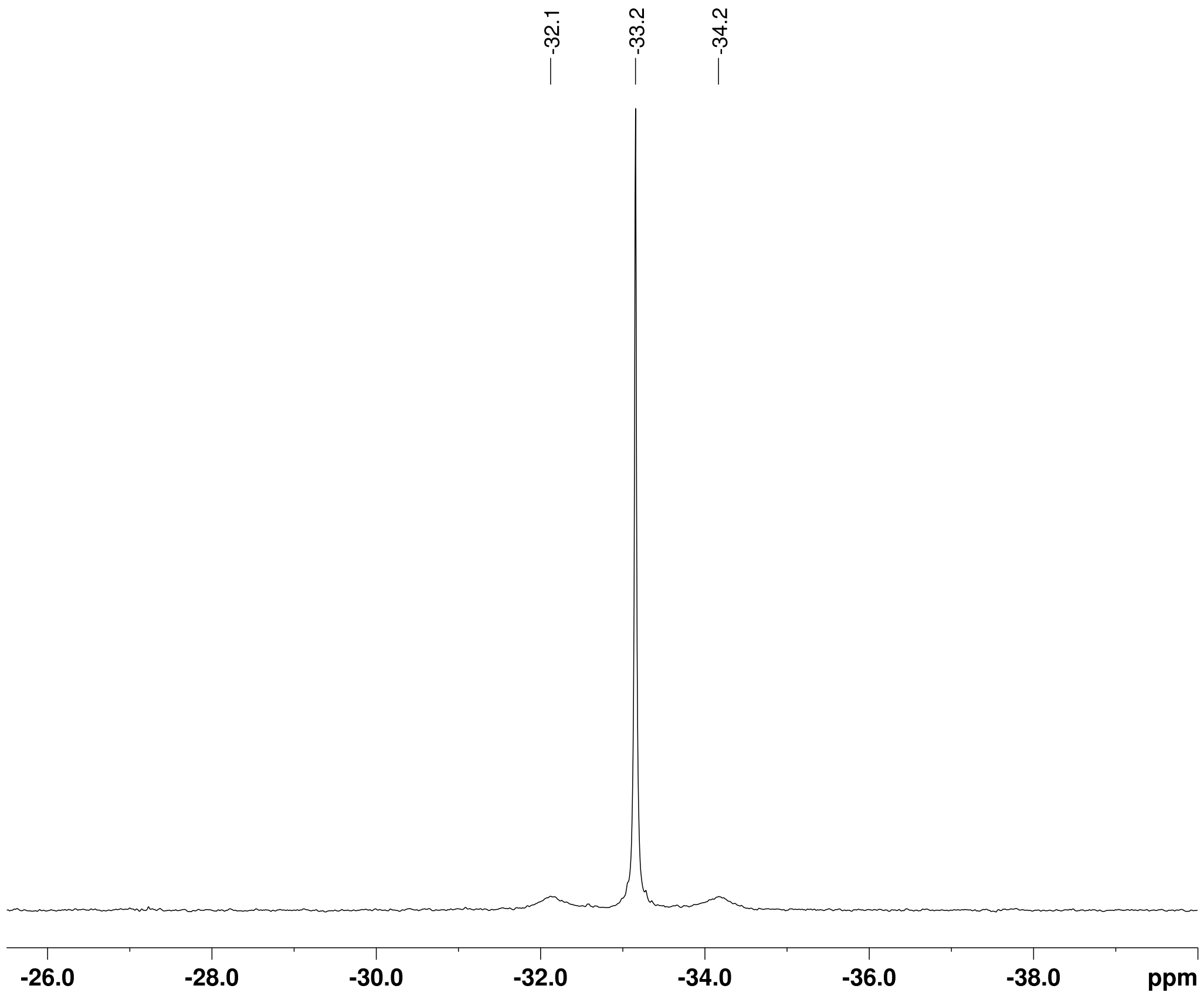


```
Current Data Parameters
NAME           EH021_300
EXPNO                 31
PROCNO                 1

F2 - Acquisition Parameters
Date_           20250310
Time               16.04 h
INSTRUM            spect
PROBHD   Z104275_0338 (
PULPROG           zgig30
TD                 78426
SOLVENT             C6D6
NS                   256
DS                     0
SWH            49019.609 Hz
FIDRES          1.250086 Hz
AQ             0.7999452 sec
RG                204.67
DW                10.200 usec
DE                  6.50 usec
TE                 298.0 K
D1            1.00000000 sec
D11           0.03000000 sec
TD0                    1
SFO1         121.4948510 MHz
NUC1                 31P
P0                  4.00 usec
P1                 12.00 usec
PLW1         11.36400032 W
SFO2         300.1314106 MHz
NUC2                  1H
CPDPRG[2         waltz16
PCPD2              90.00 usec
PLW2          8.26509953 W
PLW12         0.20000000 W

F2 - Processing parameters
SI                 65536
SF           121.4948510 MHz
WDW                   EM
SSB                    0
LB                  3.00 Hz
GB                     0
PC                  1.40
```

Figure SI6. $^{31}P\{^{1}H\}$ NMR of compound **1a** (−26 to −38 ppm).

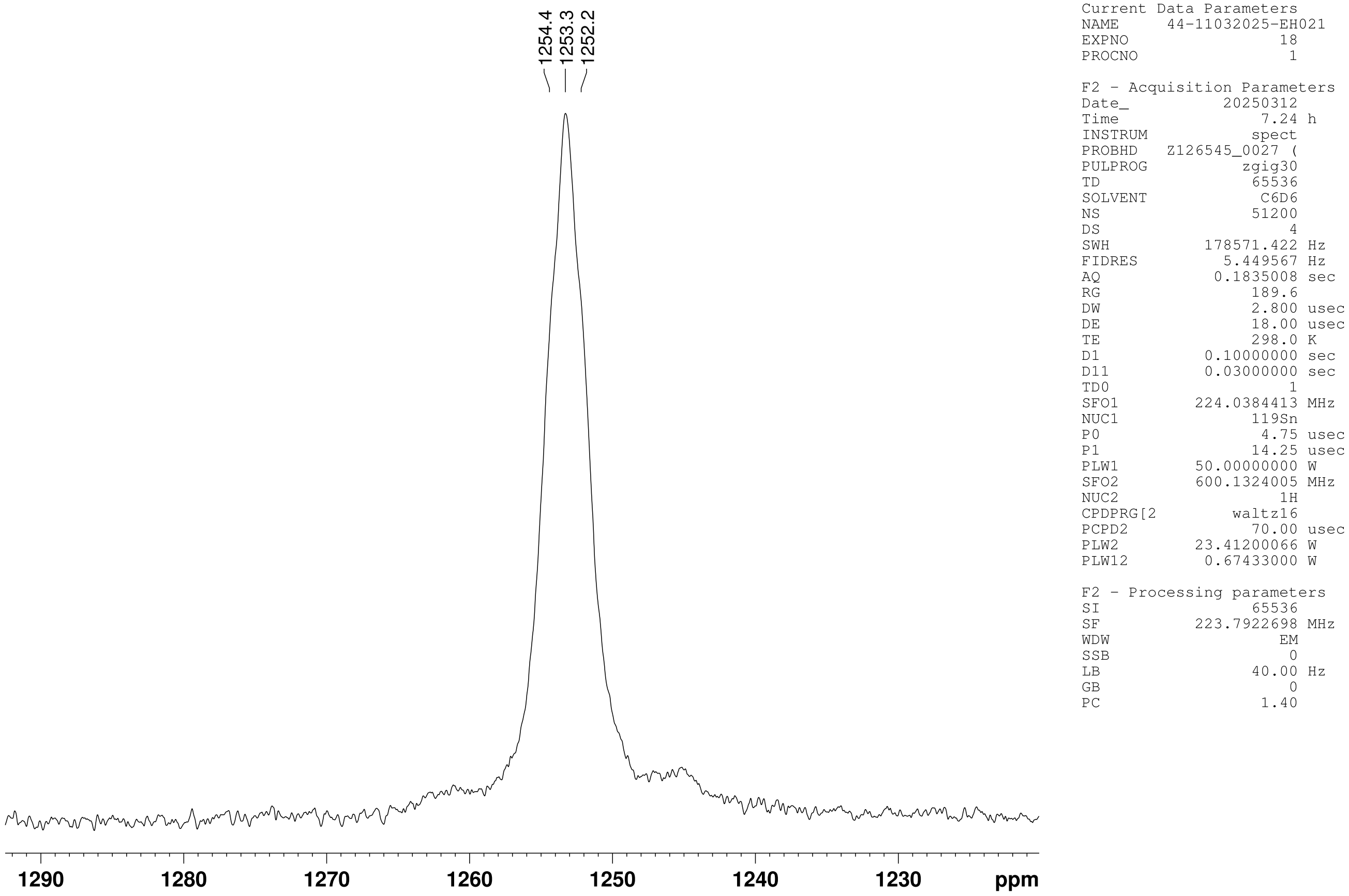

Figure SI7. $^{119}Sn\{^{1}H\}$ NMR of compound **1a**.

NMR spectra of compound **2**

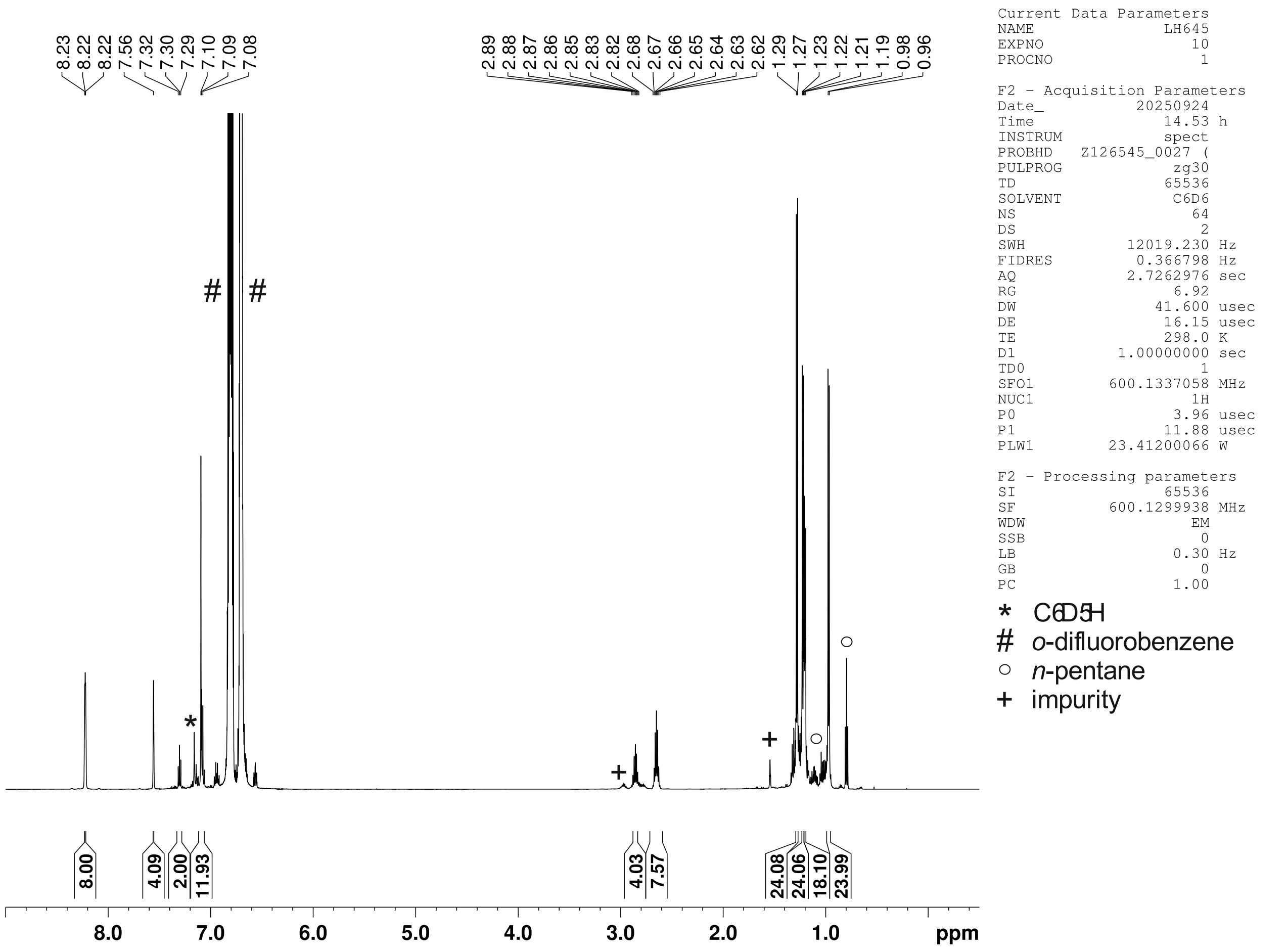

Figure SI8. ^{1}H NMR of compound **2** (0.5 – 9 ppm).

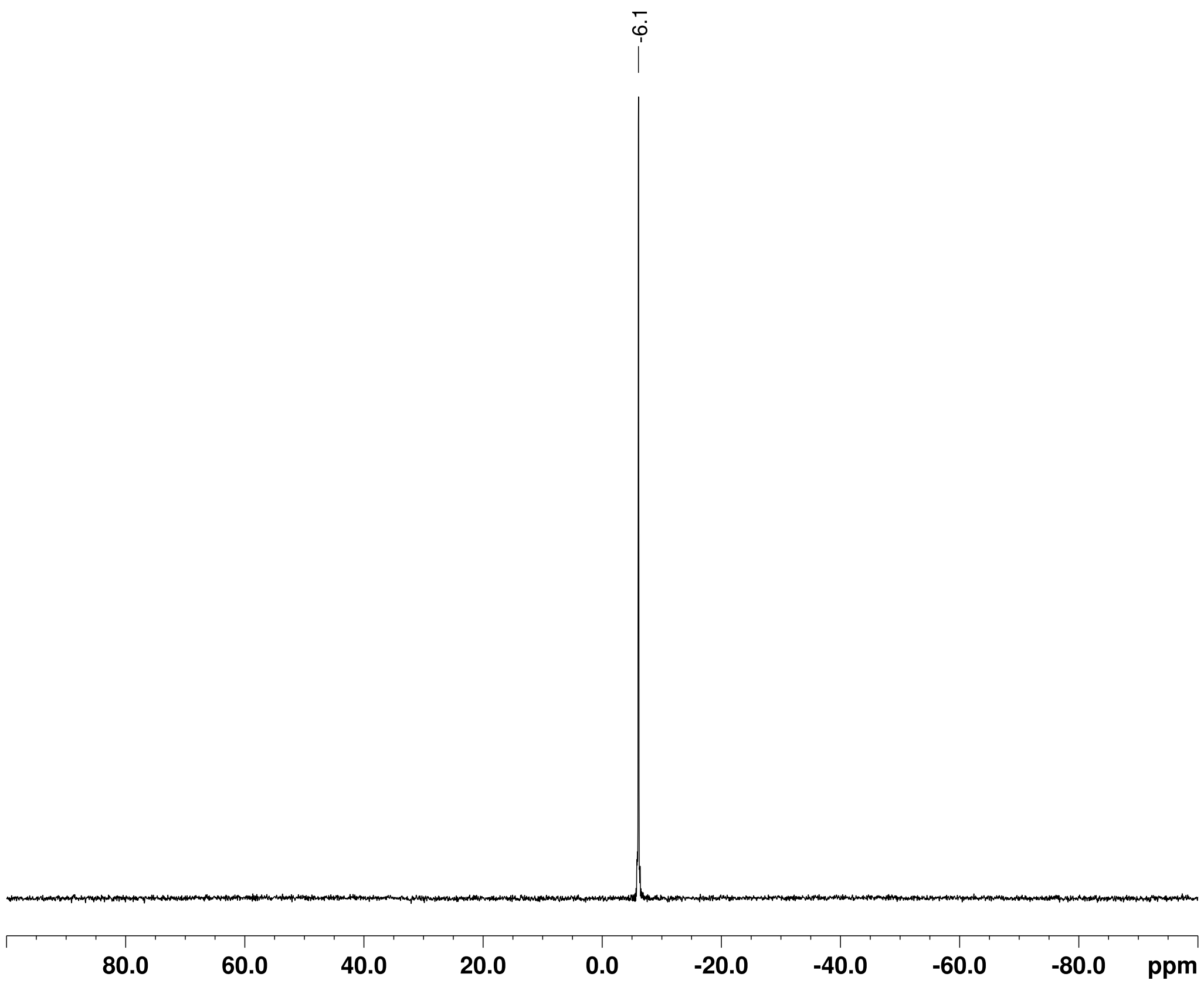


```
Current Data Parameters
NAME            LH645_300
EXPNO                  51
PROCNO                  1

F2 - Acquisition Parameters
Date_            20250926
Time                13.04 h
INSTRUM             spect
PROBHD   Z104275_0338 (
PULPROG              zgbs
TD                   8192
SOLVENT              C6D6
NS                   2048
DS                      0
SWH             48076.922 Hz
FIDRES          11.737530 Hz
AQ              0.0851968 sec
RG                 204.67
DW                 10.400 usec
DE                   6.50 usec
TE                  298.0 K
D1             0.10000000 sec
TD0                     1
SFO1           96.2936312 MHz
NUC1                  11B
P1                   5.75 usec
P2                  11.50 usec
PLW1          70.00000000 W

F2 - Processing parameters
SI                  32768
SF             96.2936312 MHz
WDW                    EM
SSB                     0
LB                   1.00 Hz
GB                      0
PC                   1.40
```

Figure SI9. $^{11}B\{^1H\}$ NMR of compound **2**.

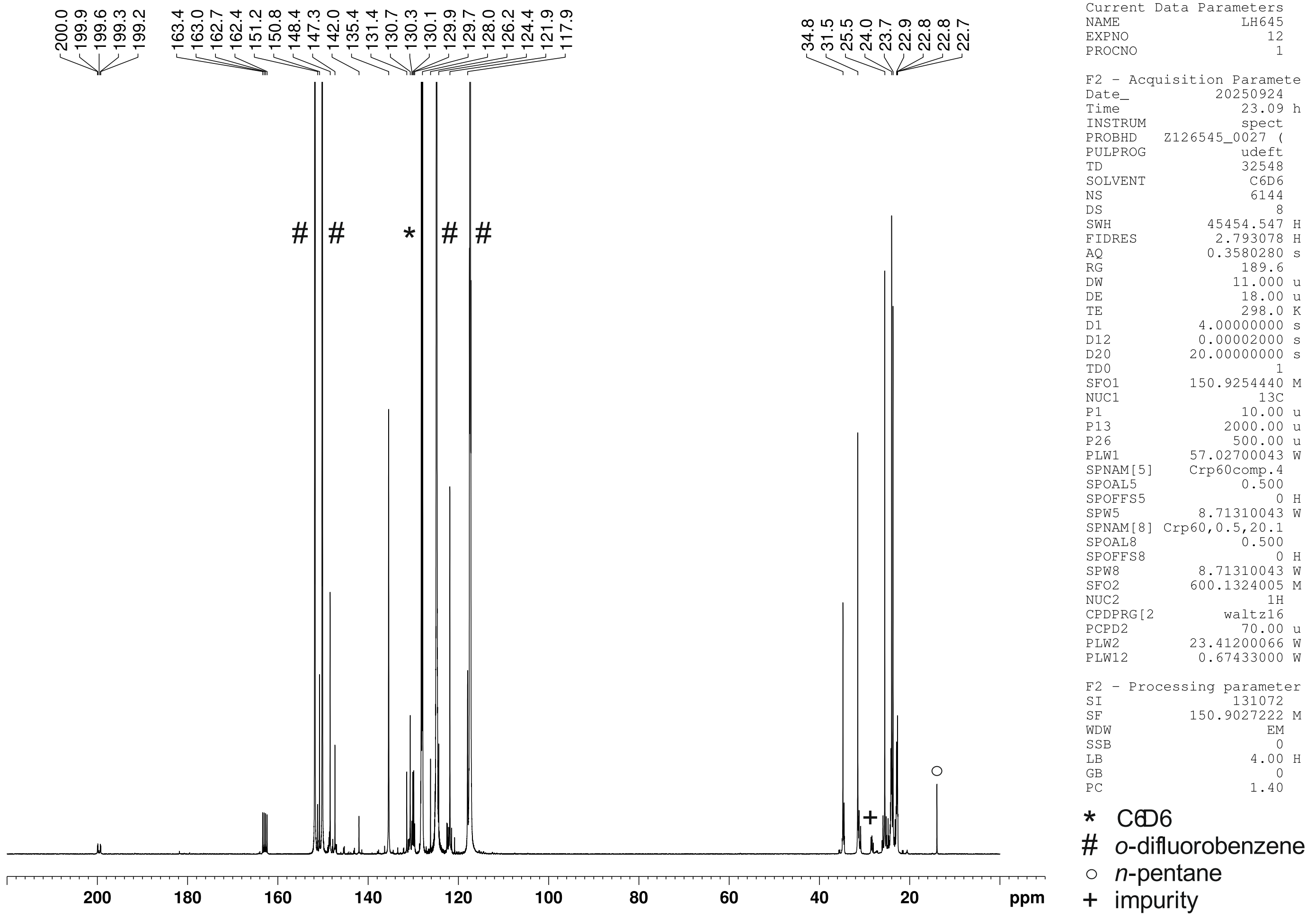

Figure SI10. $^{13}C\{^{1}H\}$ NMR of compound **2**.

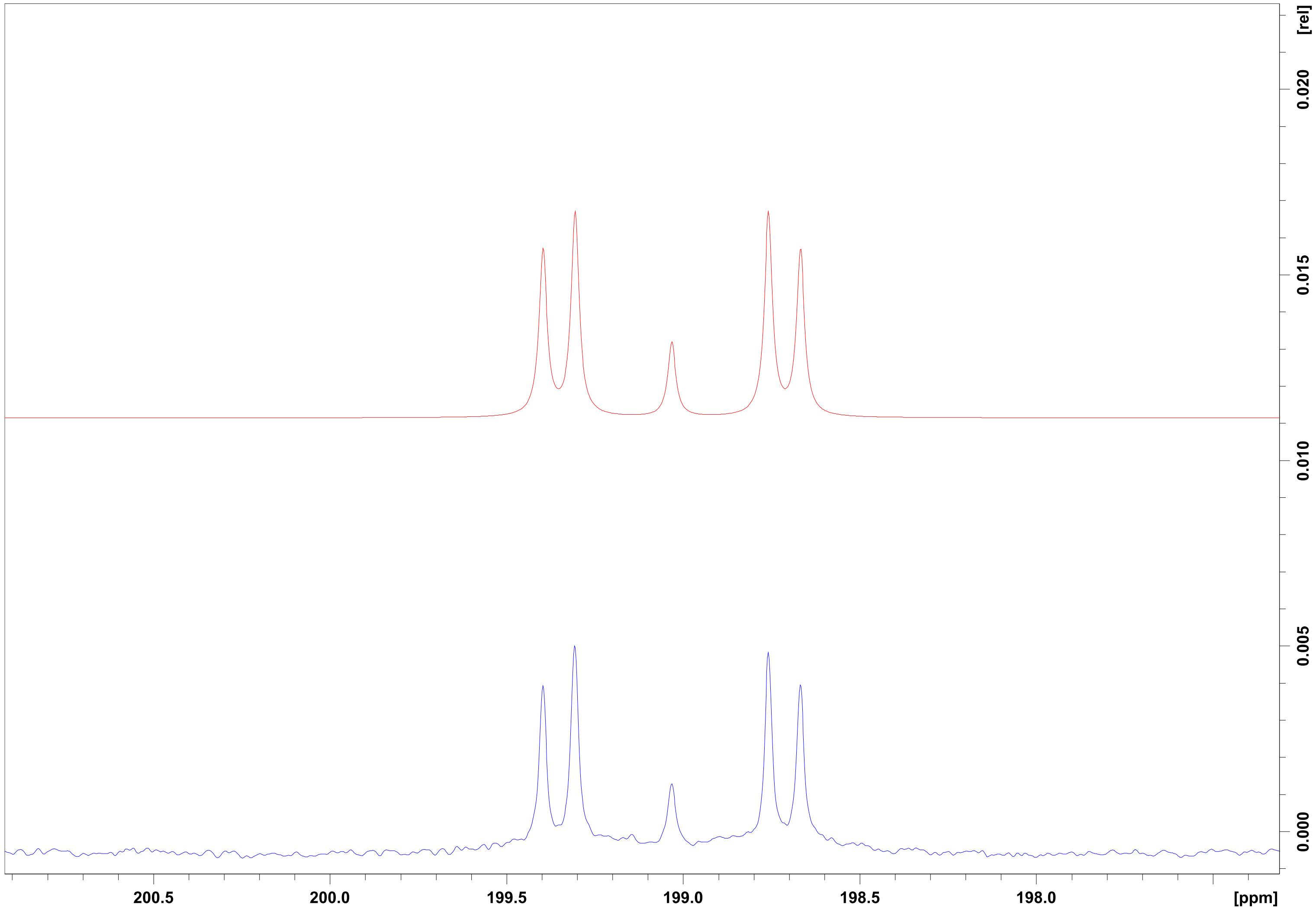

Figure SI11. $^{13}C\{^{1}H\}$ NMR of compound **2** at 199 ppm simulated (top) and experimental spectrum (bottom).

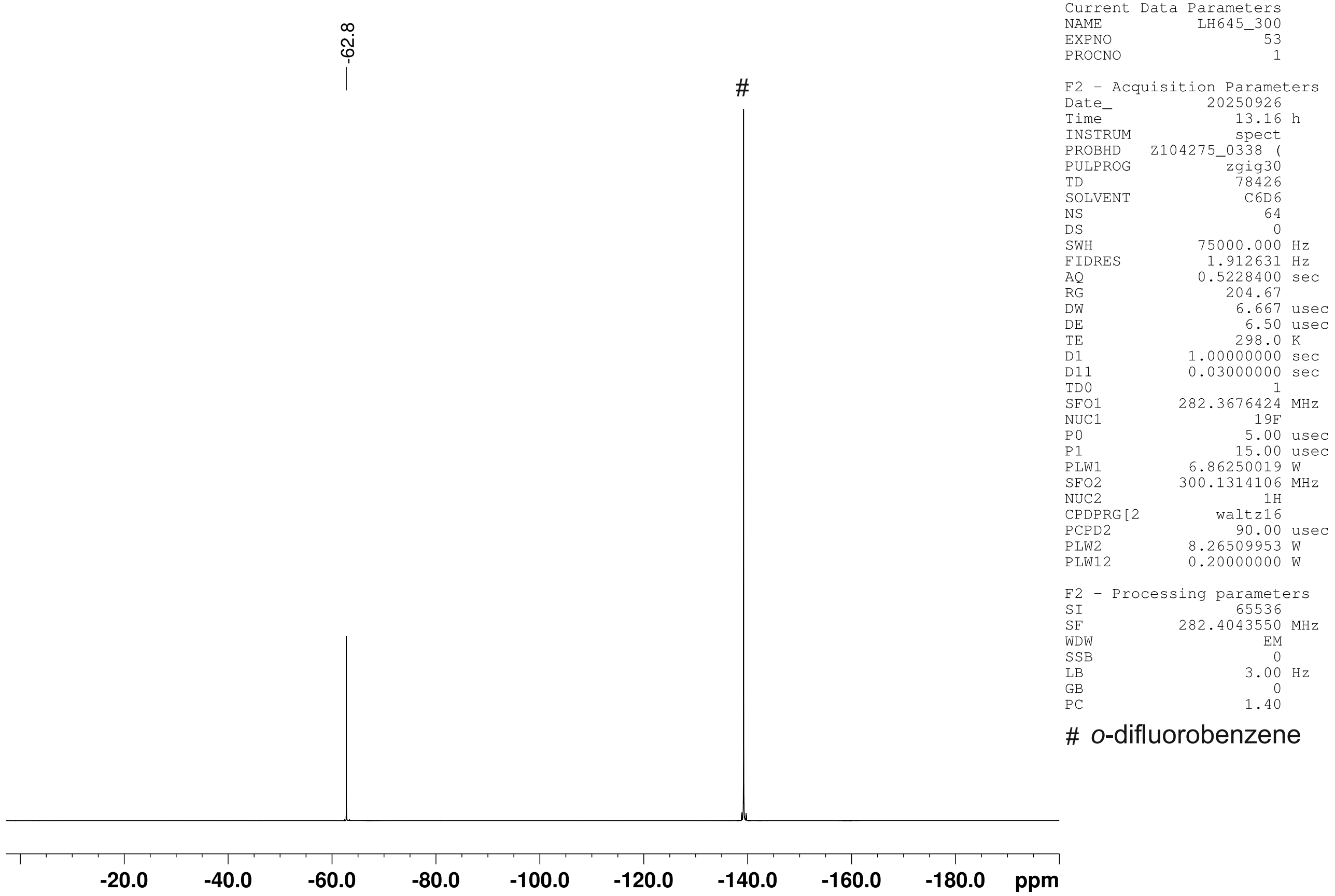

Figure SI12. $^{19}F\{^1H\}$ NMR of compound **2**.

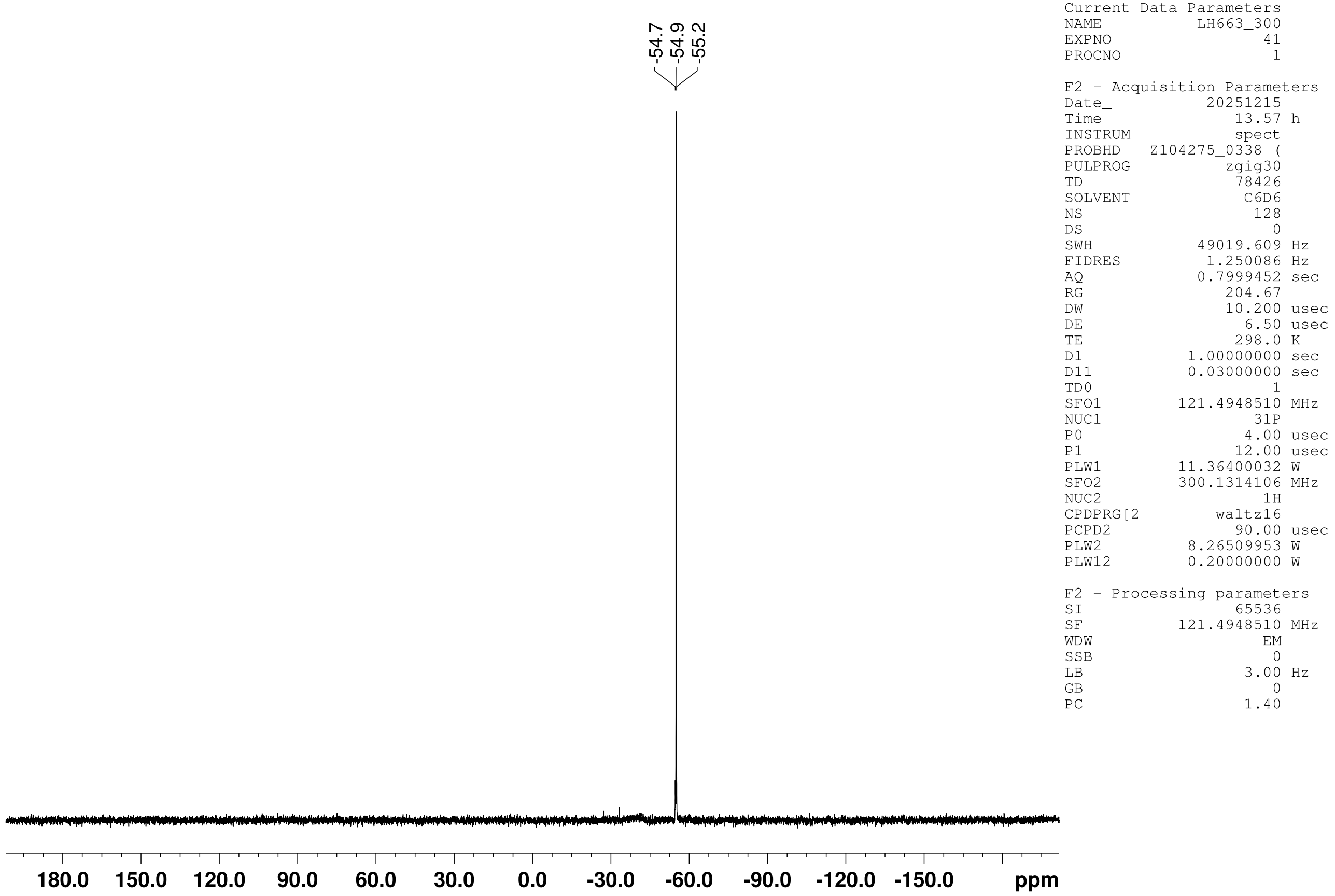

Figure SI13. $^{31}P\{^{1}H\}$ NMR of compound **2**.

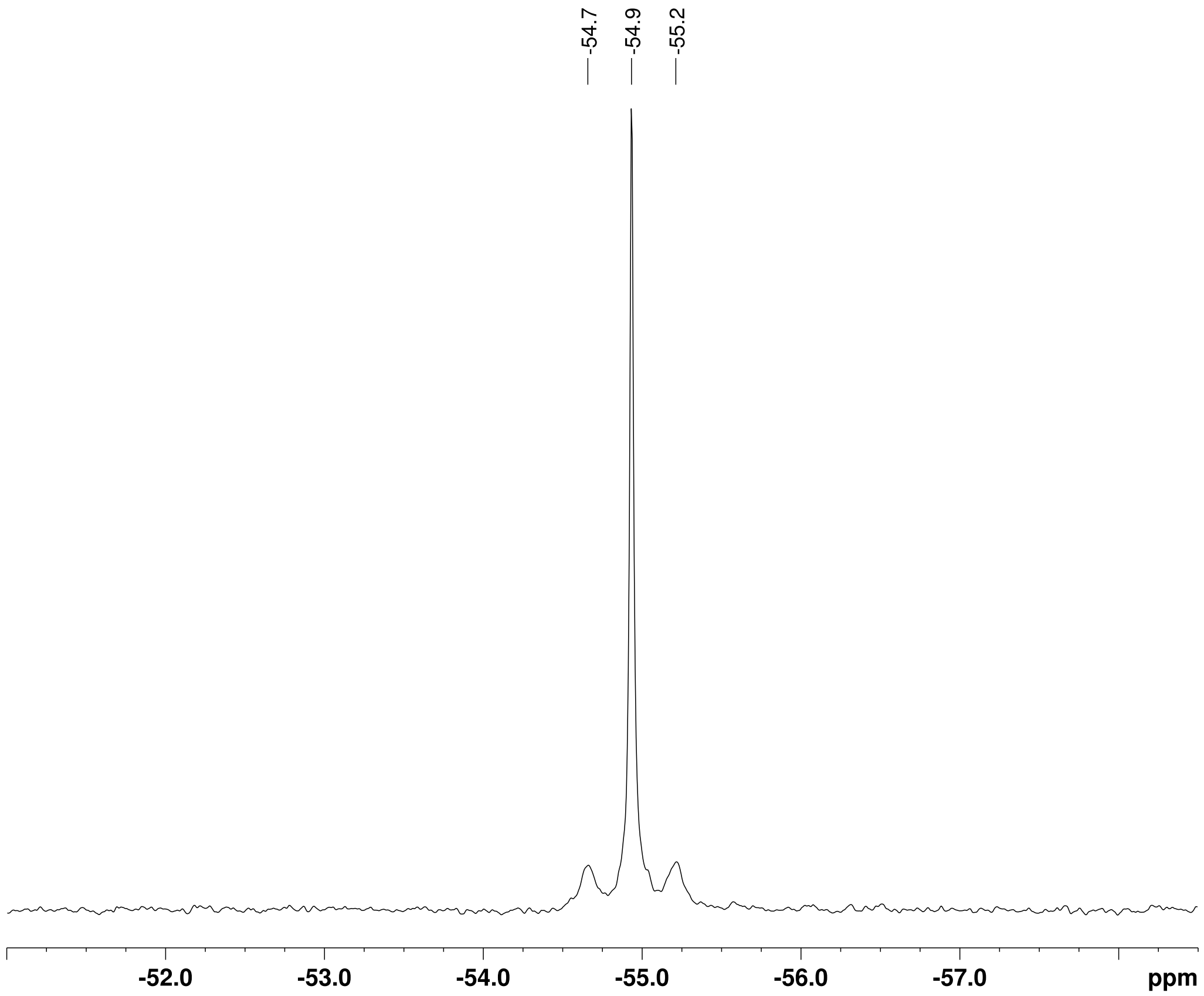


```
Current Data Parameters
NAME          LH663_300
EXPNO                41
PROCNO                1

F2 - Acquisition Parameters
Date_          20251215
Time              13.57 h
INSTRUM           spect
PROBHD   Z104275_0338 (
PULPROG          zgig30
TD                78426
SOLVENT            C6D6
NS                  128
DS                    0
SWH           49019.609 Hz
FIDRES         1.250086 Hz
AQ            0.7999452 sec
RG               204.67
DW               10.200 usec
DE                 6.50 usec
TE                298.0 K
D1           1.00000000 sec
D11          0.03000000 sec
TD0                   1
SFO1        121.4948510 MHz
NUC1                31P
P0                 4.00 usec
P1                12.00 usec
PLW1        11.36400032 W
SFO2        300.1314106 MHz
NUC2                 1H
CPDPRG[2        waltz16
PCPD2             90.00 usec
PLW2         8.26509953 W
PLW12        0.20000000 W

F2 - Processing parameters
SI                65536
SF          121.4948510 MHz
WDW                  EM
SSB                   0
LB                 3.00 Hz
GB                    0
PC                 1.40
```

Figure SI14. $^{31}P\{^{1}H\}$ NMR of compound **2** (−52 to −57 ppm).

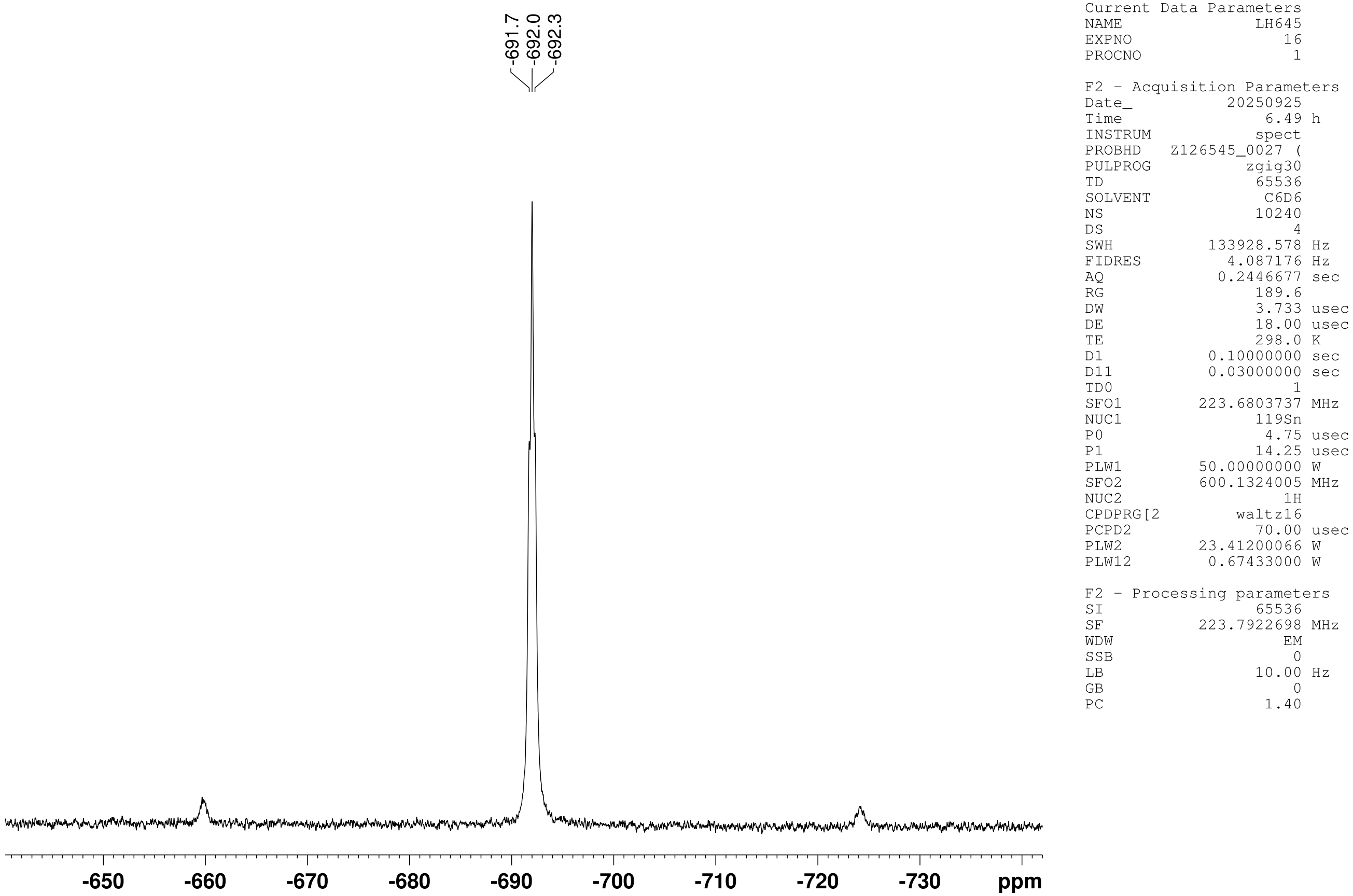

Figure SI15. $^{119}Sn\{^{1}H\}$ NMR of compound **2**.

NMR spectra of compound **3**

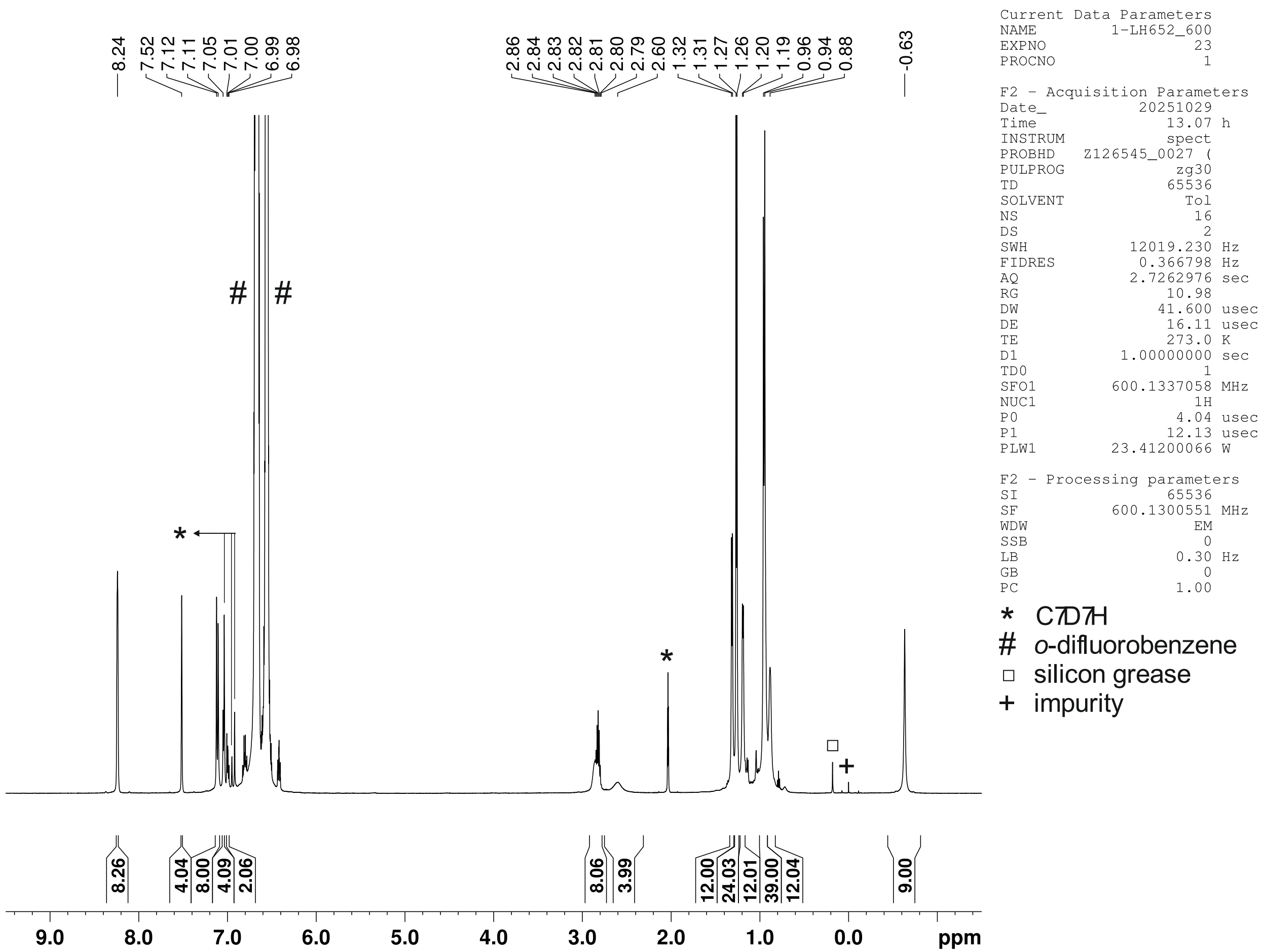

Figure SI16. ^{1}H NMR of compound **3** (0.5 – 9 ppm).

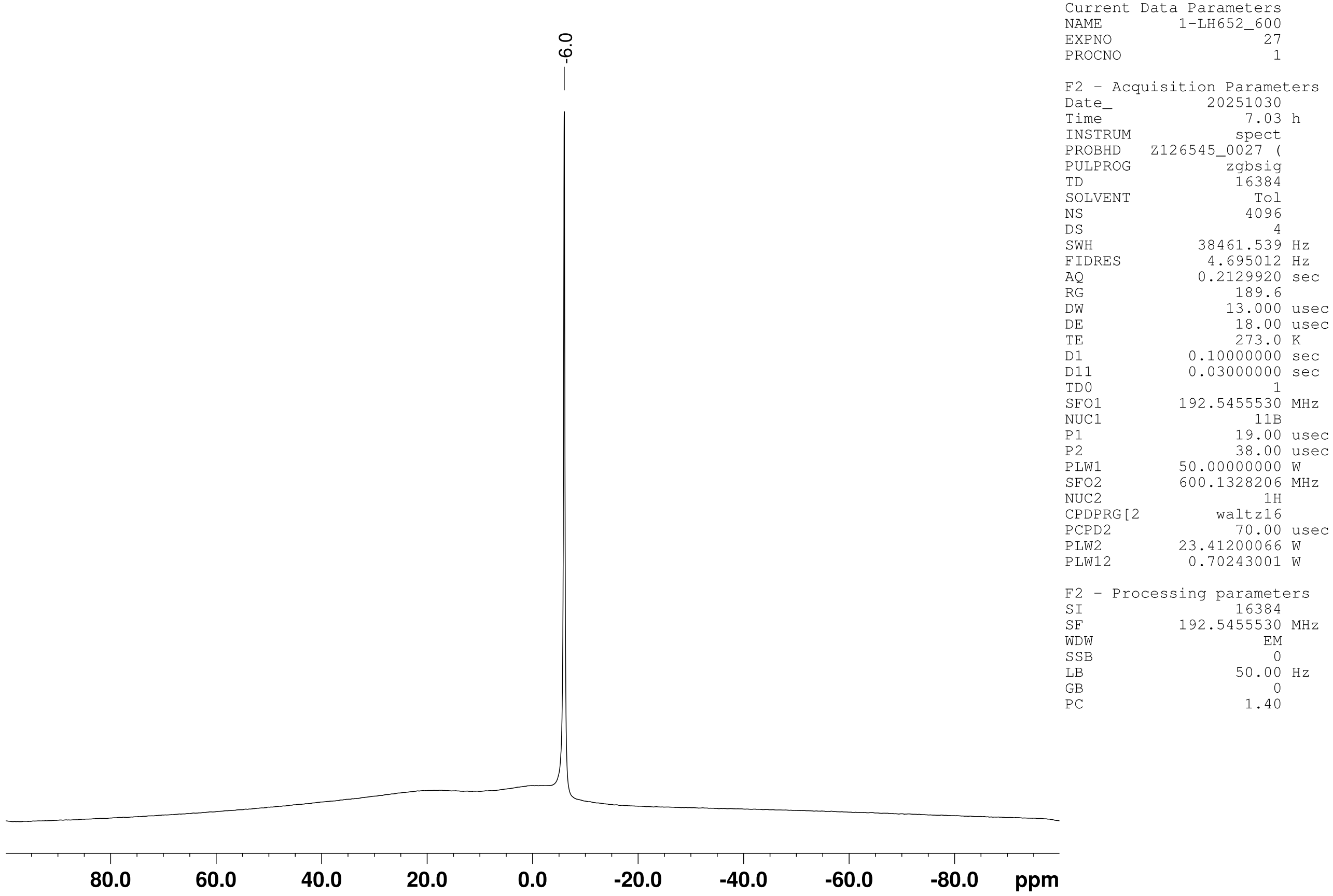

Figure SI17. $^{11}B\{^1H\}$ NMR of compound **3**.

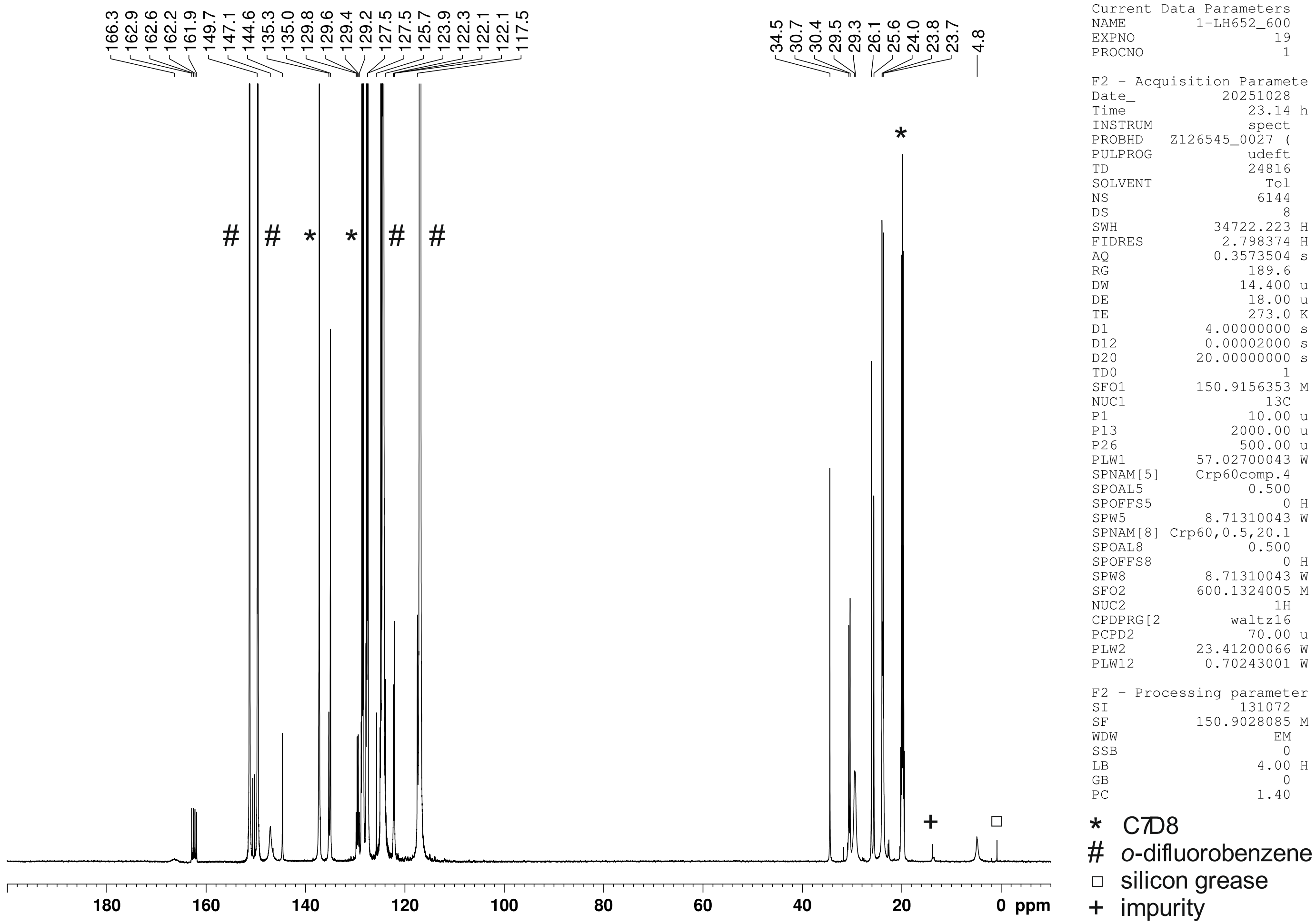

Figure SI18. $^{13}C\{^{1}H\}$ NMR of compound **3**.

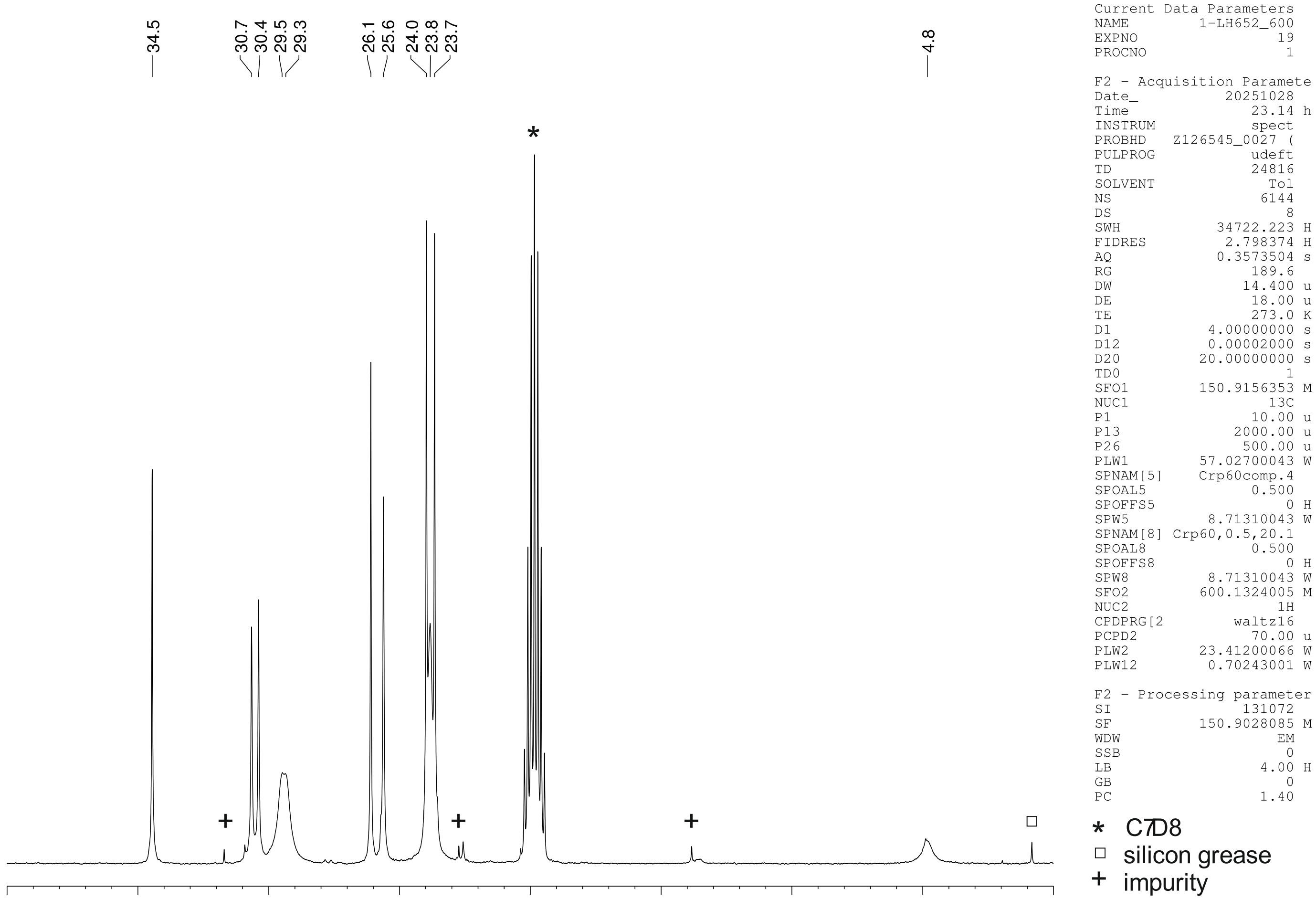

Figure SI19. $^{13}C\{^1H\}$ NMR of compound **3** (0 – 40 ppm).

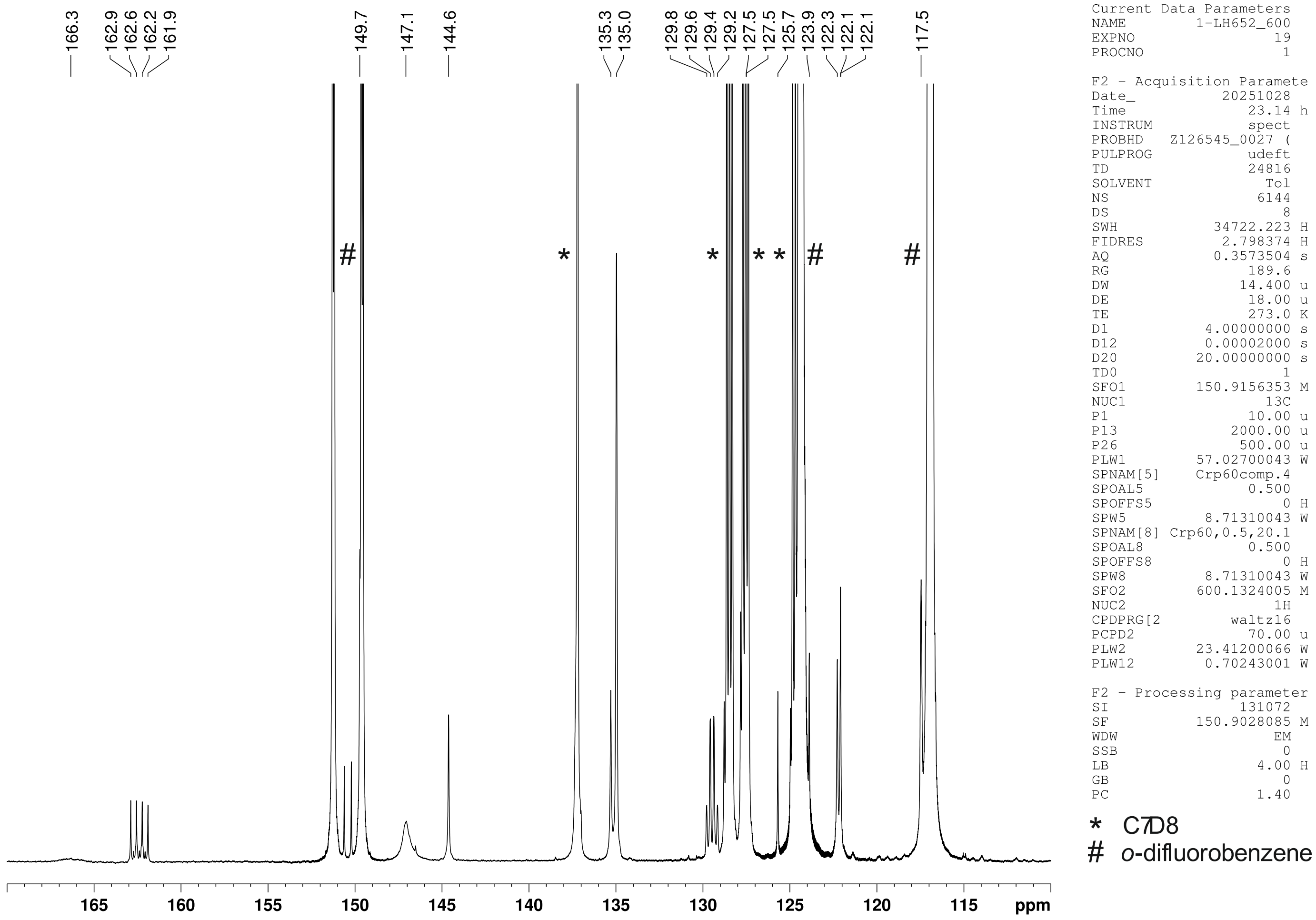

Figure SI20. $^{13}C\{^{1}H\}$ NMR of compound **3** (110 – 170 ppm).

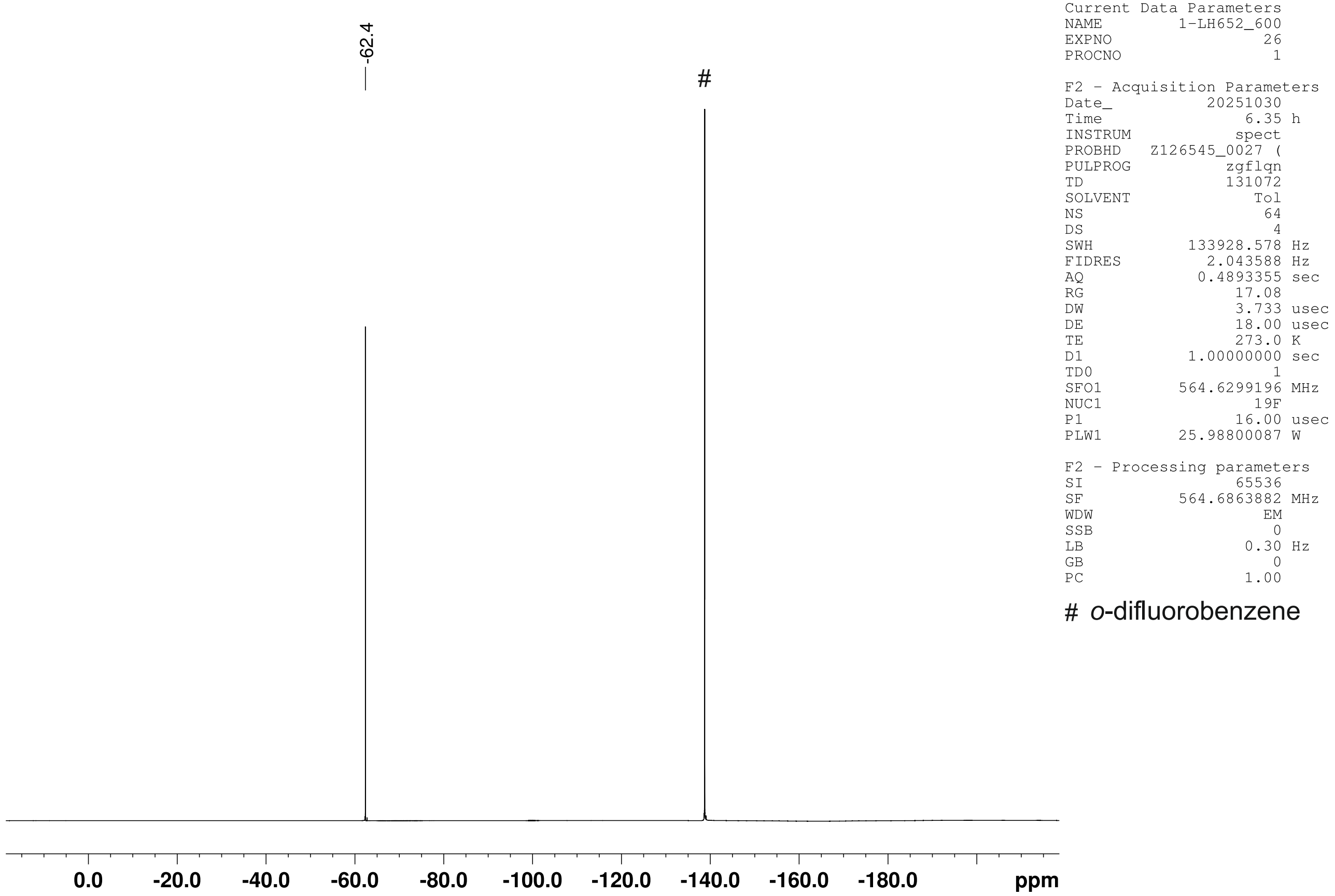

o-difluorobenzene

Figure SI21. $^{19}F\{^{1}H\}$ NMR of compound **3**.

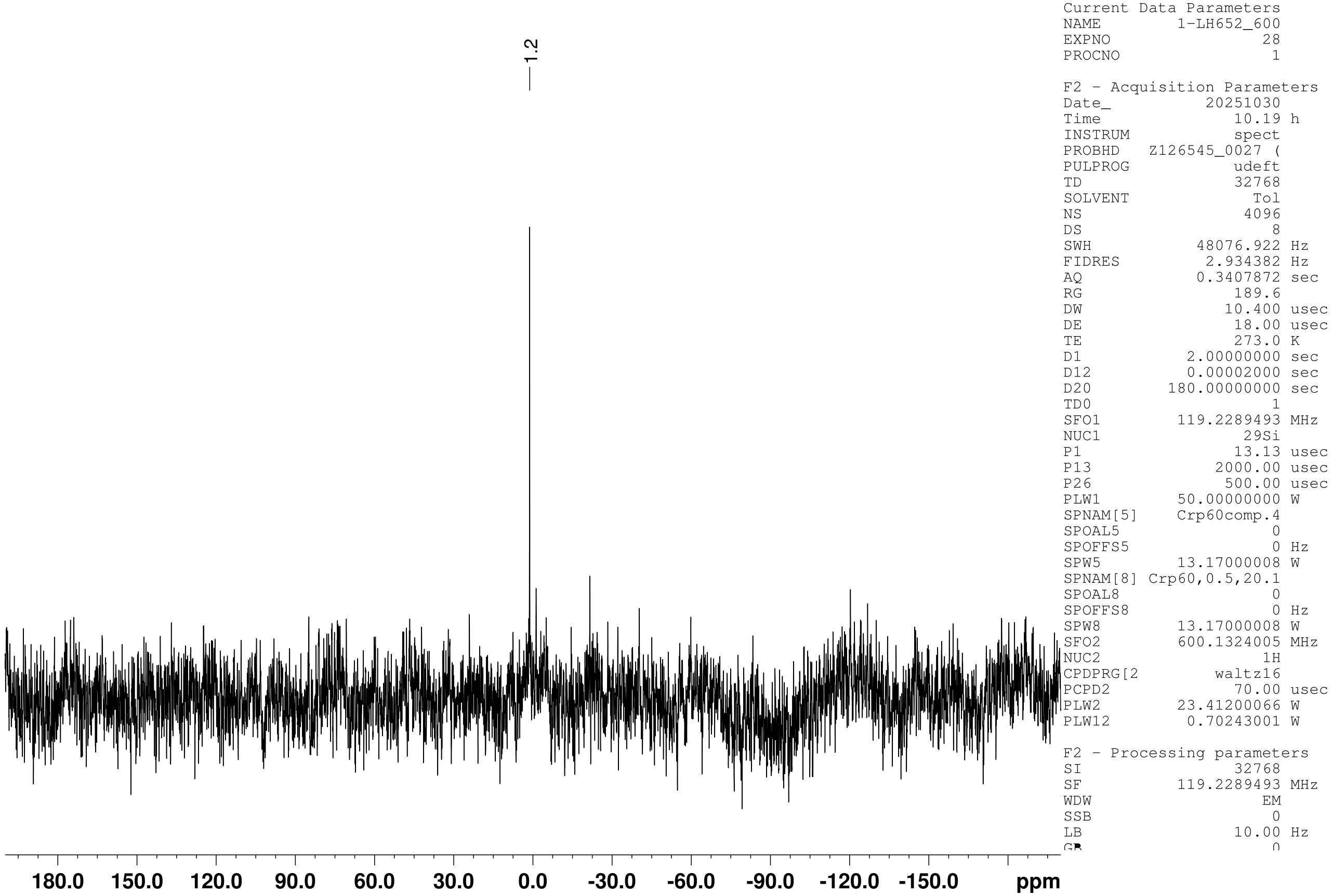

Figure SI22. $^{29}Si\{^{1}H\}$ NMR of compound **3**.

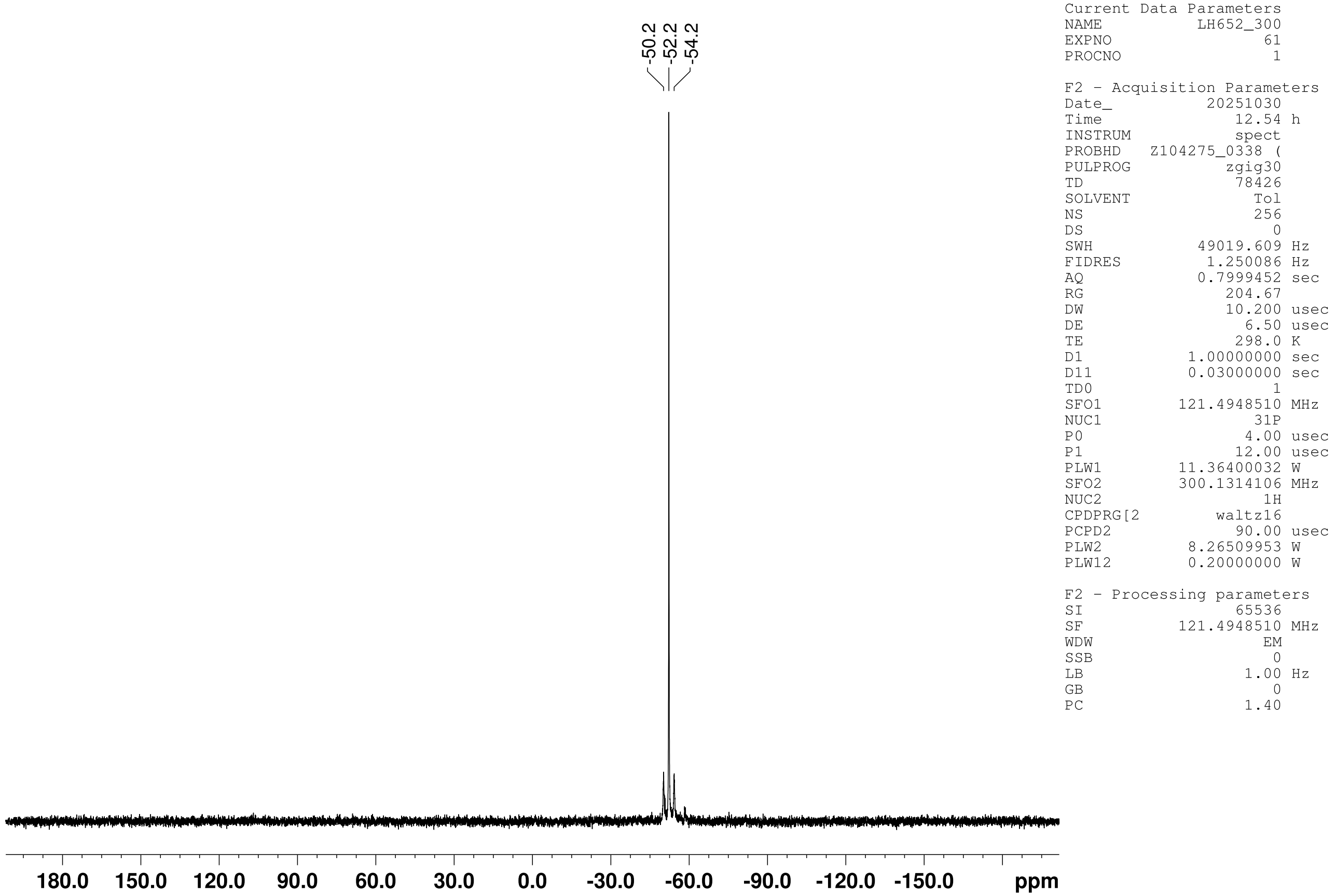

Figure SI23. $^{31}P\{^{1}H\}$ NMR of compound **3**.

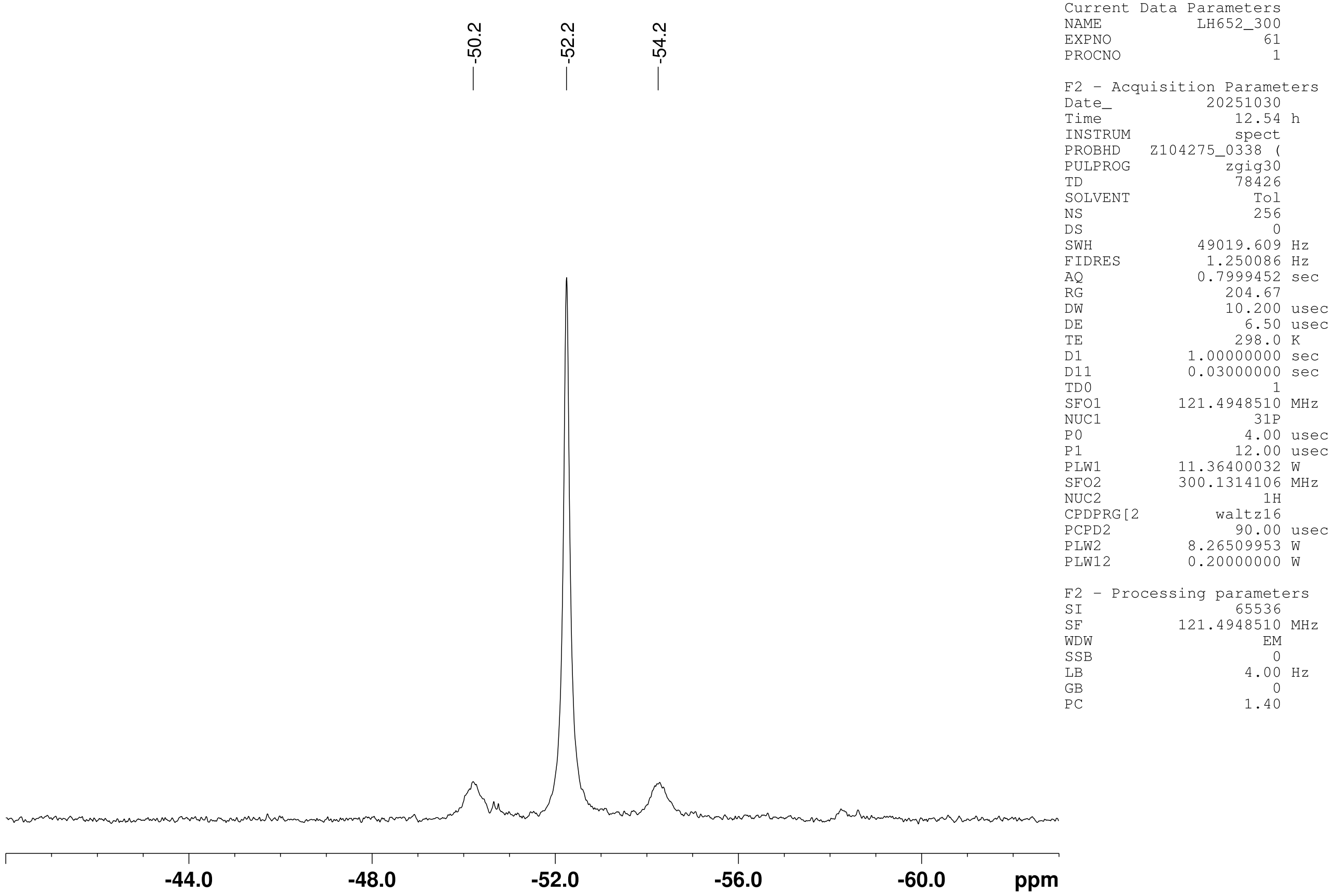

Figure SI24. $^{31}P\{^{1}H\}$ NMR of compound **3** (−44 to −60 ppm).

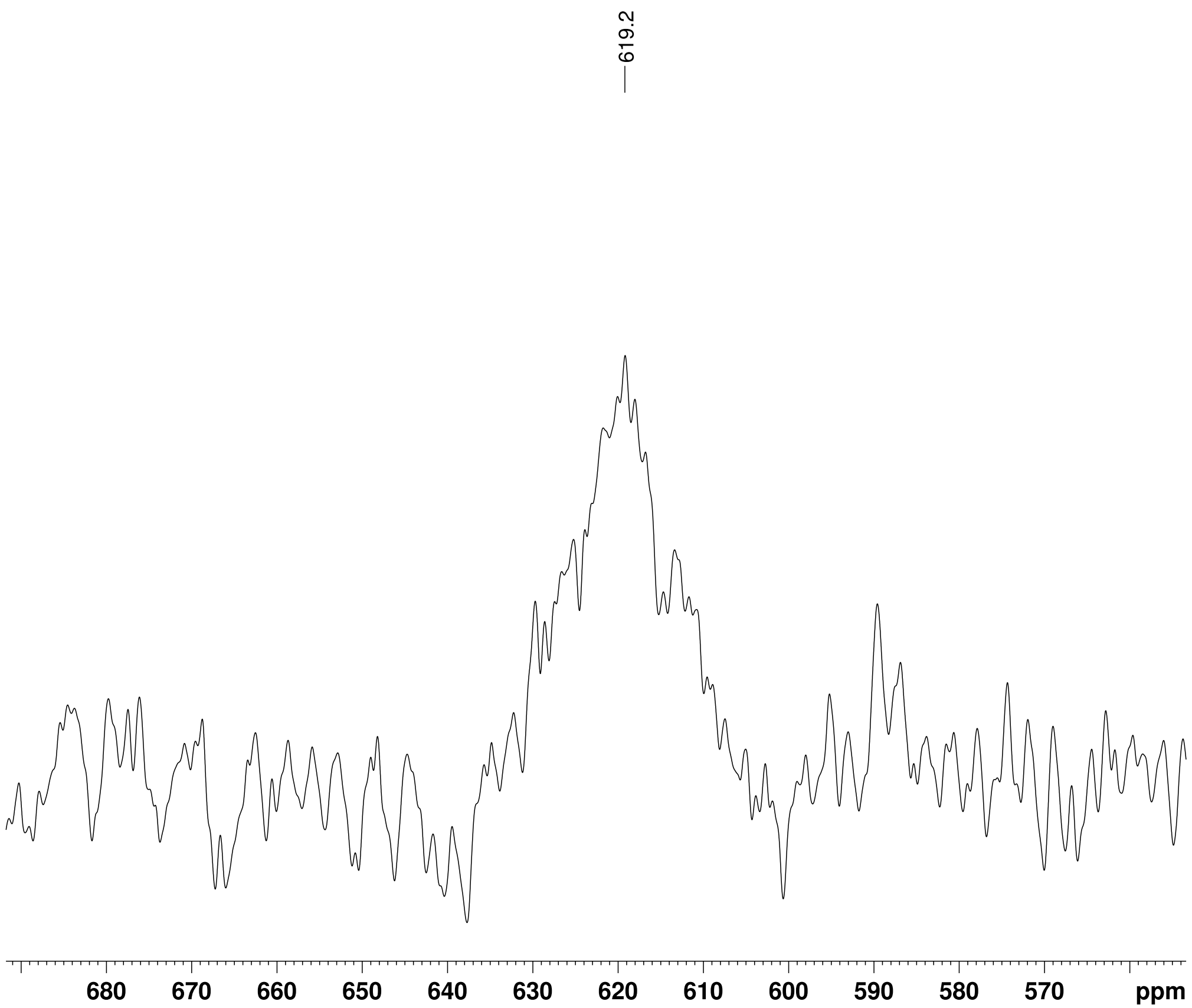


```
Current Data Parameters
NAME            LH572_300
EXPNO                  23
PROCNO                  1

F2 - Acquisition Parameters
Date_           20250523
Time                7.02 h
INSTRUM            spect
PROBHD   Z104275_0338 (
PULPROG           zgig30
TD                 39186
SOLVENT             C6D6
NS                 60000
DS                     4
SWH            89285.711 Hz
FIDRES          4.557021 Hz
AQ             0.2194416 sec
RG                204.67
DW                 5.600 usec
DE                  6.50 usec
TE                 298.0 K
D1            0.10000000 sec
D11           0.03000000 sec
TD0                    1
SFO1         112.0266978 MHz
NUC1               119Sn
P0                  4.03 usec
P1                 12.10 usec
PLW1         12.00000000 W
SFO2         300.1312005 MHz
NUC2                  1H
CPDPRG[2         waltz16
PCPD2              90.00 usec
PLW2          8.26509953 W
PLW12         0.20000000 W

F2 - Processing parameters
SI                 65536
SF           111.9203738 MHz
WDW                   EM
SSB                    0
LB                100.00 Hz
GB                     0
PC                  1.40
```

Figure SI25. $^{119}Sn\{^{1}H\}$ NMR of compound **3**.

UV-Vis spectroscopy

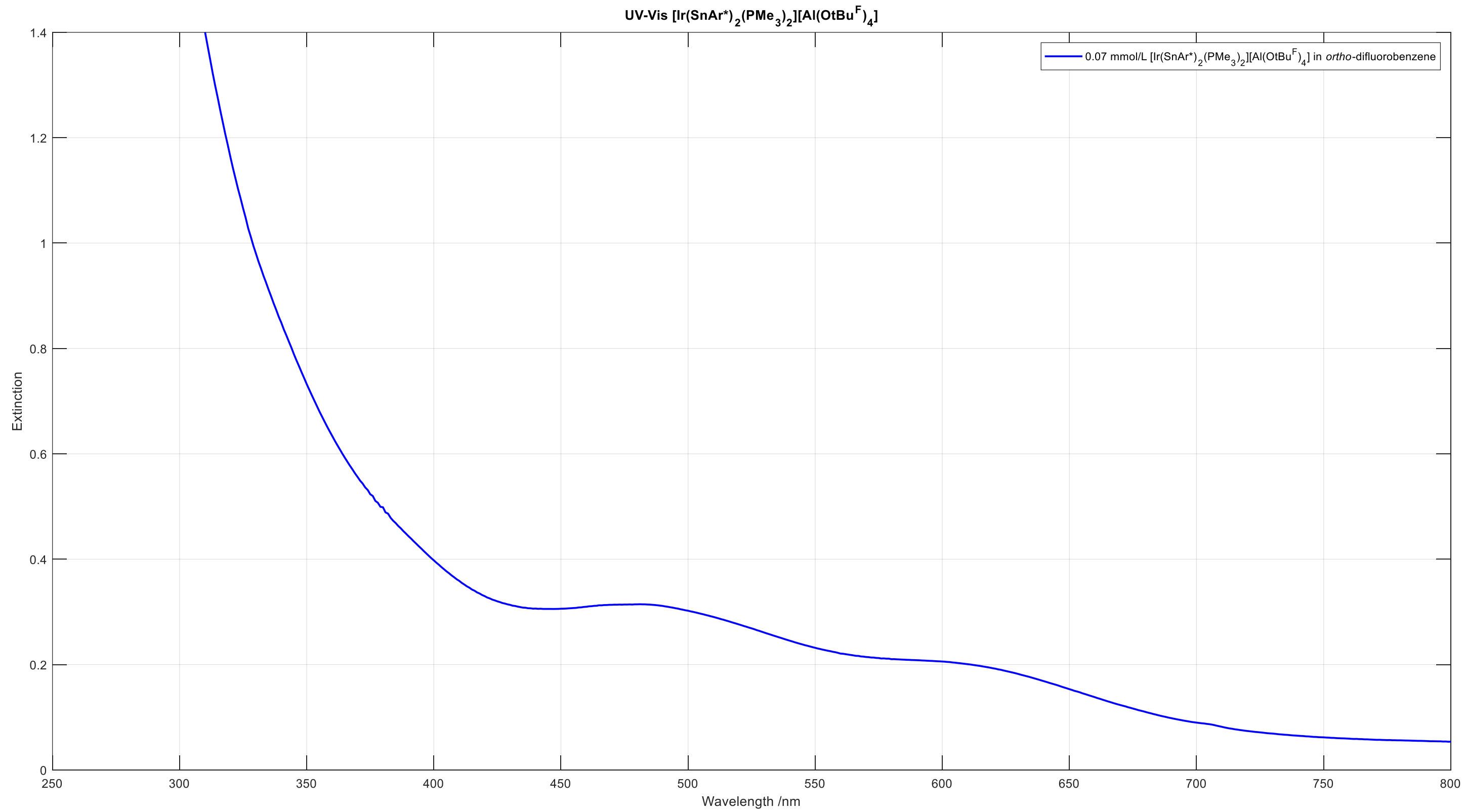

Figure SI26. UV-Vis spectrum of **1a**.

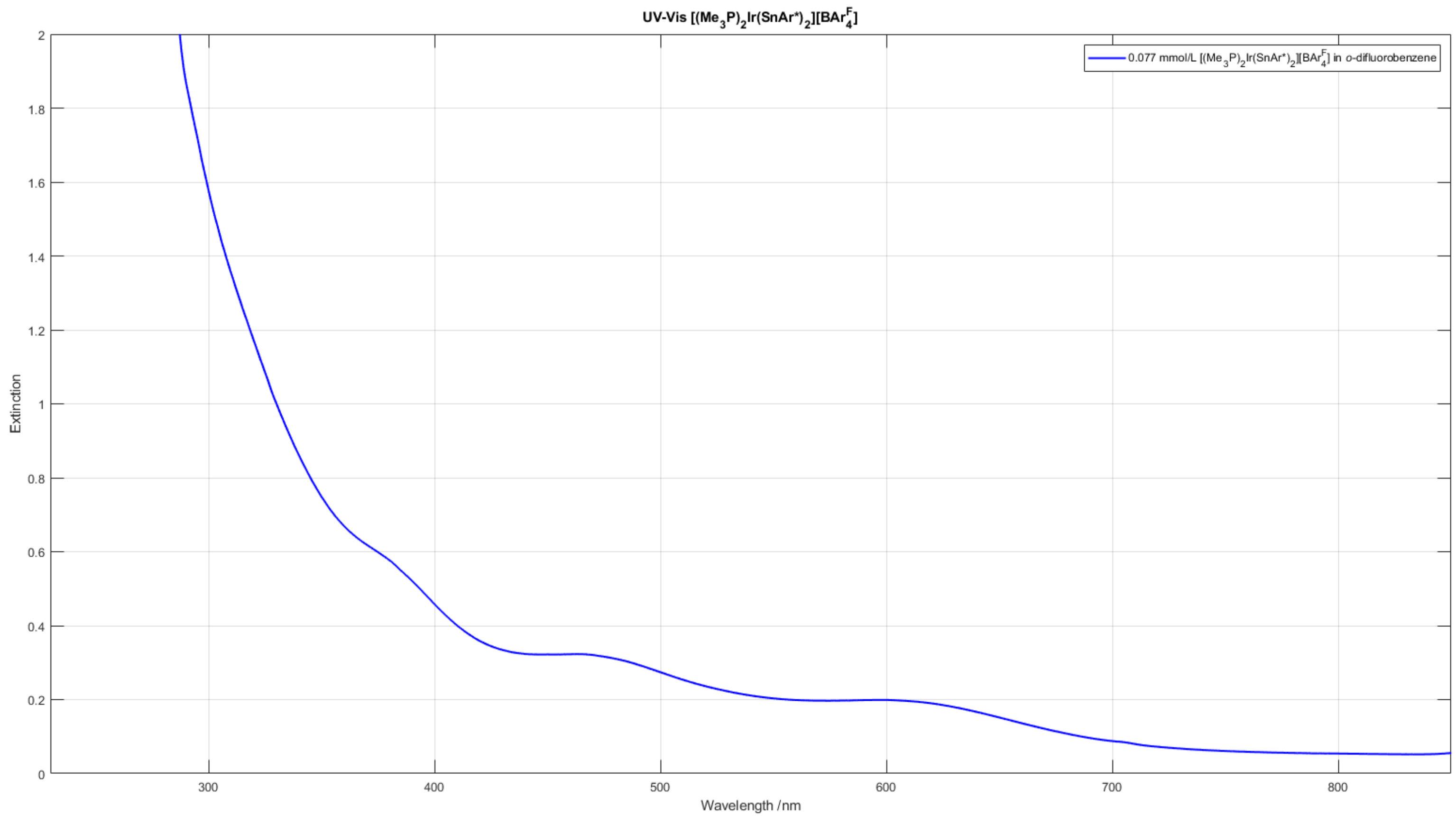

Figure SI27. UV-Vis spectrum of **1b**.

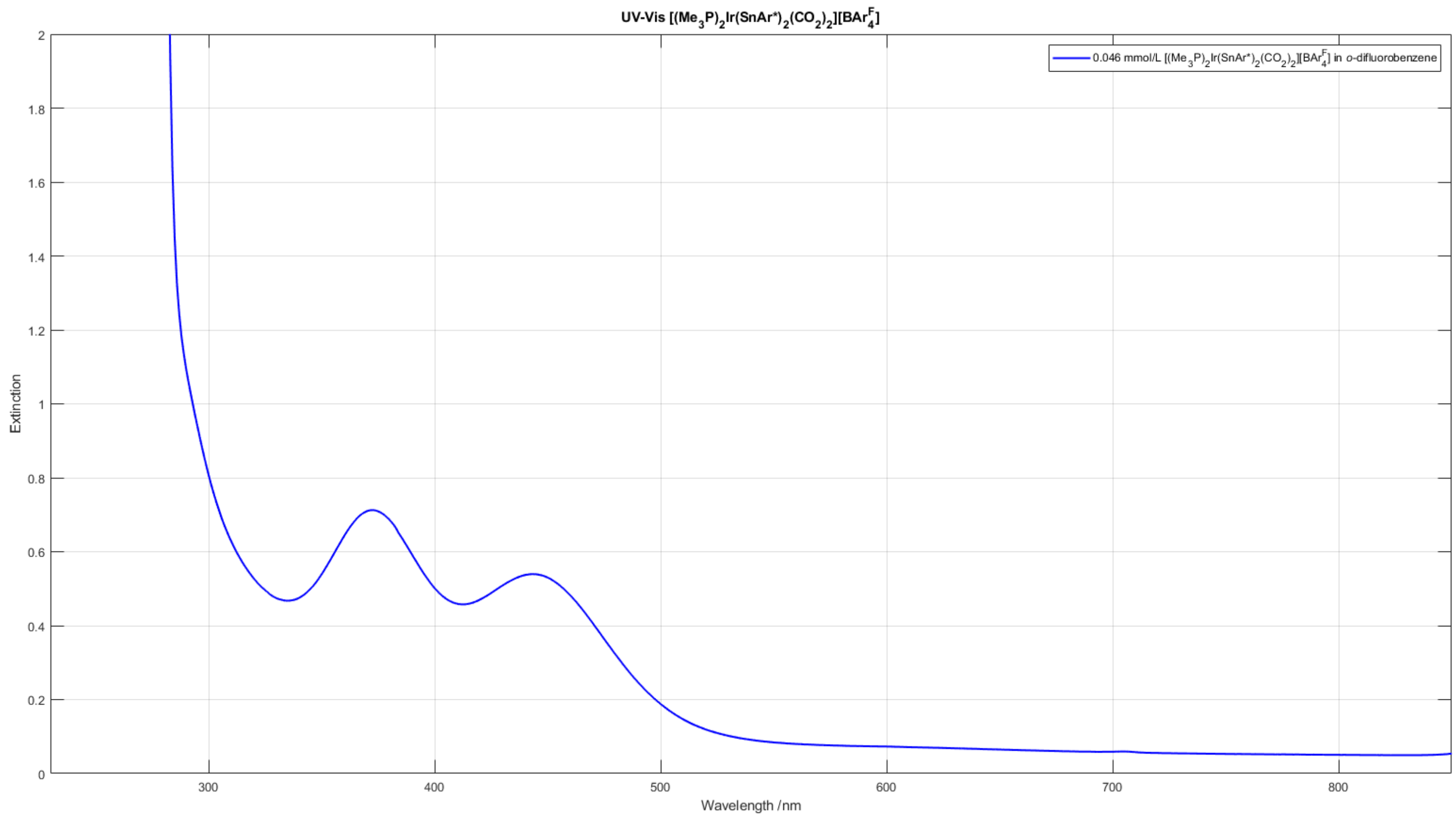

Figure SI28. UV-Vis spectrum of **2**.

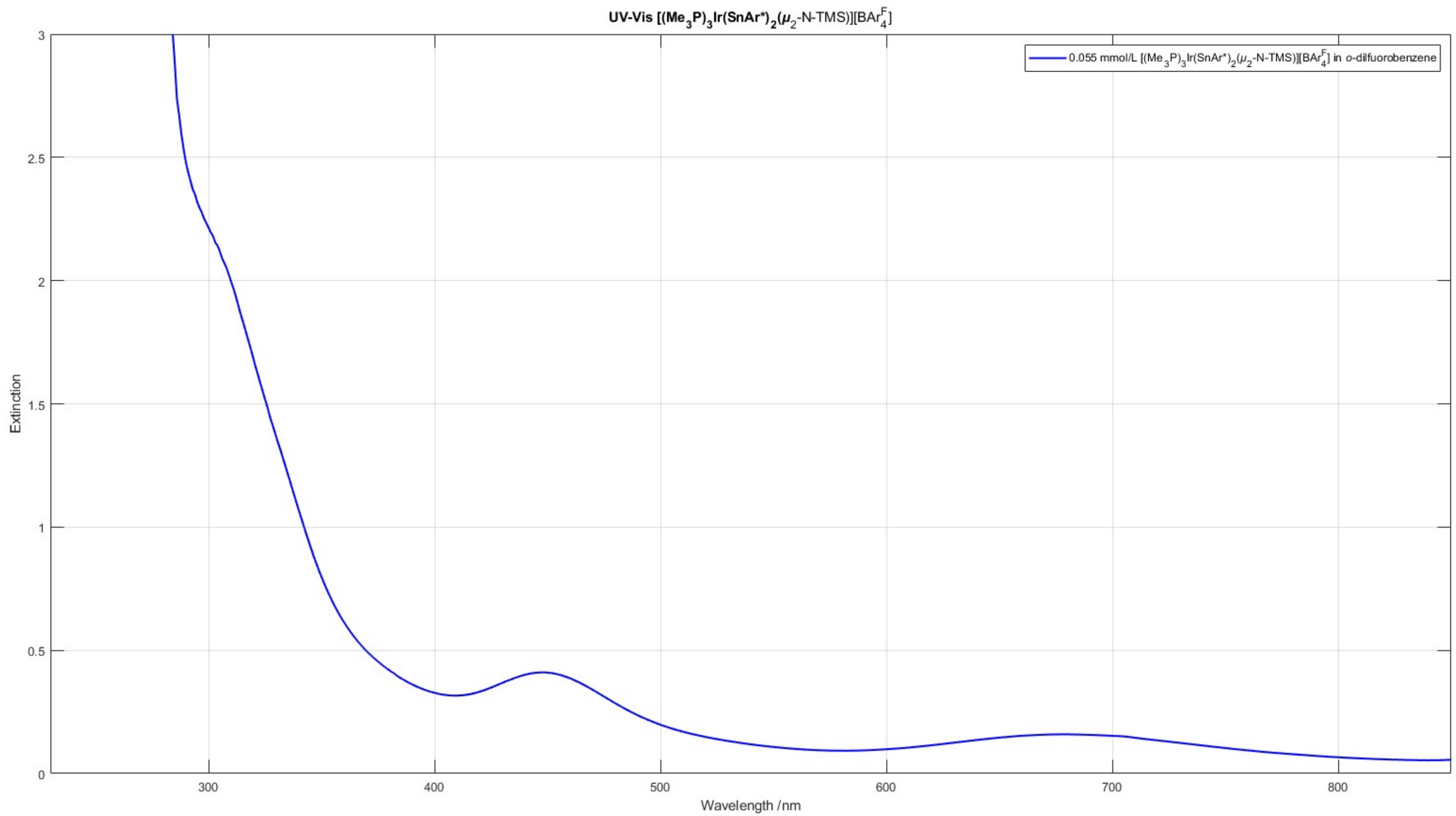

Figure SI29. UV-Vis spectrum of **3**.

IR-Spectroscopy

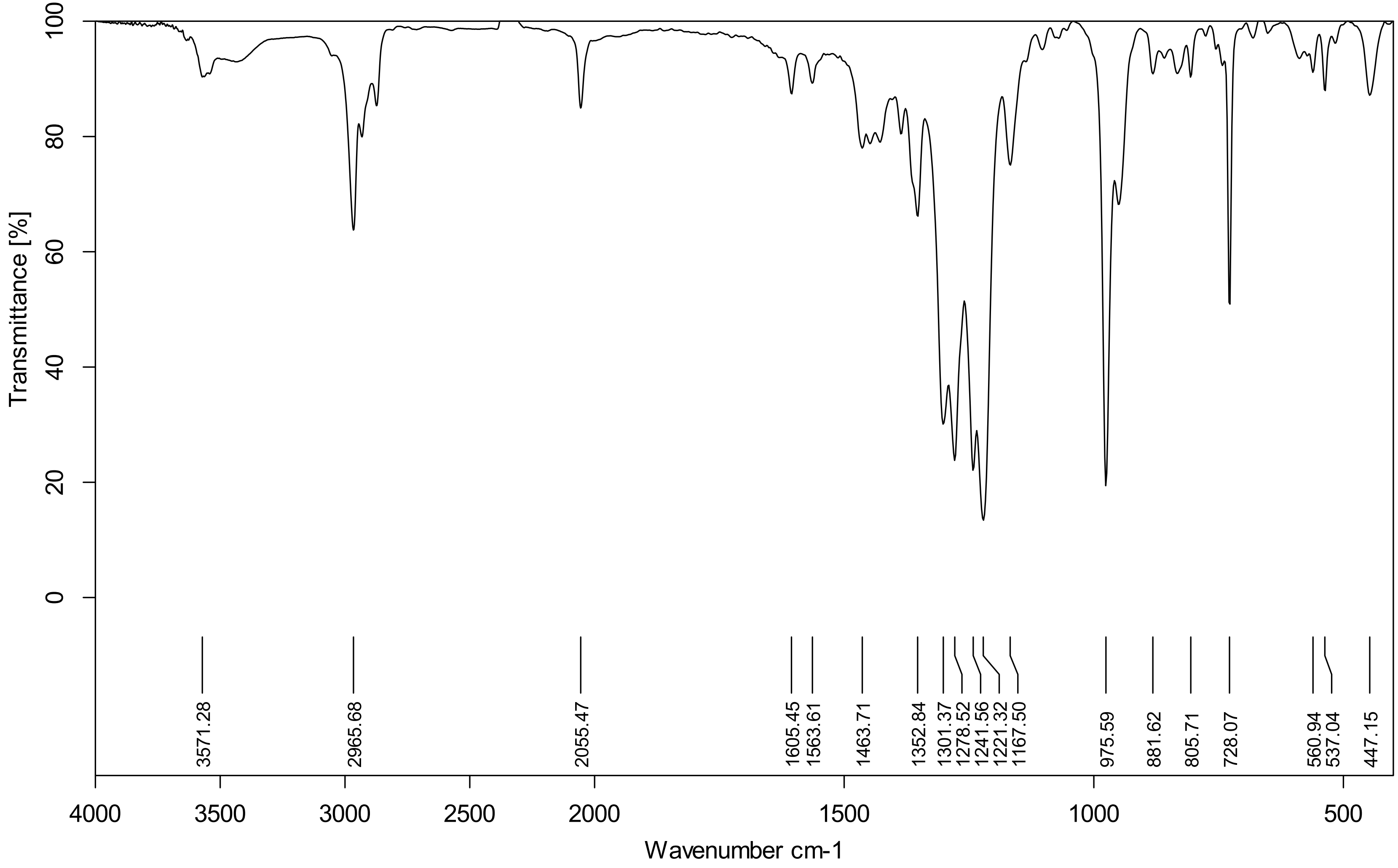

Figure SI30. IR-spectrum of **2**.

Quantum chemistry

On the basis of the molecular structures of the cation of **1**, compound **2** and **3** determined in the solid state, the structure of the complex was optimized using the programme Orca5.0.3[17-19] along with Grimme´s dispersion correction and Becke-Johnson damping (D3BJ) and BP86 with suitable RI approximations.[20, 21] The basis sets employed were def2-TZVP for Ir, Sn, P as implemented in ORCA5.0.3, and def2-SVP on all other elements.[22-25] For all calculations, tight or very tight convergence criteria were applied for optimisations and SCF convergence, respectively.[26-28] A small imaginary frequency was obtained for **3** (–11 cm^{-1}). Analyses of the electronic structure was performed using NBO7and IBO.[29-31]

Compound **1**

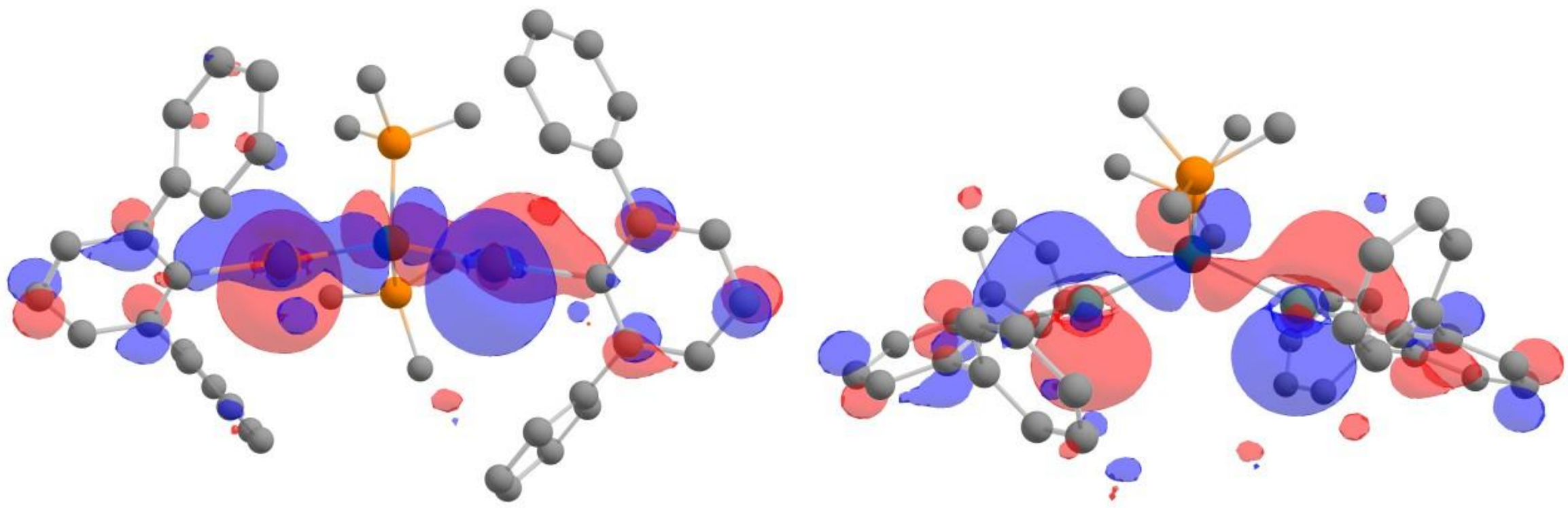

Figure SI31. LUMO of **1** left front view, right top view (contour value = 0.03).

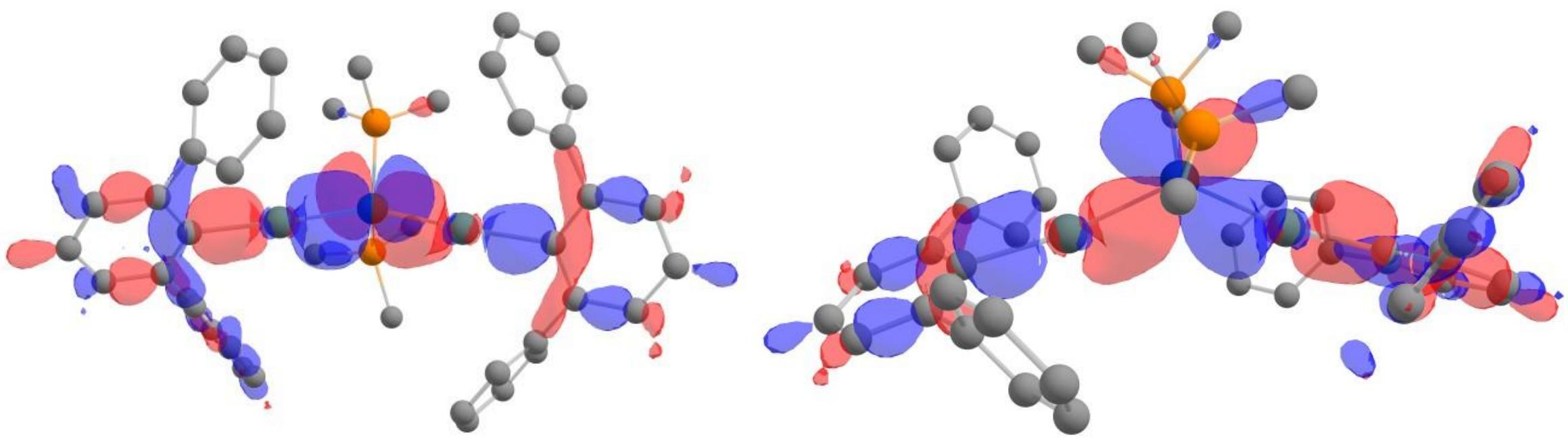

Figure SI32. HOMO-17 of **1** left front view, right top view (contour value = 0.03).

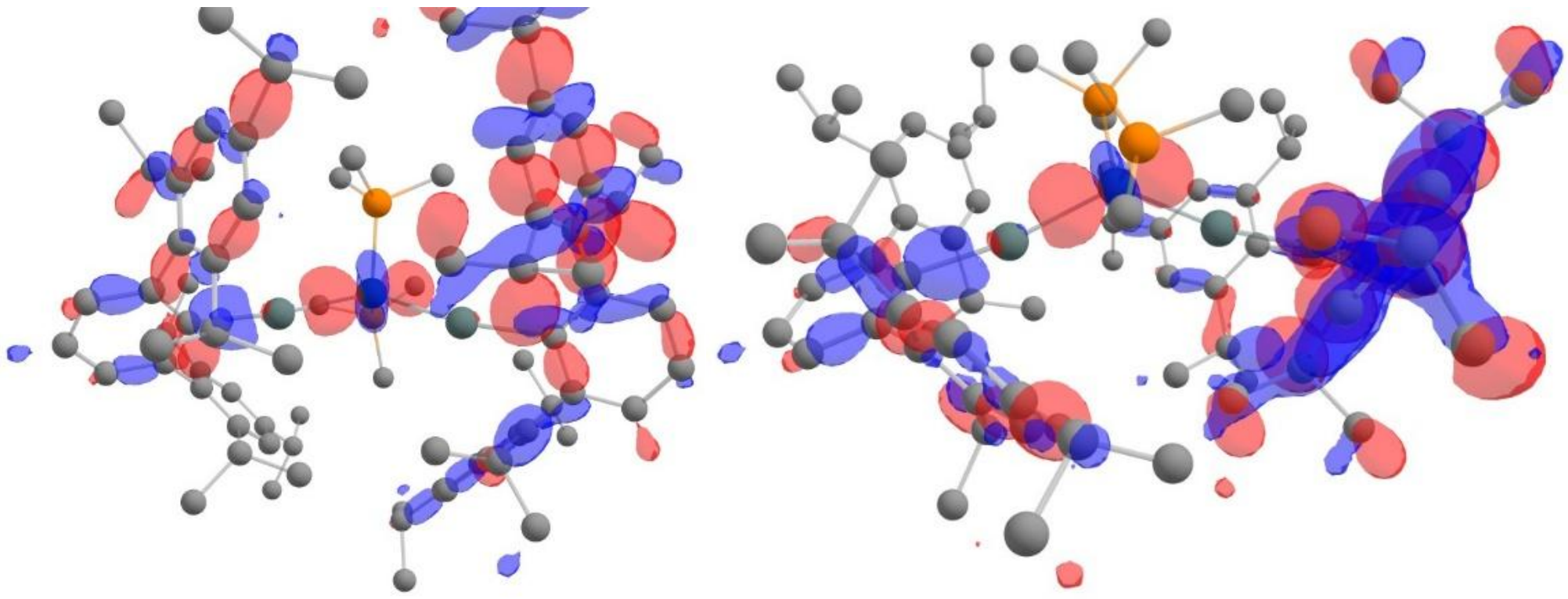

Figure SI33. HOMO-18 of **1** left front view, right top view (contour value = 0.03).

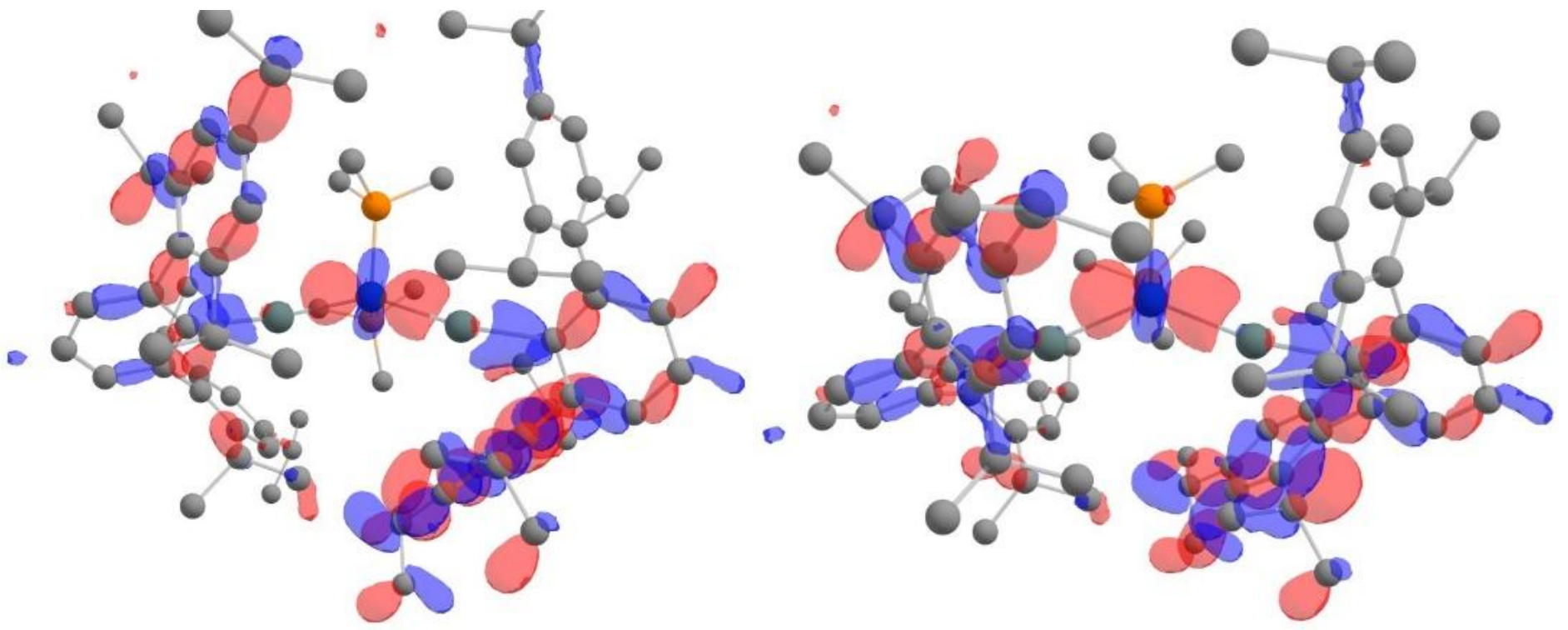

Figure SI34. HOMO-20 of **1** left front view, right top view (contour value = 0.03).

Compound **2**

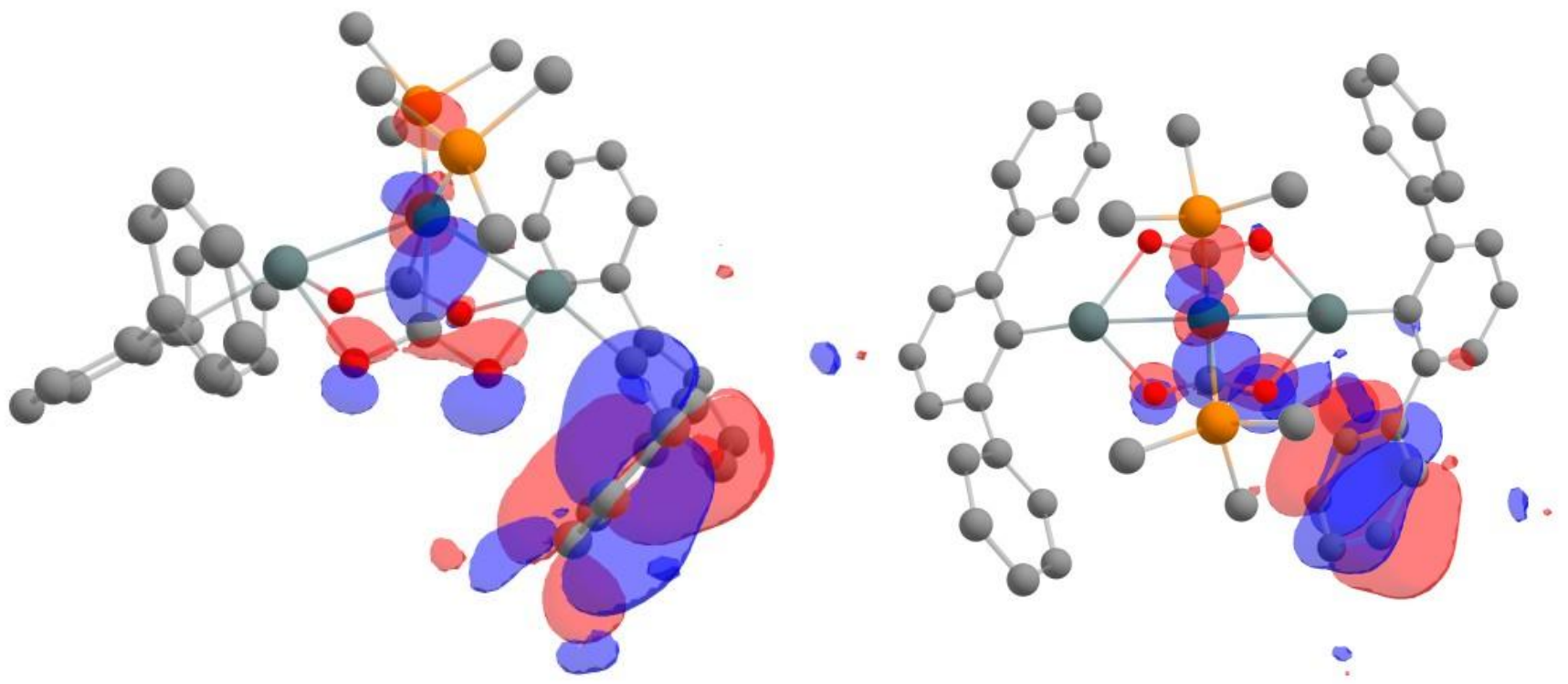

a) HOMO left front view, right top view

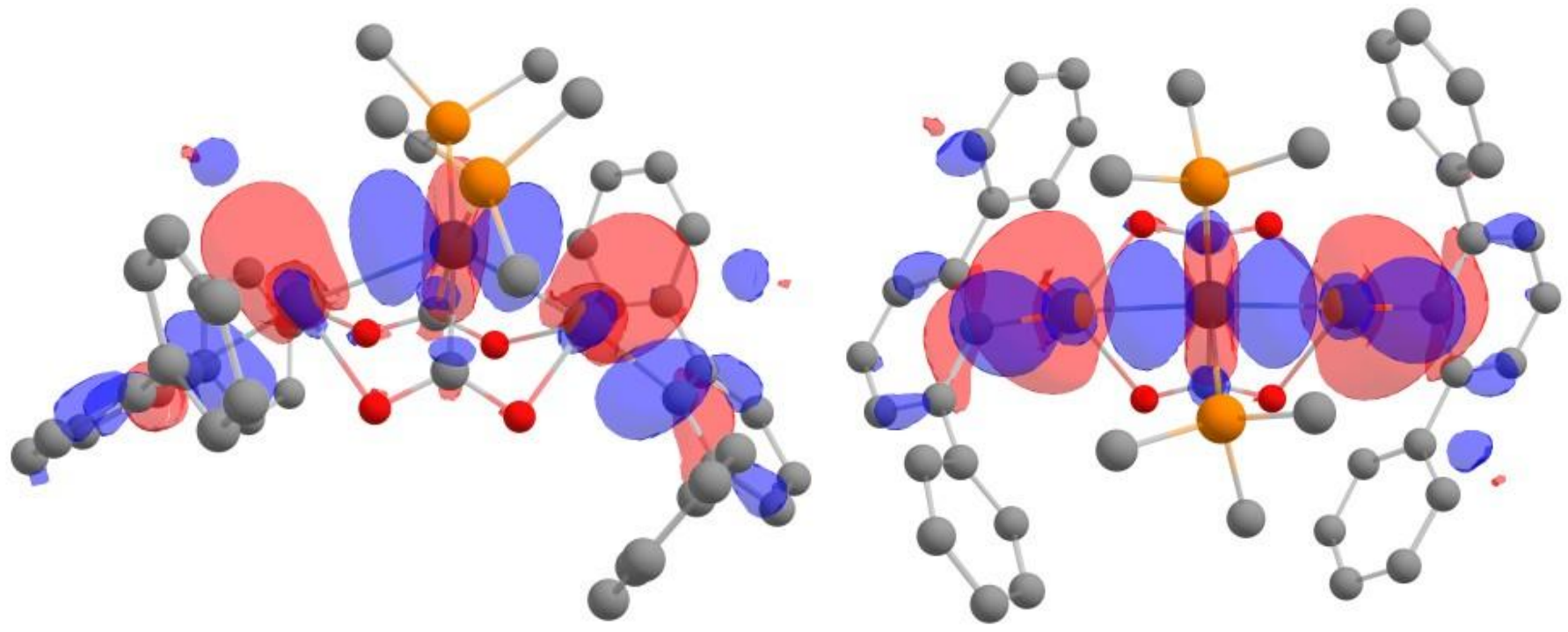

b) LUMO left front view, right top view

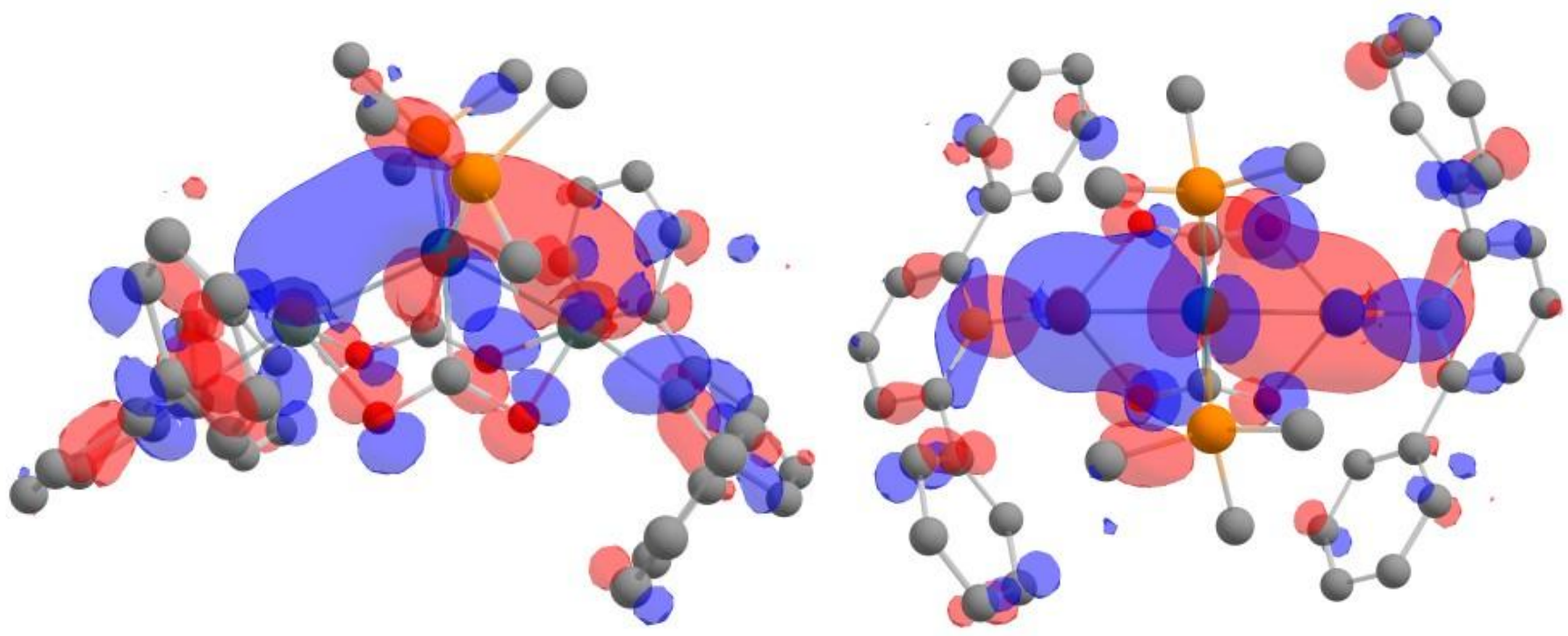

c) HOMO -4 left front view, right top view

Figure SI35. HOMO, LUMO and HOMO-4 (Ir–Sn 3c2e-σ bond) of **2** (contour value = 0.03).

Compound **3**

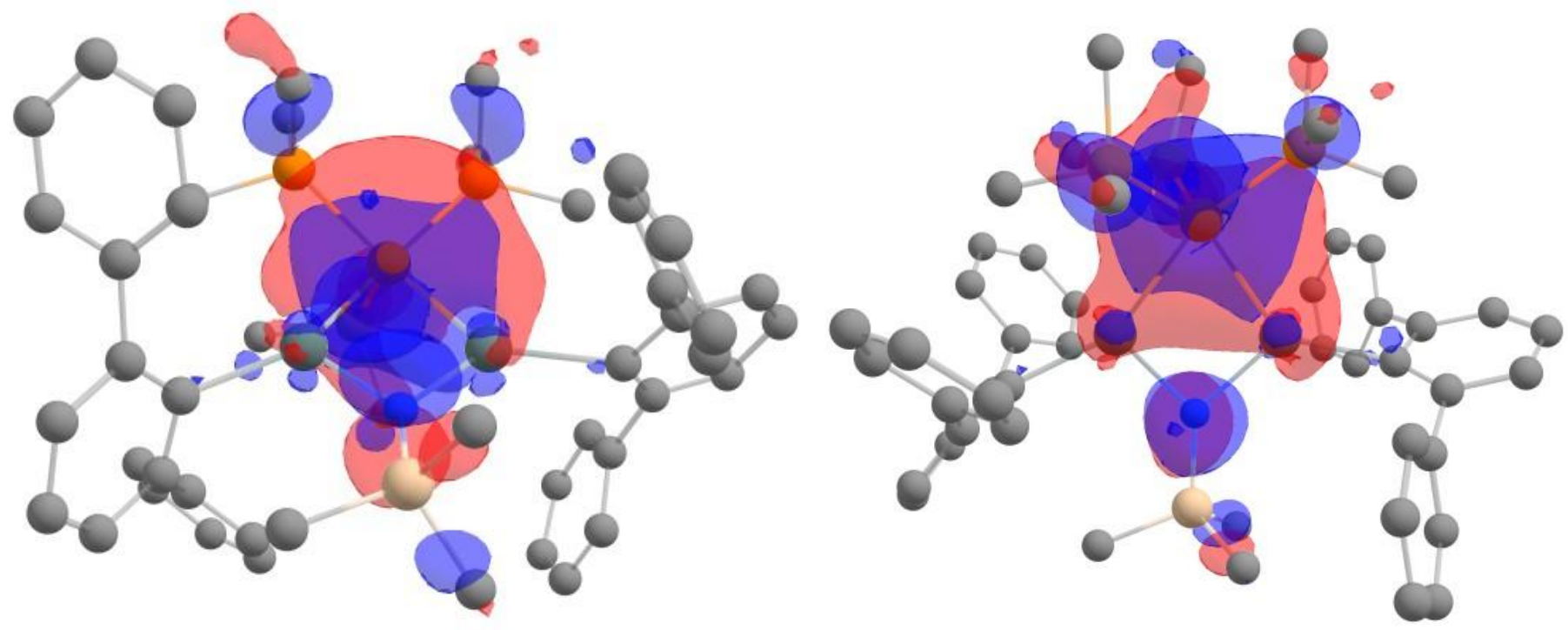

a) HOMO left front view, right top view

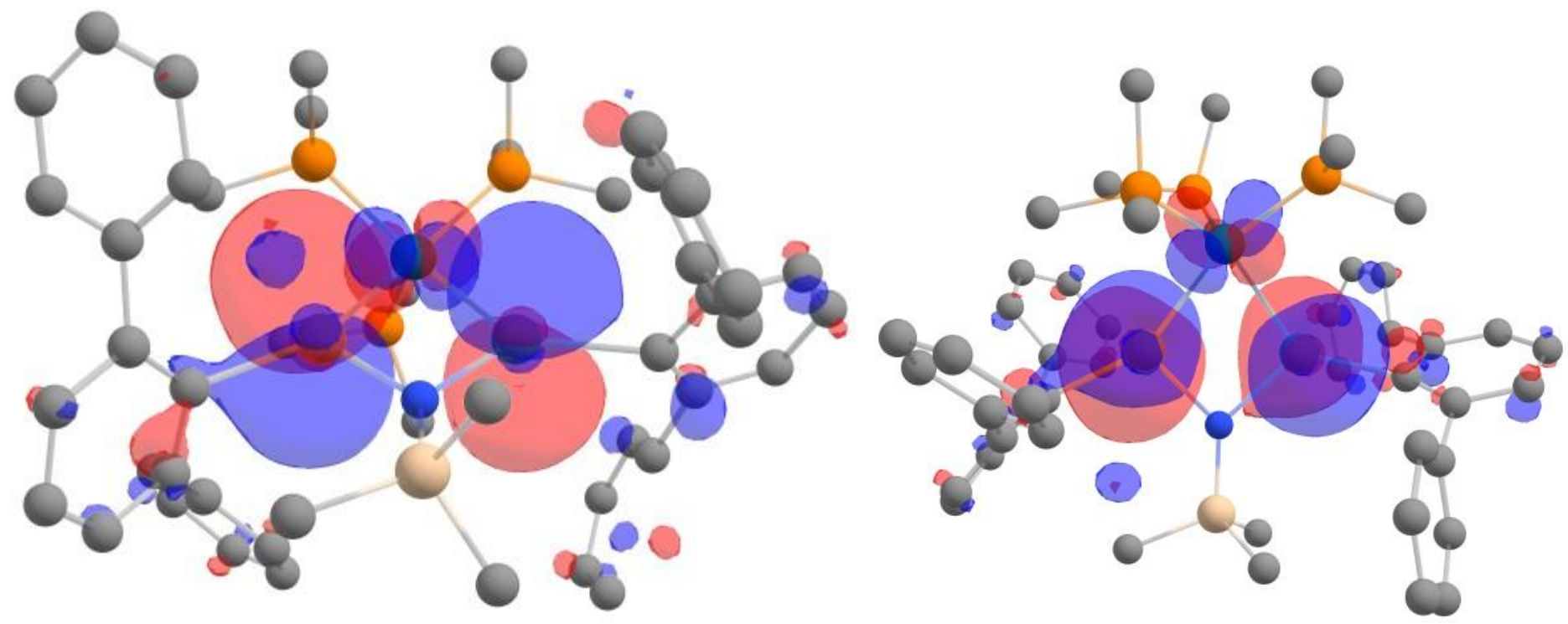

b) LUMO left front view, right top view

Figure SI36. HOMO and LUMO of compound **3** (contour value = 0.03).

IBOs of compound **3**.

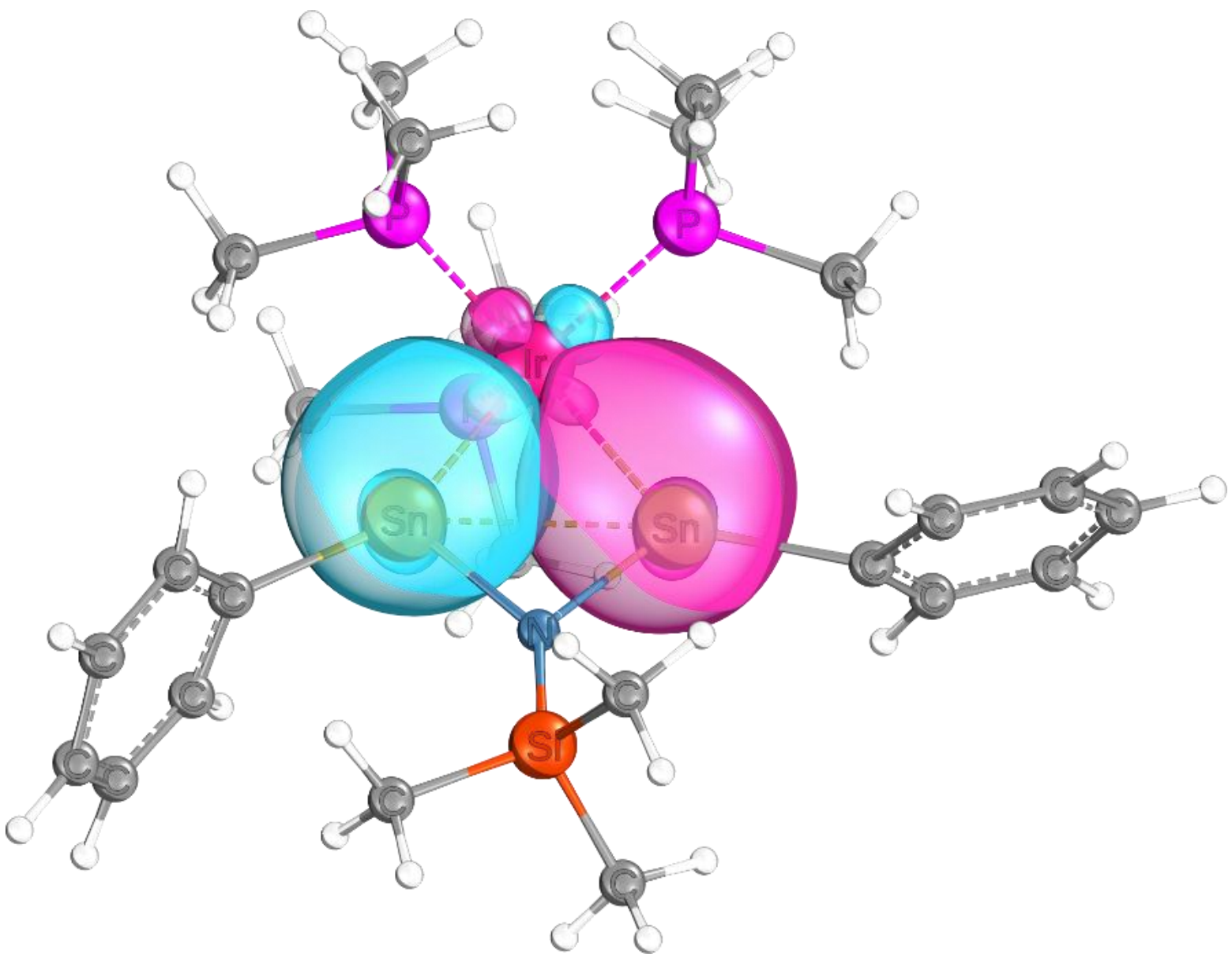

Figure SI37. Combined IBOs 130 and 132, Sn lone pairs as σ donors to Ir (IBO 130 Sn left 76%, Ir 16%; IBO 132 Sn right 79%, Ir 13%).

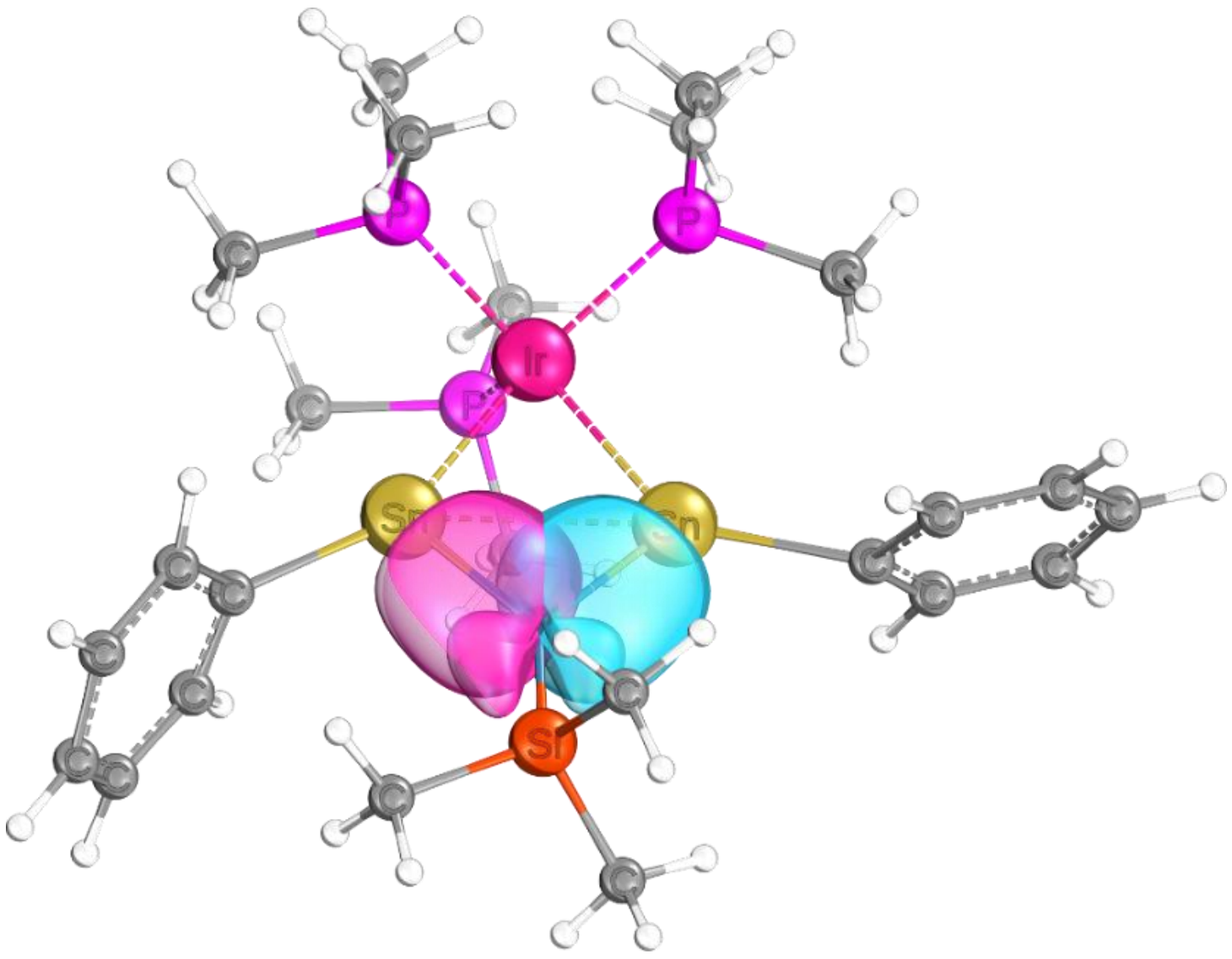

Figure SI38. Combined IBOs 129 and 131, N–Sn σ bonds (each IBO N 70%, Sn 26%).

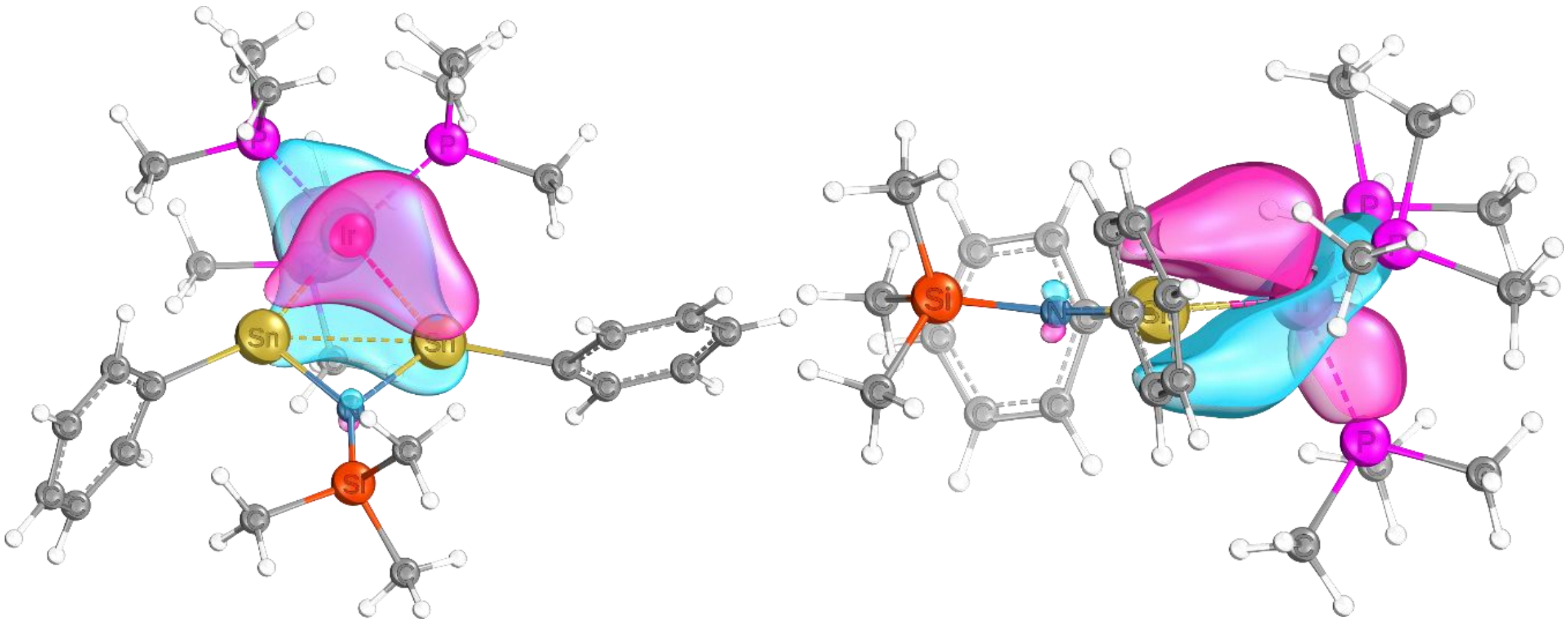

Figure SI39. IBO 155 (two perspectives), Sn–Ir–Sn 3c2e π bond (Ir 75%, Sn left 14%, Sn right 7%).

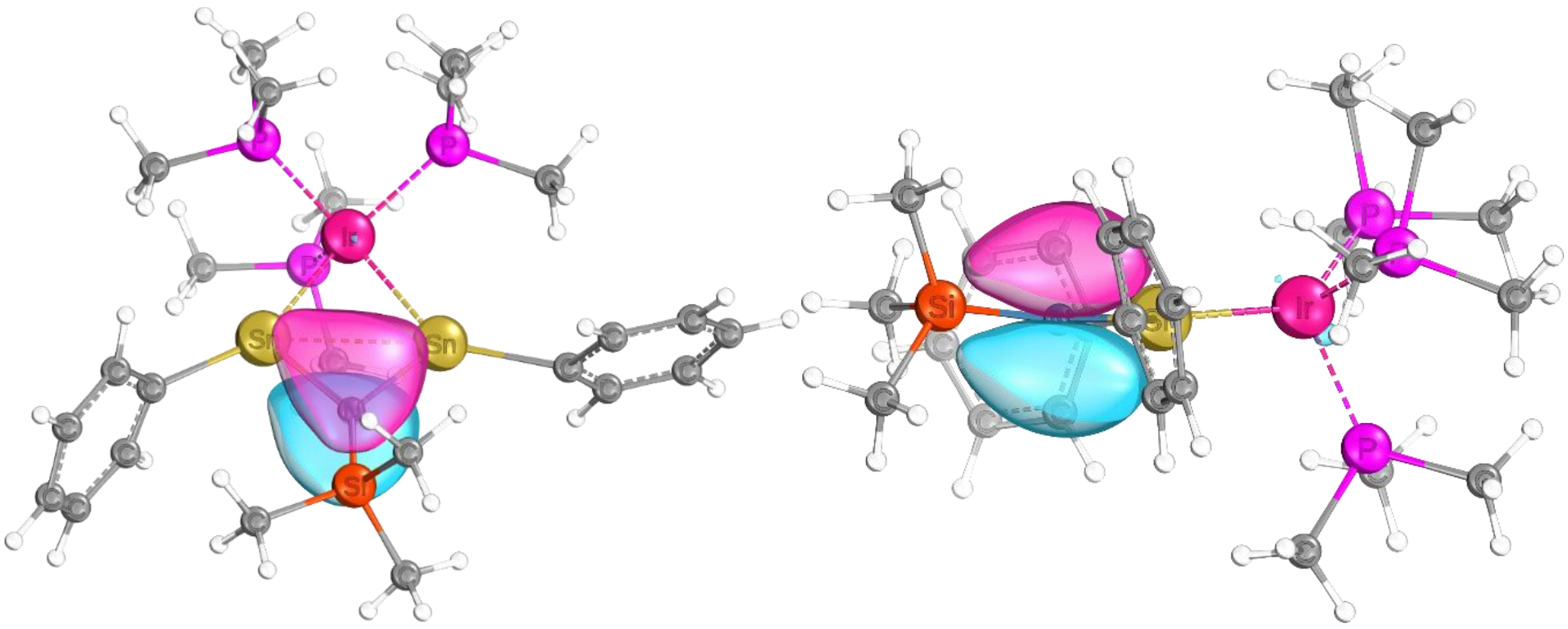

Figure SI40. IBO 158 (two perspectives), N lone pair (N 79%, Sn left 9%, Sn right 6%, Si 4%)

Table SI2. NLMOs of compound **3** with atomic and orbital contributions (see also Figure 7) (a-e belongs to Figure 7 in the manuscript).

NLMO	138 (a)	137 (b)	142(c)	136 (d)	131 (e)
Occ (e^{-})	1.6922	1.7892	1.9633	1.824	1.6923
Ir%	2 (6s 13%, 5d 87%)	1 (6s 12%, 5d 87%)			85 (5d 100%)
Sn1%	6 (5s 72%, 5p 26%)	3 (5p 96%)	1 (5p 95%)	3 (5s 18%, 5p 80%)	6 (5p, 99%)
Sn2%	3 (5s 33%, 5p 61%)	4 (5p 97%)	1 (5p 95%)	3 (5s 21%, 5p 77%)	5 (5p, 99%)
N%	85 (2p 100%)	90 (2s 1%, 2p 99%)	81(2s 62%, 2p 38%)	91 (2s 36%, 2p 64%)	
Si%	1 (3p 100%)	1.5 (3p 100%)	17 (3s 32%, 3p 68%)	0.5 (3s 51%, 3p (44%)	

ETS-NOCV analysis

Analysis of [(RSn)$_2$Ir(PMe$_3$)$_2$]$^+$: ETS-NOCV analysis[32] was performed using ADF2022 programme suite with BP86-D3BJ[20, 26, 27]/TZ2P[33] model chemistry taking relativistic effect into account by using the scalar zeroth order regular approximation (ZORA) method. Input structure for the ETS-NOCV analysis was based on the experimental structure obtained from X-ray diffraction for the full molecule [(Ar*Sn)$_2$Ir(PMe$_3$)$_2$]$^+$ for which the bulky Ar* was stripped down to Me or Ph followed by a restrained geometry optimization with coordinates of the inorganic core (Ir,Sn and P-atoms) fixed to the experimental values and only C and H atoms allowed to optimize by DFT (BP86-D3BJ/TZ2P/ZORA(SO)).[34, 35] To probe individual bonding interactions between [RSn]$^+$ fragments and the [Ir(PMe$_3$)$_2$]$^-$ fragment, two species were analysed in and ETS-NOCV analysis resulting from stepwise removal of a [RSn]$^+$ fragment.

[(RSn)$_2$Ir(PMe$_3$)$_2$]$^+$ → [RSn]$^+$ and [(RSn)Ir(PMe$_3$)$_2$] → [RSn]$^+$ and [Ir(PMe$_3$)$_2$]$^-$

ETS-NOCV findings for respectively examined [(PhSn)$_2$Ir(PMe$_3$)$_2$]$^+$ and [(MeSn)$_2$Ir(PMe$_3$)$_2$]$^+$ were qualitatively identical. (R = Me, Ph; findings were qualitatively identical)

Depictions of deformation densities and SFO were prepared using ADF2022. Graphical depictions of the relevant NOCV interactions and contributing fragment orbitals are provided. Different density plots are shown at an iso-density surface-value of 0.003 a.u. (green: positive charge difference; magenta: negative charge difference) and SFO are shown at an iso-density surface value of 0.05 a.u.

Analysis of [(RSn)$_2$Ir(PMe$_3$)$_2$]$^+$: A summary of graphical depictions of selected instructive NOCV for [(MeSn)$_2$Ir(PMe$_3$)$_2$]$^+$ is shown below. It clearly reveals two similarly strong (–47.0 and –41.4 kcal mol^{-1}) π-type interactions from donation of the [(RSn)Ir(PMe$_3$)$_2$] fragment into the perpendicular empty p-orbitals of the stannylidinium fragment along with a respectively weaker, yet clear σ-type interaction arising from donation of the Sn-lonepair into the [(RSn)Ir(PMe$_3$)$_2$] fragment. We would argue that these three interactions allow for a description of a triple bond.

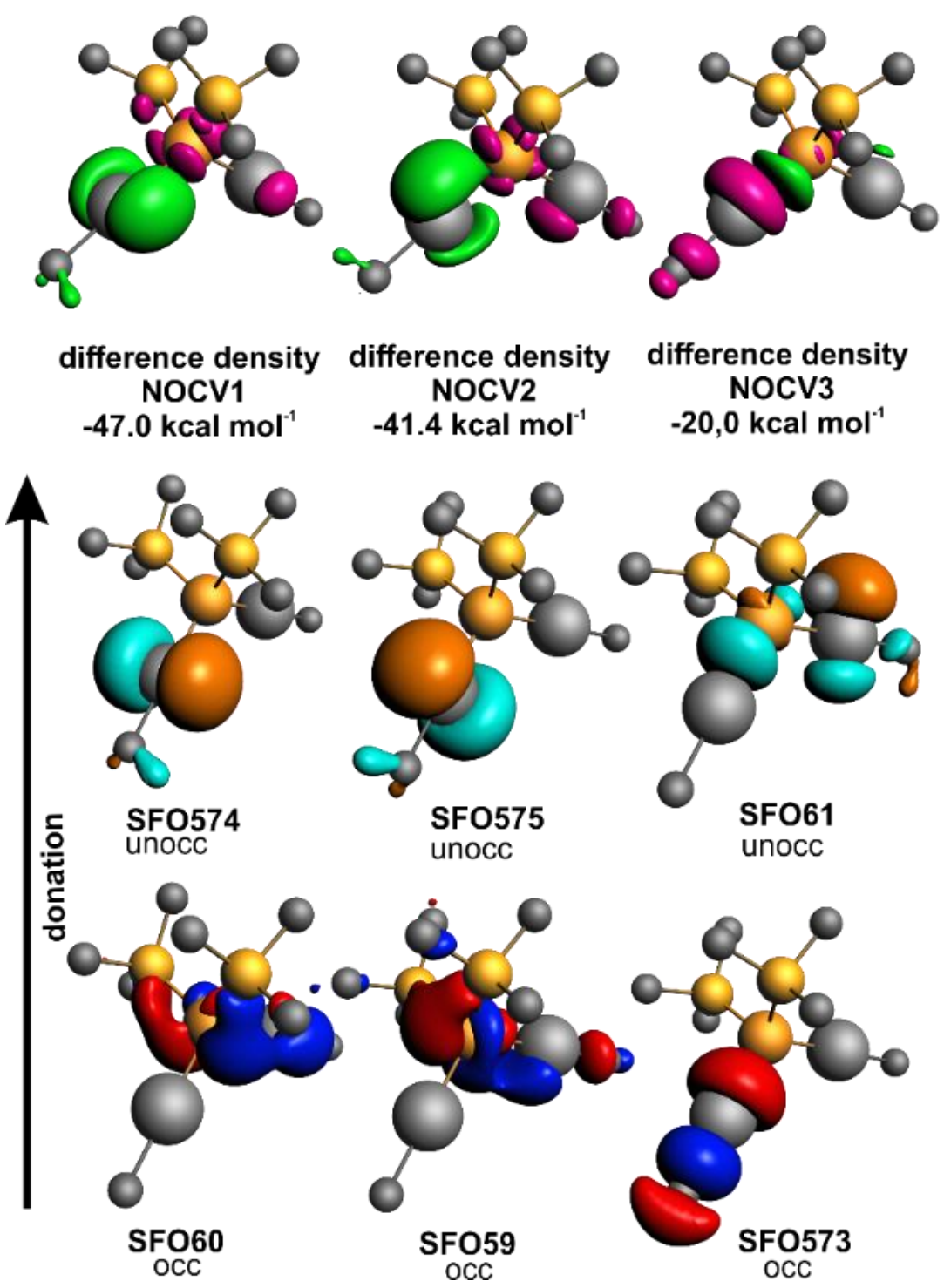

Figure SI41. Relevant NOCV-interactions of [RSn]$^+$ and [(RSn)Ir(PMe$_3$)$_2$].

Analysis of [(RSn)Ir(PMe$_3$)$_2$]: A summary of graphical depictions of selected instructive NOCV for the hypothetical fragment [(MeSn)Ir(PMe$_3$)$_2$] analysing the interaction between a stannylidinium [MeSn]$^+$ and an anionic transition metal fragment [Ir(PMe$_3$)$_2$]$^-$ is shown below. The input structure was generated by deleting one [MeSn]$^+$ fragment from the optimized and accordingly analyzed (*vide supra*) structure of [(MeSn)$_2$Ir(PMe$_3$)$_2$]$^+$ without any further optimization.

It is obvious that there are only two roughly equally strong interactions between the transition metal fragment and the stannylidinium (NOCV1 and NOCV2, ca. -73 kcal mol^{-1} each). NOCV1 is a complex involving numerous SFOs of which only the dominant ones are depicted. Essentially it involves donation of a Ir d(z^2)-orbital into a p-orbital at Sn, however the d(z^2) orbital is naturally ill-suited for efficient π-interactions and furthermore lacks ideal relative orientation. NOCV2 however again represents a rather clear π-backdonation interaction from the metal to the Sn-atom. The further NOCV (e.g. NOCV4) represent σ-interactions from the [MeSn]$^+$ moiety towards the Ir-fragment, however these interactions are rather weak.

Overall, this second stannylidinium moiety appears to be bound by a twofold interaction when additional bonding contribution is neglected.

The averaged bond order between Sn-Ir-Sn would accordingly be around 2.5 for each Ir-Sn-fragment.

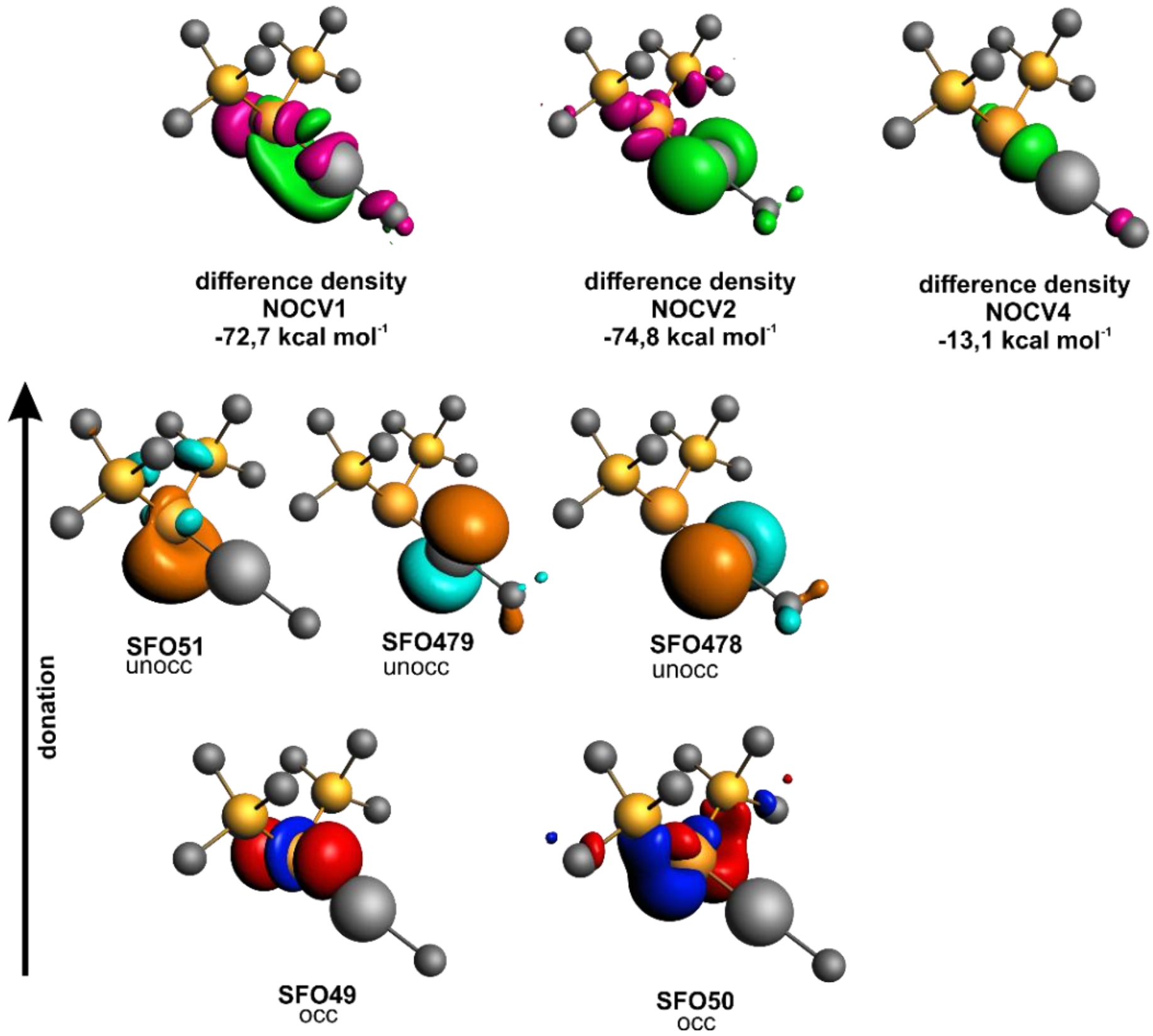

Figure SI42. Relevant NOCV-interactions of [RSn]$^+$ and [Ir(PMe$_3$)$_2$]$^-$.

NMR chemical shift calculations

On the basis of the optimized structures of **1**, **2** and **3** NMR chemical shifts were computed at SO-ZORA level with ADF, including the crucial exchange correlation kernel (PBE density functional, basis sets: TZ2P Ir, Sn, P, TZP O, N, C, H).[36-42] The obtained ^{119}Sn NMR shieldings were converted to chemical shifts (δ, in ppm) relative to the shieldings of tetramethyltin (σ[^{119}Sn] = 2867.21) calculated at identical computational level.

Compound **1**
Sn(1)

UNSCALED: TOTAL NMR SHIELDING TENSOR (ppm)

CARTESIAN AXIS REPRESENTATION

total shielding tensor

1286.217 -1345.535 -310.340

-1345.535 3630.892 206.022

-310.340 206.022 594.303

isotropic = 1837.137

PRINCIPAL AXIS REPRESENTATION

Principal components:

416.961 824.031 4270.420

Principal Axis System:

0.580 0.700 0.417

0.192 0.380 -0.905

0.792 -0.605 -0.086

Sn(2)

UNSCALED: TOTAL NMR SHIELDING TENSOR (ppm)

CARTESIAN AXIS REPRESENTATION

total shielding tensor

2217.573 -1372.562 -1332.196

-1372.562 1745.626 823.139

-1332.196 823.139 1524.448

isotropic = 1829.216

PRINCIPAL AXIS REPRESENTATION

Principal components:

413.920 816.609 4257.119

Principal Axis System:

0.720 0.120 0.684

0.384 0.752 -0.536

0.579 -0.648 -0.495

Compound **2**

Sn(1)

UNSCALED: TOTAL NMR SHIELDING TENSOR (ppm)

CARTESIAN AXIS REPRESENTATION

total shielding tensor

2993.681 -272.464 -16.732

-272.464 3750.700 300.867

-16.732 300.867 3450.239

isotropic = 3398.207

PRINCIPAL AXIS REPRESENTATION

Principal components:

2893.312 3305.476 3995.833

Principal Axis System:

0.923 0.303 0.238

0.350 -0.400 -0.847

-0.161 0.865 -0.475

Sn(2)

UNSCALED: TOTAL NMR SHIELDING TENSOR (ppm)

CARTESIAN AXIS REPRESENTATION

total shielding tensor

3118.831 170.345 239.245

170.345 3814.549 139.700

239.245 139.700 3318.991

isotropic = 3417.457

PRINCIPAL AXIS REPRESENTATION

Principal components:

2954.231 3378.671 3919.469

Principal Axis System:

-0.848 0.445 -0.289

0.083 -0.426 -0.901

0.524 0.787 -0.325

Compound **3**

Sn (1)

UNSCALED: TOTAL NMR SHIELDING TENSOR (ppm)

CARTESIAN AXIS REPRESENTATION

total shielding tensor

765.920 191.079 637.709

191.079 2794.126 -233.741

637.709 -233.741 2879.505

isotropic = 2146.517

PRINCIPAL AXIS REPRESENTATION

Principal components:

561.002 2730.026 3148.523

Principal Axis System:

0.955 0.223 0.194

-0.110 0.878 -0.465

-0.274 0.423 0.864

Sn(2)

UNSCALED: TOTAL NMR SHIELDING TENSOR (ppm)

CARTESIAN AXIS REPRESENTATION

total shielding tensor

2006.785 750.686 -876.893

750.686 2161.522 626.537

-876.893 626.537 2220.535

isotropic = 2129.614

PRINCIPAL AXIS REPRESENTATION

Principal components:

613.184 2775.874 2999.784

Principal Axis System:

0.637 0.326 -0.698

-0.534 0.840 -0.095

0.556 0.434 0.709

Minimum energy path computations

The minimum energy path (MEP) of the trimethylsilyl azide reaction with cation $[(PhSn)_2Ir(PMe_3)_2]^+$ was investigated using the Nudged Elastic Band (NEB) method [43] in conjunction with the r^{2}SCAN-3c method [44] as implemented in ORCA6.0.1.[18, 19, 45] The r^{2}SCAN-3c method is a versatile and computationally efficient approach often referred to as a "Swiss army knife" of computational chemistry.[44] The method combines the r^{2}SCAN meta-GGA density functional with a tailored triple-ζ Gaussian basis set (def2-mTZVPP/J), an auxiliary basis set for the resolution-of-the-identity (RI) approximation [46], the D4 dispersion correction for long-range London dispersion,[47] and a geometric counterpoise correction to account for basis set superposition error (gCP).[46, 48]

The reactants and products were fully optimized at the r^{2}SCAN-3c level of theory starting from experimentally determined structures obtained by single-crystal X-ray diffraction (XRD) when available. The structure of $[(PhSn)_2Ir(PMe_3)_2]^+$ was generated based on the structure of $[(Ar^*Sn)_2Ir(PMe_3)_2]^+$ (**1**) by exchanging the Trip substituents against hydrogen atoms. Trimethylsilyl azide and trimethylphosphine were added using the software *chemcraft_v1.8*.[29] The minimum energy pathway was then computed. The intermediate from step 10 was separately generated and optimized.

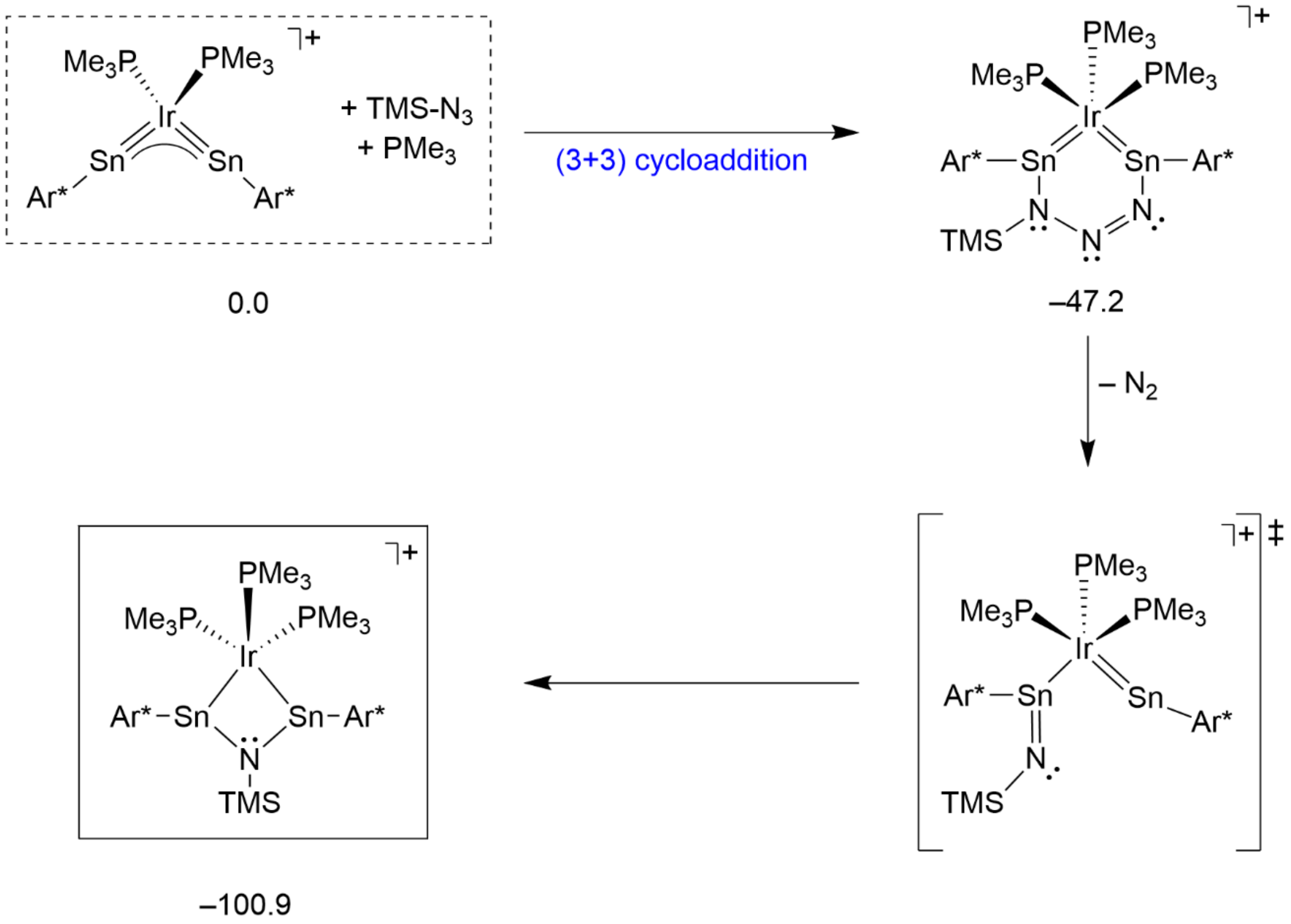

Scheme SI1. Proposed mechanism for the formation of **3** (energies are given in kcal mol^{-1}).

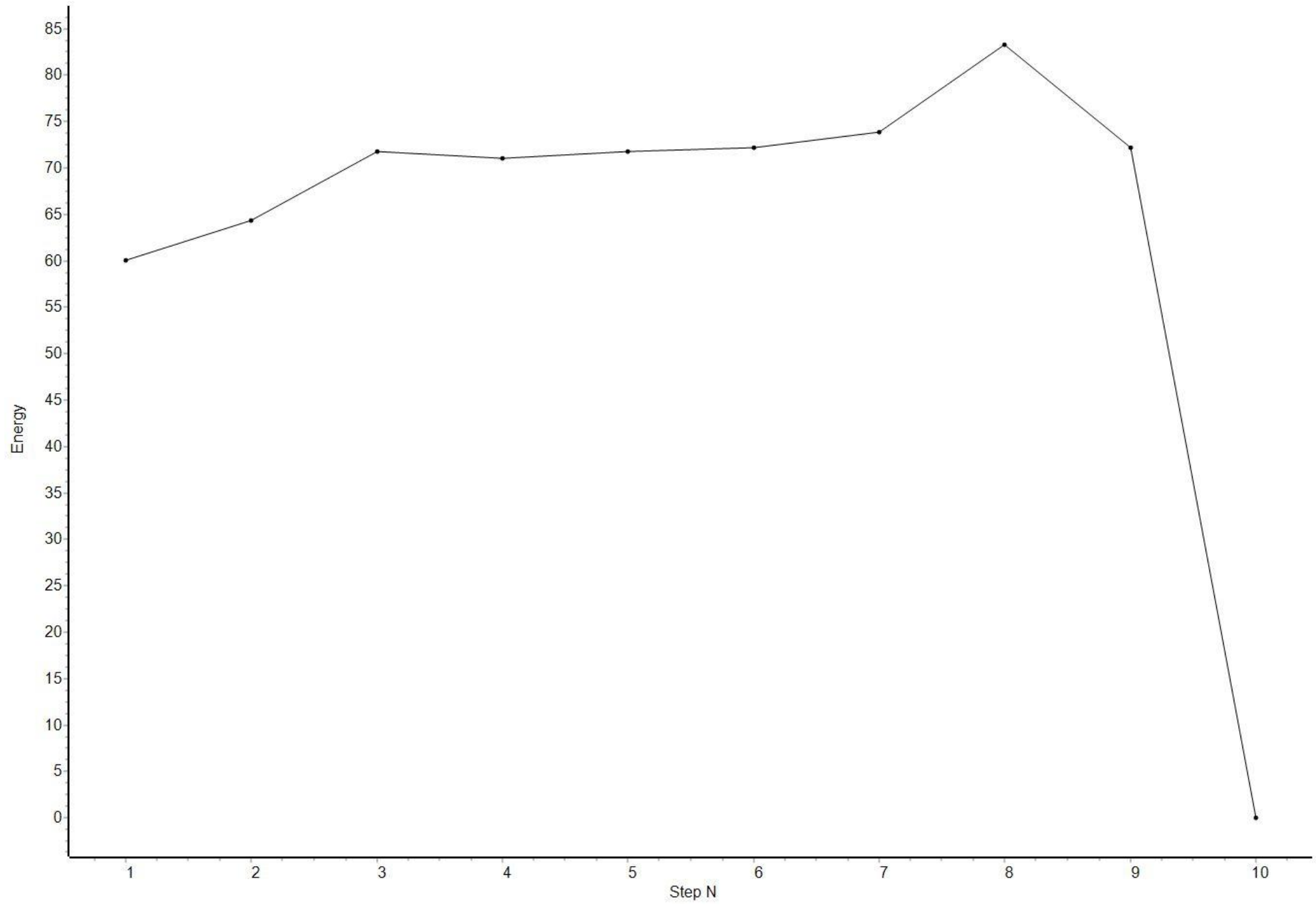

Figure SI43. Energy profile (Energy in kcal/mol) of the minimum energy path of the reaction of $[(PhSn)_2Ir(PMe_3)_2]^+$ with TMS-N_3 (1/2).

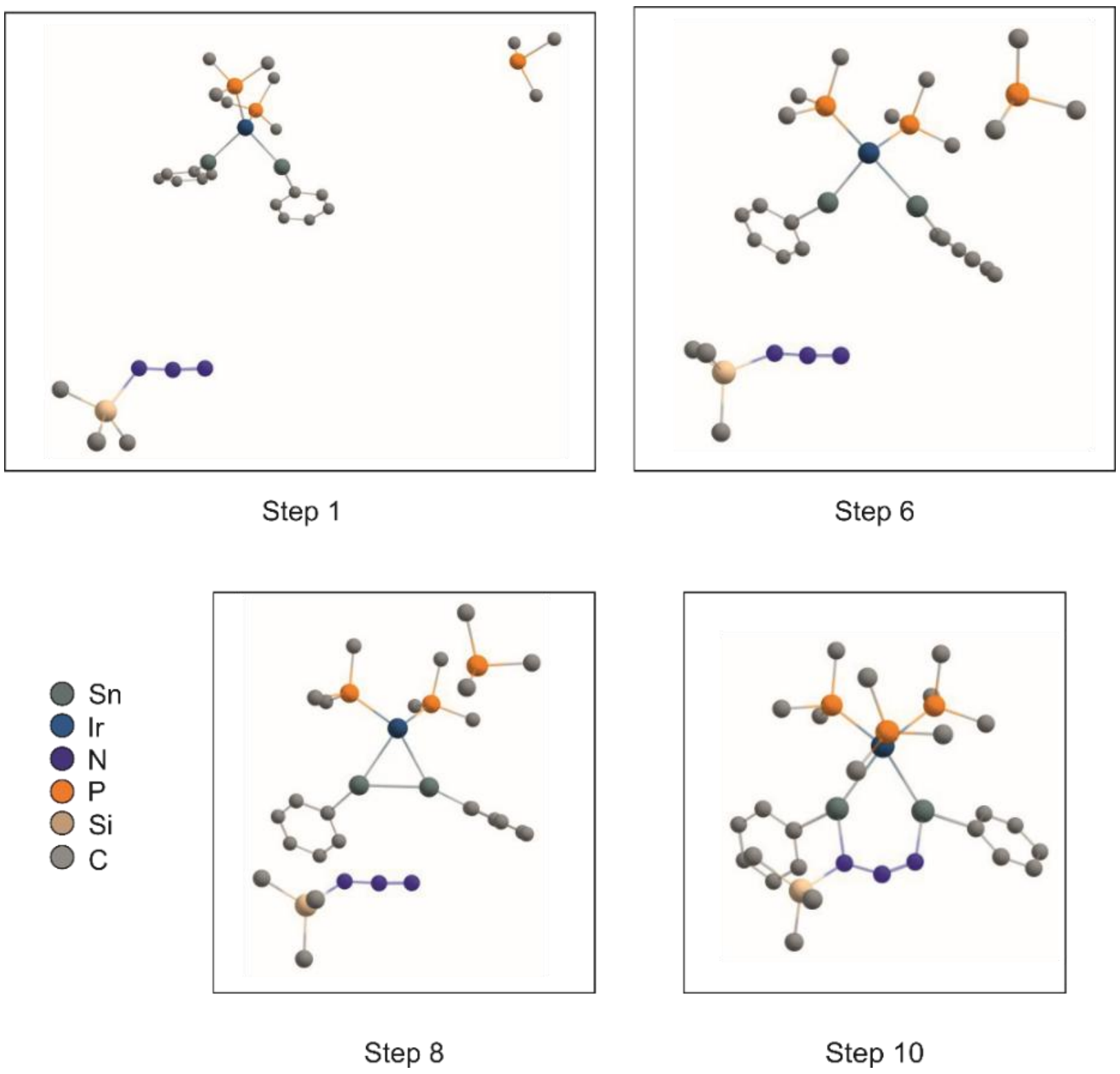

Figure SI44. Depicted molecule geometries of the minimum energy path for the reaction of $[(PhSn)_2Ir(PMe_3)_2]^+$ with TMS-N_3 (1/2). Carbon-bound hydrogen atoms are not displayed.

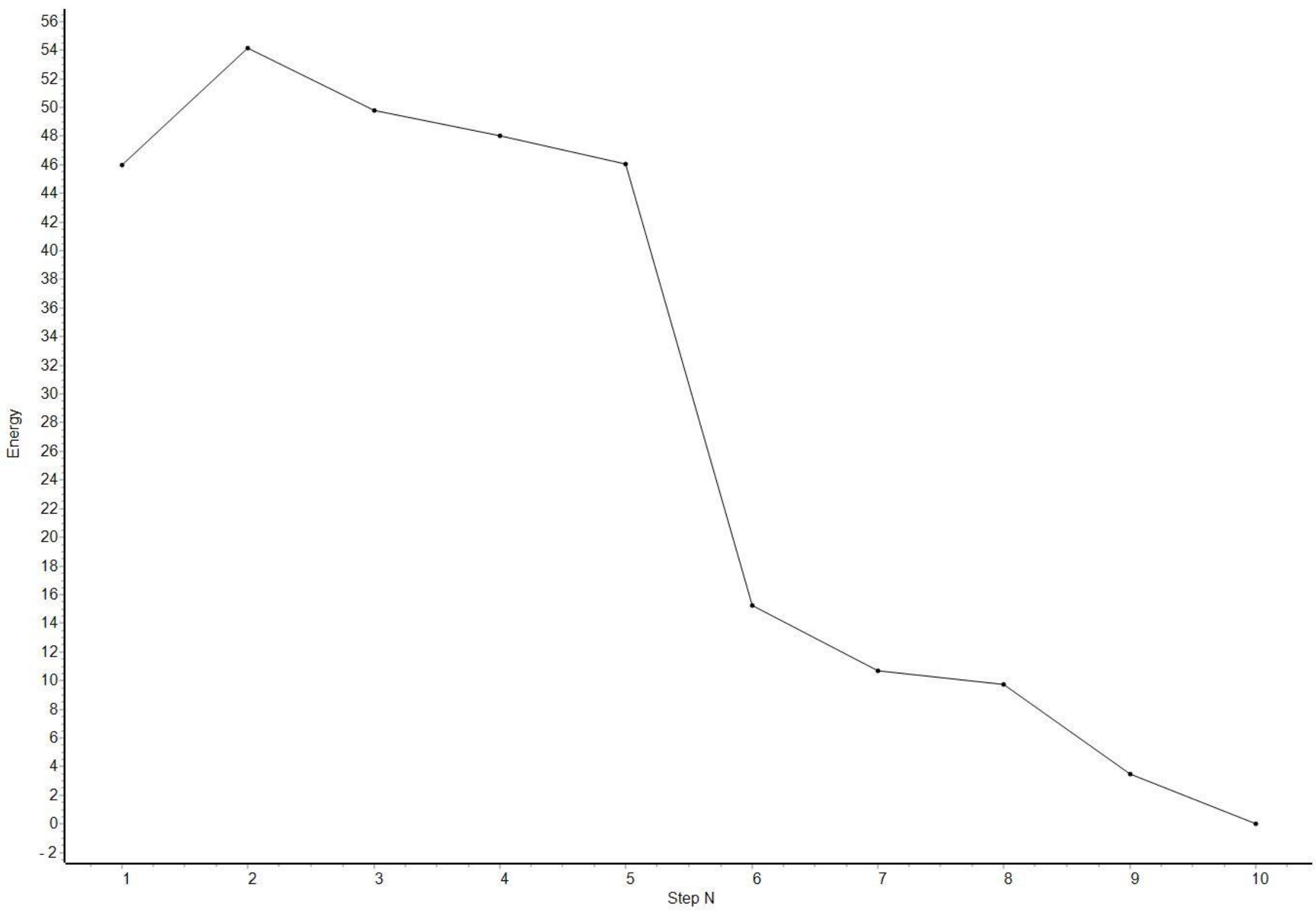

Figure SI45. Energy profile (Energy in kcal/mol) of the minimum energy path of the reaction of $[(PhSn)_2Ir(PMe_3)_2]^+$ with TMS-N_3 (2/2).

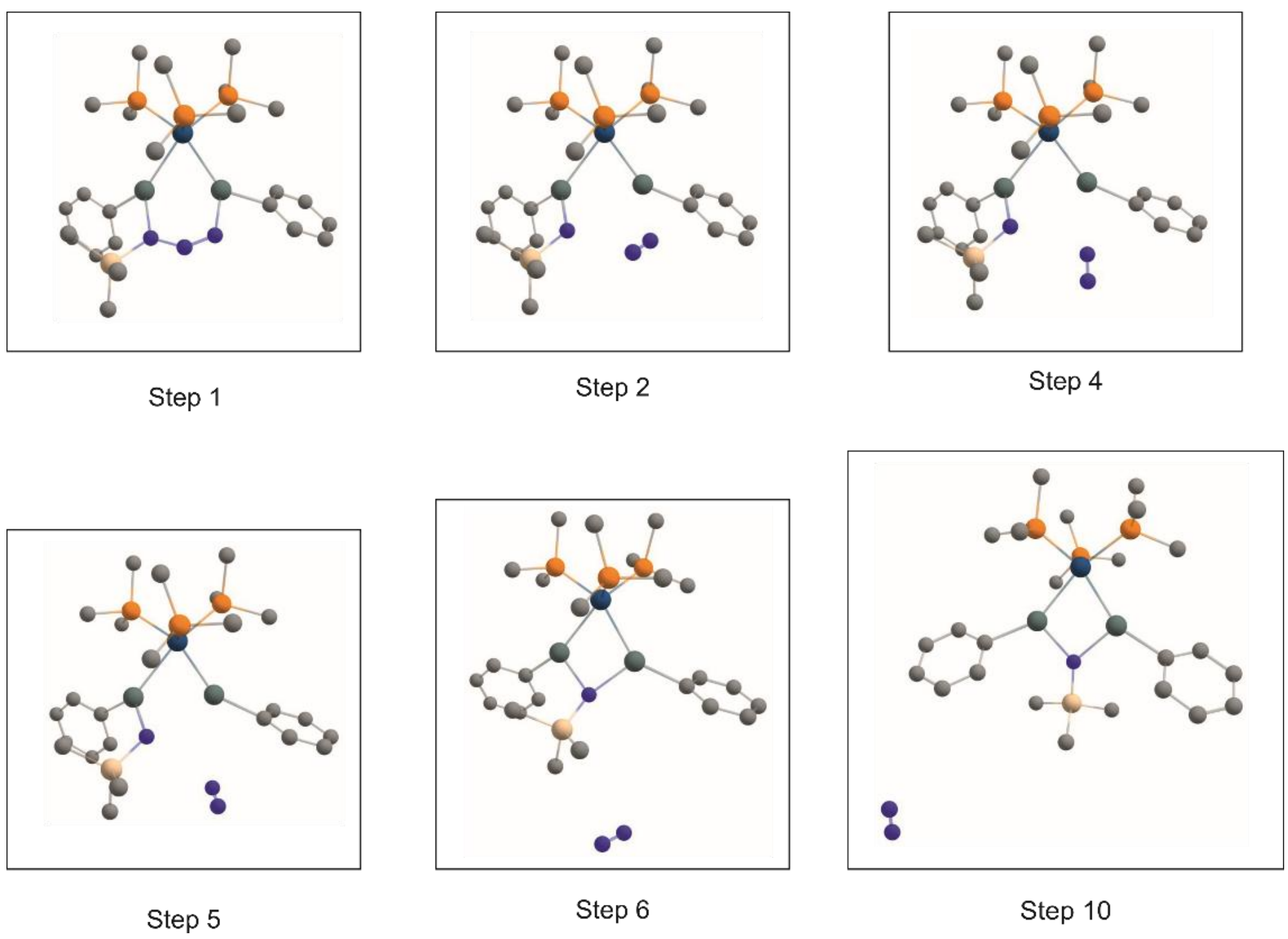

Figure SI46. Depicted molecule geometries of the minimum energy path for the reaction of $[(PhSn)_2Ir(PMe_3)_2]^+$ with TMS-N_3 (2/2). Carbon-bound hydrogen atoms are not displayed.

Results of structure optimization

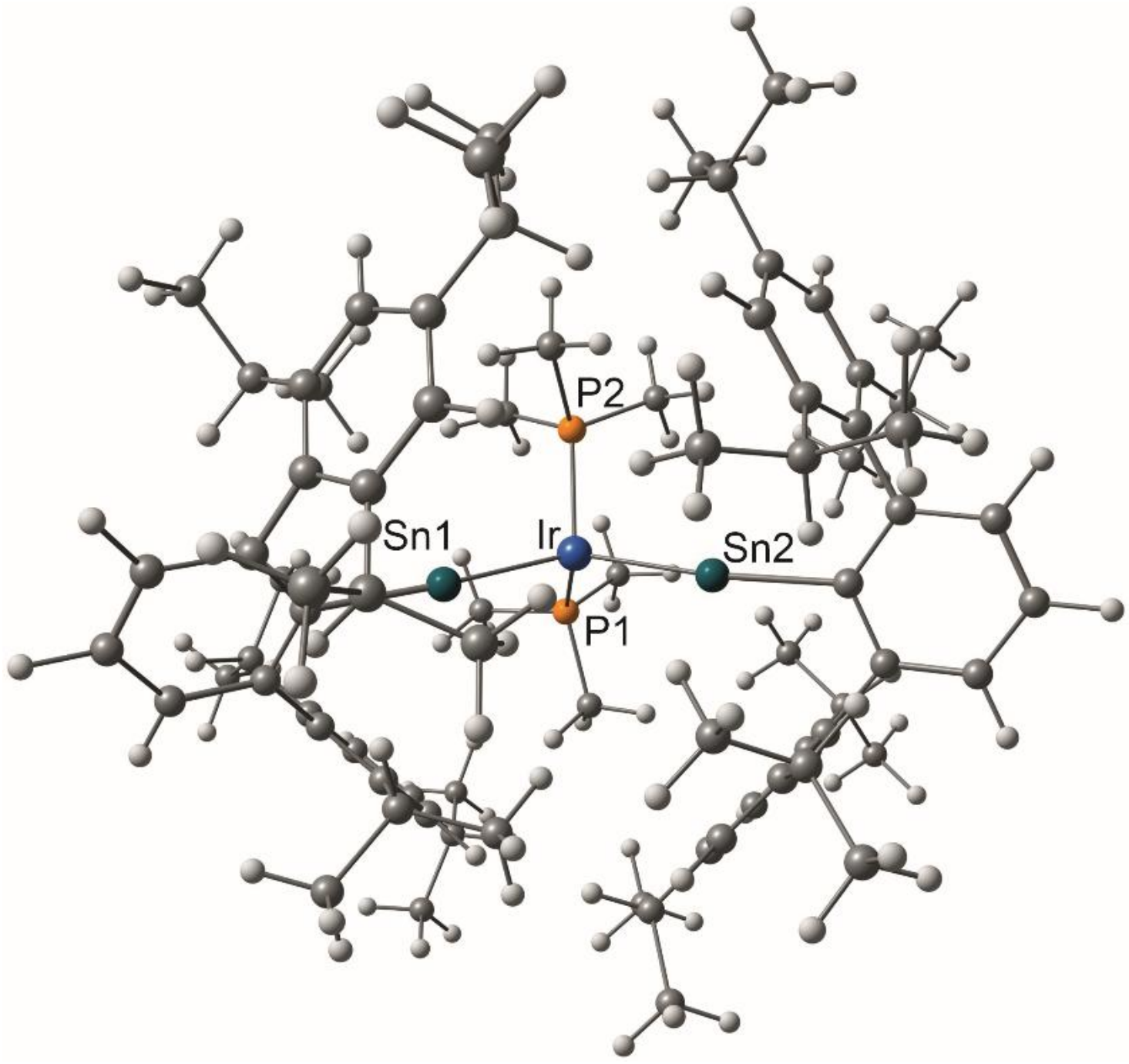

Figure SI47. Optimized structure of cation of **1**.

Table SI3. Coordinates of optimized structure of cation of **1**.

Ir	8.27637995363462	4.94186457084518	4.99275454977148
Sn	10.04831749157620	6.61011144521465	4.96542449135353
Sn	6.15439918285734	4.90946105938381	3.80232408600630
P	9.31146220092669	3.01827729344305	4.40072871495238
P	7.73930644482833	4.67869590901914	7.17591341174020
C	11.86878985790462	7.76573367717073	5.12328567342455
C	4.23180041687853	4.54344570430992	2.88761973214520
C	4.17923638802381	4.09526093354444	1.55282313454267
C	11.74021852755061	9.01433186021458	5.76762985028250
C	13.07012634492944	7.38452452222958	4.49215517311859
C	5.44994370010485	3.74502751826869	0.84970078557705
C	10.40241458846171	9.35126217715037	6.33869260795310
C	13.52161754693300	3.69394443812799	2.28156918307262
C	11.22758558478277	8.63458927602585	8.65093285148461
H	12.20230555939474	8.77965898029134	8.14279789635784
C	14.16067747810449	8.27849379613208	4.53380142026077
H	15.10182659807078	8.00150999416263	4.03409600505708
C	3.06248204335849	4.90950052798304	3.58844424377539
C	5.78748701550555	6.22760600502563	0.31797795208355
H	4.96415204533194	6.29852565420249	1.05880004227244
C	14.61789939503054	5.17415528094482	5.67271777493617
H	14.91941425714847	6.23451214693195	5.79569002646037
C	14.15556054153061	3.87371943065606	3.52350861299286
H	14.80791159114911	3.07148132451368	3.89837675561454

C	2.82894676037732	4.52299037416005	6.07308160300548
C	14.04792527088922	9.51414797250047	5.19152537028546
H	14.90730087882976	10.20113399267550	5.21031883633636
C	13.98795224596701	5.04133014734095	4.28798017965507
C	13.56532420776629	4.88292789906780	6.76209223290018
H	12.67060882208273	5.52641072928820	6.64165425189349
H	13.98028478980866	5.06857577620106	7.77360831124110
H	13.23562653989038	3.82528166279472	6.71488751392011
C	12.84286096016885	9.88716851865094	5.80894452981016
H	12.74911982873865	10.86150984431166	6.31211258621067
C	9.36253307125634	9.77325382158244	5.46056485767871
C	10.14569808738600	9.17010629840792	7.72041354996284
C	6.25405788465417	4.77452297230143	0.29167523617757
C	5.78016229309819	0.94492414812092	2.78817568925736
H	6.78790212496738	0.52013917375871	2.60025928327370
H	5.20068606653665	0.21074356727170	3.38373535498992
H	5.91421296385478	1.85589509847289	3.41014710297312
C	5.07096998907065	1.28757544459941	1.46498057135757
H	4.07451331258841	1.69889688629216	1.72461039510224
C	6.88427624003656	7.20633521383730	0.75967786399168
H	7.71115451180320	7.26086362323598	0.02335431064140
H	7.32637953373766	6.90779196891150	1.73354086691239
H	6.47260980909665	8.22978988843265	0.86922763085327
C	8.59526886037210	1.48952557863695	5.11728171739320
H	7.52226174397912	1.42642840620639	4.86425609077009
H	8.69182377749501	1.52408293211050	6.21913563533593
H	9.11718162446013	0.58586039752559	4.74180133188806
C	12.66603206503378	4.70974071374640	1.82138076731524
H	12.16432924915059	4.58752317170943	0.85103864362121
C	13.17087391625181	6.08267307618216	3.76786110857533
C	3.76209192407657	6.65983803336532	5.25973301741537
C	9.06714779343778	3.97562317741782	8.22749727300385
H	9.33241603022005	2.96768296672399	7.85590634867130
H	8.74731043672717	3.89556079656845	9.28647559556116
H	9.96618955673436	4.61396682349964	8.16026459778583
C	3.94614207888620	7.05178037176045	6.59656477515676
H	4.37111549887424	8.04314639588604	6.81269430291201
C	2.92594596770052	4.00095798684485	0.91555187042621
H	2.87384334276571	3.64601602078865	-0.12498842752453
C	5.84936710397298	2.38598659378348	0.75093960279164
C	8.08078731984667	9.98899272922078	5.99347536325412
H	7.27639328150248	10.31833666579984	5.32023181908920
C	9.65508882004832	10.09447618354785	3.99668551097216
H	10.62481666185837	9.62111575543094	3.73877701154160
C	1.82070304782892	4.81904273955391	2.93126088266850
H	0.90201027418297	5.11033466450299	3.46336943548717
C	3.20072165261998	5.37678829413963	4.99973966719490
C	5.19032373225178	6.61491537045335	-1.04838683166469
H	5.95659422725793	6.55087091682008	-1.84851499973727
H	4.79979147692856	7.65304843478410	-1.02997041250379
H	4.35725350461337	5.93863973333658	-1.32548566153888

C	7.79910188090312	9.81818887626610	7.36179386633450
C	13.79525780075931	2.45133727016153	1.44526653516101
H	14.54885320848977	1.85188701689151	2.00099453781294
C	12.48865278156196	5.91245903031025	2.52996729159443
C	8.84843677736640	9.41402831488654	8.20494629081310
H	8.65516941776725	9.27332039950967	9.27675346167139
C	11.06603810503008	2.85100966348334	4.88195785029613
H	11.47452024917577	1.85648135915106	4.60949606566681
H	11.15971829579497	3.00002108546910	5.97323415411679
H	11.65544304856282	3.63475312526768	4.37203598366446
C	15.87152263934059	4.31388921499180	5.87901126568543
H	15.63400161812562	3.22990595701695	5.89290138744813
H	16.34343710611799	4.55307811661350	6.85291713271540
H	16.62374014691308	4.48785430786016	5.08370962042151
C	7.44387554420234	4.42026349389916	-0.37533762035897
H	8.06358228581885	5.21724640185446	-0.81091584694667
C	2.29234463446731	3.11989966745093	5.79834403524056
H	1.70434039139317	3.17460169182964	4.85962072226201
C	4.04493723207153	7.64036553567223	4.12291136950915
H	4.33407399556065	7.05090264537280	3.22564114282226
C	7.83692223762893	3.07974337484952	-0.52335667240784
C	9.05695725521768	2.66005313275682	-1.33221381843208
H	9.42640787887733	1.71318895940155	-0.87834071318596
C	4.84723918800570	0.03604432874044	0.60255995265167
H	4.35210675022489	0.29131886009398	-0.35547044929065
H	4.20620422020818	-0.69427131835851	1.13643117182290
H	5.80014436068155	-0.47968591677388	0.36376981640987
C	7.26046234597035	6.19309661533075	8.07735057284915
H	8.08977107203608	6.92163140731943	8.02861785540639
H	6.99684741283544	5.98083845760385	9.13386855439577
H	6.39162066829024	6.63549297224973	7.55626843364720
C	3.02381216137195	4.97184847993211	7.39167491454360
H	2.73621387266396	4.31388574579431	8.22223754589699
C	6.18036345513765	9.70916414947789	9.35164099474614
H	6.45560473919503	8.65536048909829	9.55645276087539
H	5.11736342268640	9.84144172443372	9.63439527436187
H	6.78227039955579	10.35431803917113	10.02475320351050
C	7.02911288285277	2.08311745342185	0.05555538640304
H	7.34712589613982	1.03221296851668	-0.03404942929749
C	8.63961902534619	2.34718611392973	-2.78445982331419
H	8.24792870094393	3.25908861114565	-3.28119637384502
H	7.84235885586046	1.57838758200632	-2.81881320259241
H	9.50260105300253	1.97750390647543	-3.37532233839667
C	11.04043645841818	7.11852754117040	8.84553078544412
H	11.01265072851947	6.59424356590976	7.86654286581786
H	10.08800841608268	6.89896834982175	9.37049190681444
H	11.86858627530748	6.68016425447476	9.43853504401906
C	9.34645316508886	2.65481204498511	2.61288946493899
H	8.31324703839593	2.64692014179613	2.22320681115633
H	9.83800078436385	1.68601253444557	2.39545908049139
H	9.89431911309129	3.46921904041370	2.10370845219864

C	1.75719414654464	4.36552569966732	1.60273712455716
H	0.78306004033584	4.30106479667708	1.09502917769513
C	6.38956364431009	10.08421307422237	7.88040552448700
H	5.71641622696902	9.43926252774729	7.27056104746529
C	3.58108079946448	6.23007677533138	7.67634136431280
C	3.45548730939924	2.14176647992812	5.53789284442095
H	3.07324865595577	1.14262645227995	5.24549366791291
H	4.07700144813042	2.01727361470863	6.44751765845284
H	4.11106390279394	2.50298373800542	4.72103746035205
C	10.20750289336377	3.67171410423779	-1.29338474348597
H	11.10625063565954	3.26129525071338	-1.79545686368160
H	10.48067613124126	3.92522840867341	-0.25078649876221
H	9.94547650352080	4.61588997993626	-1.81390221644444
C	12.54237385427602	1.57585533193108	1.27170668880163
H	11.74581886873612	2.11990041681395	0.72456907935499
H	12.12983595747998	1.26541457441825	2.25211249268860
H	12.77955714211449	0.65896059564386	0.69458799299965
C	6.34195066343042	3.55488755355754	7.53666374331588
H	5.41667322168208	4.00905063325217	7.13506478840120
H	6.22288432250590	3.38890720956719	8.62707970270857
H	6.50589995674584	2.58506587649280	7.03281847181028
C	11.29016912791958	9.37061030305878	9.99830929179571
H	11.40414467960739	10.46369721814910	9.85598405690918
H	12.15093246129679	9.01041799341106	10.59695551250815
H	10.37750182713119	9.20174916015644	10.60647384486189
C	5.97384590502596	11.54765357446351	7.62916964942145
H	6.05490370086220	11.82446636300408	6.55960955241952
H	6.62212887011033	12.24021102011824	8.20469417095413
H	4.92463649345887	11.71852865064685	7.94493115221640
C	1.36007825715505	2.58537769698075	6.89444470599637
H	0.54256899091985	3.29870111310974	7.12118970230459
H	1.90472156184139	2.37890848139917	7.83915058053056
H	0.90046203742869	1.62946577767149	6.57295762748598
C	14.40693823143554	2.82275042463214	0.08115530007590
H	14.68176897827714	1.91163204645037	-0.48834951725216
H	15.31820827829721	3.44141349459813	0.20289541071265
H	13.68821060411500	3.39945271863935	-0.53760521986638
C	5.19425800066557	8.61074503536834	4.41729537653411
H	6.10892961619290	8.07529449697523	4.74692043696911
H	4.92116129528940	9.34564974906287	5.20153195141937
H	5.44629945345832	9.18844676009805	3.50618791187290
C	2.75070908952718	8.39023936532770	3.74908476321956
H	2.92632133452879	9.08051380321433	2.89880913879920
H	2.38518178355924	8.98731448216256	4.60994698725836
H	1.94742345265721	7.68497799508723	3.45905390096638
C	9.84349622421038	11.61420464636150	3.82578567096806
H	10.10795988019817	11.86422604269554	2.77799276737132
H	10.65066893130213	11.99218071174303	4.48426206302590
H	8.91299377889545	12.16023478284706	4.08552973134594
C	3.94541227543551	5.64250360606075	10.14443552887847
H	3.02656235866323	5.03613281133055	10.28285912605733

H	4.19308100610148	6.08312539474157	11.13079729766513
H	4.76515910260262	4.95192823883984	9.86115570238289
C	3.74789829878185	6.74685436432609	9.09810969786436
H	4.66194982322720	7.37829065291724	9.09624326464410
C	8.60238636141955	9.53830040541090	3.02757513962663
H	8.91938475285636	9.69572918424098	1.97732170715579
H	7.61867380419656	10.03415612957677	3.15196956191807
H	8.44280048865554	8.44948349877618	3.17792668286523
C	2.56412102016766	7.66309568111356	9.47085494448961
H	2.45405506894298	8.49510871410699	8.74687664361026
H	2.70375450807339	8.09985041287174	10.48099907740747
H	1.61237960376741	7.09249915355970	9.47232425580588
C	10.49184318531695	6.61629781702696	1.07888254138856
H	9.90492700885646	7.50078094305274	0.76403803744235
H	9.81968141853046	5.95860652356136	1.66879434312693
H	10.78420657347278	6.07706550826099	0.15718570138895
C	11.70942830858126	7.06261292179015	1.89504843364879
H	11.33269196728811	7.71260841233483	2.71309580217539
C	12.66382310677557	7.93710735822742	1.05788910796309
H	13.10108305215035	7.34882971711793	0.22484877320374
H	13.49832226463695	8.32380846983593	1.67543572719403
H	12.12583456234354	8.80452181773002	0.62332246080995

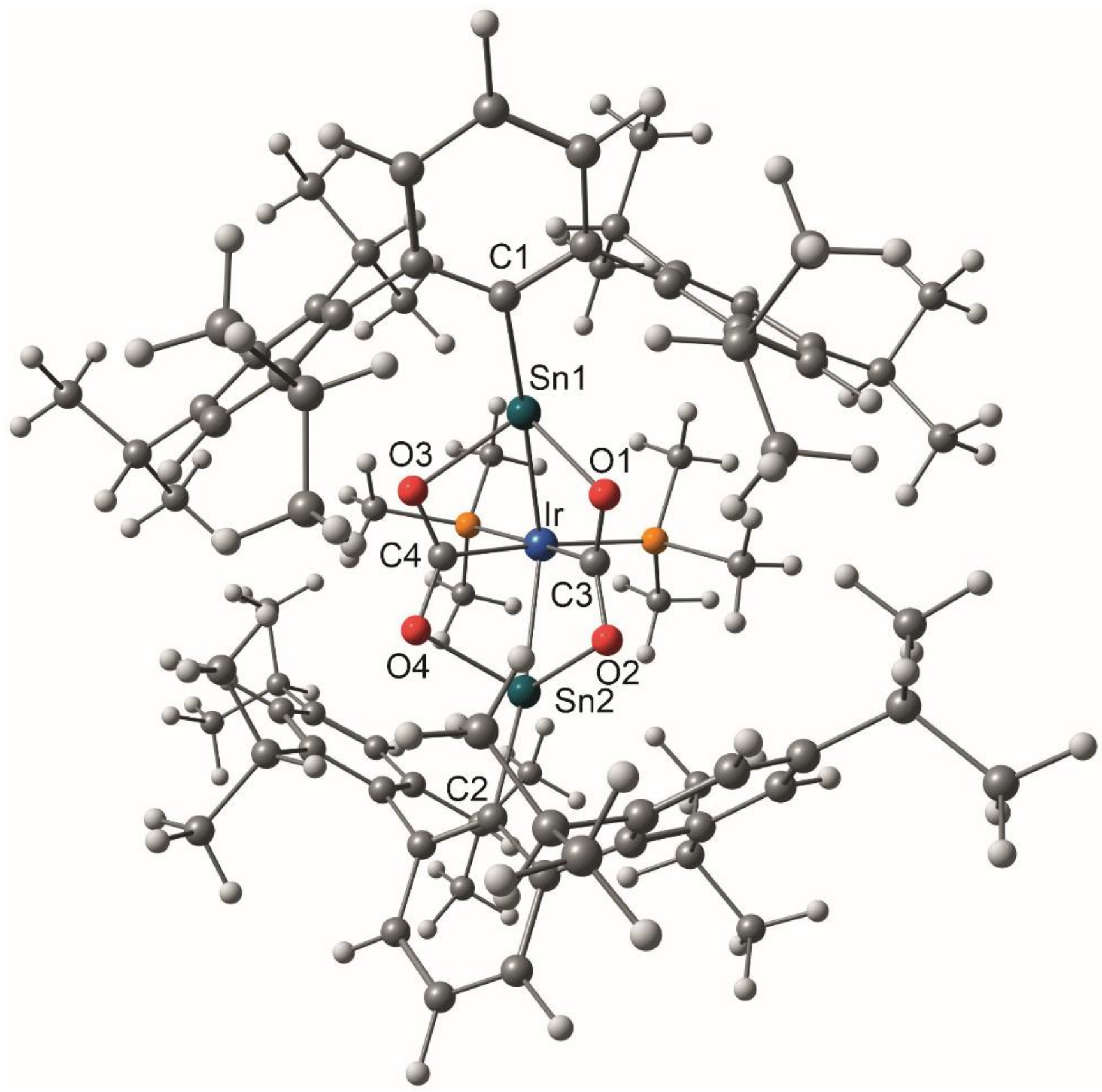

Figure SI48. Optimized structure of cation of **2**.

Table SI4. Coordinates of optimized structure of cation of **2**

Ir	12.42728285120946	6.21362525260567	4.63022034520938
P	11.50765613169915	4.12231717294028	4.05622427343693
Sn	10.54641035034103	8.01345030885501	4.57959523968715
Sn	14.68863227754772	6.31556986360809	3.35629858482311
P	12.83222859914616	5.79062436294559	6.91293690060210
C	14.01245190339183	12.05995853887394	7.77601525237767
H	14.74234979155564	12.49030572062989	7.05622369467721
C	14.63539674158157	10.77985119152930	8.35575243065347
H	14.80839972850462	10.02893008277458	7.56057556695463
H	13.97912435711151	10.31923055885570	9.12372011421157
H	15.60671402881790	10.99887563050227	8.84417234795005
C	13.75655527166461	13.11119835572822	8.87270121914408
H	13.34143735601862	14.04510338479948	8.44420257936025
H	14.69469436117477	13.36404908006289	9.40843504834737
H	13.03009418021523	12.73465647006495	9.62311771935048
C	8.72660982736930	10.97167474918995	8.65624173739611
H	8.39469578989084	11.94297530779185	8.23921615685216
H	9.46547711418333	11.18589593841499	9.45578599386702
H	7.85009129692235	10.47969038114534	9.12596865283303
C	9.33512583148567	10.08263799906158	7.55560740854082
H	8.53937824550385	9.90707745957640	6.80238995107669
C	9.75069026129622	8.71051708746825	8.10878000025332
H	10.14893160114806	8.05564682770583	7.30092906226159
H	8.89216862603741	8.18296573093226	8.57080320160094
H	10.54857167881264	8.79573059414416	8.87456997671086
C	16.47209254179660	6.65436211933456	2.22010292853118
C	17.60384664224027	6.95519651078522	3.01030799055681
C	18.82790478672588	7.18436579589201	2.35047764272068
H	19.72793744486780	7.40567909236822	2.94253696166049
C	18.90058520829531	7.10494222461301	0.94996417560508
H	19.86256499729600	7.27878063000420	0.44466688250288
C	17.76638094092091	6.77869616542883	0.19079970806271
H	17.83536087342268	6.68829562781799	-0.90351568523233
C	16.52444995573692	6.53194070452768	0.81607703784653
C	15.32047844813919	6.11953794755609	0.02950038278083
C	14.52766257091109	7.09844713571780	-0.62302339251176
C	14.87595318094858	8.58064901625280	-0.54113052317553

H	15.57932388714435	8.70416438444919	0.30939517986805
C	15.60460340663912	9.04421732453699	-1.81756744752189
H	14.95373802750161	8.92100143156871	-2.70822275973982
H	16.52840728222168	8.45885043363709	-1.99672509163118
H	15.88559435679602	10.11510110162581	-1.74624820445204
C	13.77624580194787	4.38394644453434	-0.69677553842991
H	13.48882676910219	3.32073448173636	-0.73228232456312
C	18.91324471996963	10.01420063878042	4.56337348957438
H	19.60968475649048	9.29125116667144	4.09654783216754
H	19.25616520406416	10.18251430451925	5.60547771149863
H	19.00043901916018	10.97471576044481	4.01579310917995
C	16.51289370115932	10.57287808502563	5.13553365347225
H	16.56121038536927	11.49518999139781	4.52204499250575
H	16.80565856789683	10.85875726683758	6.16680248591101
H	15.46517729783684	10.21877148062448	5.14364900571340
C	17.45439300764964	9.50911849994631	4.54760506008792
H	17.15961996426209	9.35051483732359	3.48877800989473
C	17.47587495395959	6.94301334893029	4.50262880090938
C	17.43142375984006	5.68149402623842	5.17166720738699
C	17.31651825657582	5.67043608829084	6.57259032516536
H	17.33157977420067	4.70488429717554	7.10141893912635
C	17.23542526088604	8.07996304964894	6.64305181732600
H	17.14951738445240	9.01644671702582	7.21027154229807
C	15.79494569750286	3.66739294642920	0.65814638547247
H	16.65286856833738	4.16702135342569	1.15386117785099
C	15.01065811674211	2.91942535777025	1.74766385973261
H	14.11535365153501	2.40974043280210	1.33660916848908
H	14.65232293779531	3.62092660706720	2.53400591494698
H	15.63816217959452	2.15357875690883	2.24582694951763
C	16.37923236970224	2.69645472574348	-0.38415013486976
H	15.57852032155129	2.14126190787460	-0.91481082001328
H	17.04557752237166	1.95142352924277	0.09726253264782
H	16.96798700750300	3.24391820078912	-1.14655854425706
C	7.04090276037539	11.35015125421604	3.67214615962458
H	6.22987204468611	12.06649687646896	3.47218272717545
C	8.14420820029950	7.26191108933654	1.70406725913413
C	14.94492693910084	4.75037754494152	-0.00331212027927
C	16.76172785313036	3.22018409607213	4.94472313065796
H	15.68597000834282	3.49261595807949	4.93976852336560
H	17.03074002161661	2.93213487327290	5.98136931980972
H	16.88808059403211	2.31723887921365	4.31541547431224
C	19.12699582099109	3.99170399820282	4.40954849163157
H	19.29981290233849	3.06593991763991	3.82357515247016
H	19.49321980575111	3.82047903916421	5.44285120384802
H	19.73976276779571	4.79961856822642	3.96342263869788
C	17.63215797906408	4.36869466414772	4.41389361138959
H	17.34156716745664	4.53946531442548	3.35650566369740
C	17.22585380948708	6.85333186249298	7.33049982340168
C	11.33415769941204	3.96240051344732	2.24773749639002
H	10.57671892627379	4.69759100027480	1.91289605509150
H	11.00975694017154	2.94774638379603	1.94898179860671
H	12.28869406525624	4.20866787644364	1.74635452988571
C	12.45275836823566	2.63099067373777	4.54234815872724
H	12.02864308097561	1.71797486324712	4.07930147771965
H	12.41936630777250	2.51847898218748	5.64292701097722
H	13.50914502467446	2.73848973727017	4.23429323125041
C	9.83128140901281	3.74540657381827	4.66787316576334
H	9.13244474864406	4.51545119926508	4.28956704548280
H	9.81783881845165	3.77792891723875	5.77292585031125
H	9.48657190373446	2.74872696048712	4.32752956060870
C	11.39379414994309	5.24515699922169	7.90591188578265
H	10.54573476651404	5.93611477916313	7.74609578199893
H	11.63697535893873	5.20823070278016	8.98638183629988
H	11.08374261399993	4.23529938385639	7.57525255331891
C	14.10472305390078	4.55792948279700	7.34799099953072
H	13.84088019583463	3.56956971107726	6.93020285292709
H	14.22480537082841	4.47169090644962	8.44635242910856
H	15.06369812615848	4.88059614482474	6.90150506301232
C	13.42582289603124	7.28552440336814	7.77479239630055
H	14.39682802880943	7.57113196989024	7.32482789671351
H	13.56128924509406	7.11182528718716	8.86006639143297
H	12.71981001203558	8.12146452640975	7.61371691167839
C	13.38558516937490	6.67794978373290	-1.32541111733966
H	12.76081300953577	7.42861431106719	-1.83329455957853
C	12.98687441293487	5.33200142243420	-1.37229194957333
C	13.23031149991008	8.12507002609724	4.69851300283618
C	12.16946023544234	6.97860732422984	2.72007312847837
O	12.44729708122754	9.12298835195785	4.92394272232631
O	11.15251923093689	7.74230158446689	2.47982013817470
O	14.43911900687930	8.31035747938382	4.26768456428586
O	13.13970638713502	6.89174932106676	1.87733185245913
C	13.64812254591712	9.45956408500952	-0.24482883508671
H	12.94819192630979	9.49265374872357	-1.10547837460362
H	13.96319852172291	10.50309320414255	-0.04231281732011

H	13.09108793515509	9.08043825732913	0.63310786177348
C	17.36895060581078	8.15371134527922	5.24255334431518
C	9.07790034608638	9.53159450364809	4.20023140801164
C	7.81644207959235	7.66522328977921	3.03362886348792
C	7.27804288522009	6.72788189679654	3.95884948793169
C	9.20110158383368	10.84506191093989	4.69874378925417
C	8.16369271668986	11.75690599712746	4.40842437439828
H	8.24165604382128	12.78723106396967	4.78738277868626
C	7.00272503429095	5.41996695415821	3.51213820904711
H	6.53316896203117	4.70198098925977	4.20366748289593
C	6.93270051773711	10.03017465949089	3.20609481292539
H	6.03680843875533	9.70607446462547	2.65626989046622
C	7.27071315215118	5.00984582099824	2.19629634057251
C	7.88045481667834	5.93453168072967	1.32375546815558
H	8.11629118093870	5.62472390529008	0.29466691165205
C	6.84408073582052	3.62922389101260	1.71427575065107
H	6.40689403173358	3.10391446094321	2.59178437743092
C	8.02202820334858	2.78291675385954	1.20433783215608
H	7.67140766243135	1.78819823621211	0.86222921776684
H	8.77475189569172	2.62296141471899	2.00024229912485
H	8.53402549537946	3.26923198633961	0.34924603910143
C	5.73959705645652	3.75047252288842	0.64555247182824
H	6.12196461518747	4.24977476487109	-0.26887051960168
H	4.88172032414405	4.34415898368846	1.01922076070527
H	5.36525539796289	2.74896387398583	0.35072849495555
C	8.68683565112405	8.26431640014573	0.68872771135816
H	9.20462693851996	9.06088580400201	1.26232198900505
C	9.70759453075975	7.66479981893038	-0.29146961863885
H	9.23759182695259	6.93703293930957	-0.98479989189188
H	10.54189409535259	7.17007040282125	0.23870323660207
H	10.14244655565695	8.47020449617229	-0.91671762172662
C	7.52377296011499	8.91518782358182	-0.09010135186891
H	7.90989755821291	9.66789464375392	-0.80735658978076
H	6.80684178183702	9.42448467664291	0.58127504636188
H	6.96223702164839	8.15033997773471	-0.66583996084320
C	10.39441268804540	11.23062922429918	5.50826051028110
C	10.49320798820093	10.79118901435570	6.85486727647380
C	11.67327191693866	11.06415887542574	7.57017341766535
H	11.75065154807717	10.73571022599287	8.61907252907599
C	12.74400827583522	11.76454090267354	6.98933815489069
C	12.60694539948270	12.21060769642715	5.66418740575352
H	13.44668070220589	12.75421582793165	5.20610149227351
C	11.45383024702320	11.95846034405503	4.90307950175157
C	11.38565605141374	12.37276005830820	3.43506305439470
H	10.31621800235222	12.54171406531882	3.18937729682827
C	11.88614780960286	11.23548217207651	2.52064367012311
H	11.85583938754035	11.54978540335959	1.45771949785779
H	11.26768673013343	10.32187197302932	2.61197133290323
H	12.92728413555119	10.95252255008099	2.77392857296424
C	12.13349223917271	13.68165960639622	3.13677145863612
H	11.92830053727200	14.01118483332723	2.09838576723299
H	13.23282308417405	13.55851775148390	3.22418822609131
H	11.82650821635086	14.49624291357434	3.82327934438356
C	7.95694735391241	9.09445036575662	3.46255847766232
C	18.55628624116992	6.28560084068866	9.38556616836405
H	18.87763032868684	5.33100210182288	8.92388764045168
H	18.52427902485815	6.14153330795136	10.48468950997517
H	19.33365135495195	7.04489163727626	9.16124671945622
C	17.18339991805890	6.74952821692832	8.85203243280764
H	16.44548389405637	5.94887586523064	9.09177320037905
C	16.73390551849873	8.03073331171421	9.56250818858767
H	17.46968077070107	8.84998045176143	9.42602593049397
H	16.64220366738904	7.85509390144805	10.65274185760078
H	15.75634229565607	8.39233940818545	9.19136634581487
C	7.53286091658034	6.17422854762998	6.42754665181338
H	7.13828827907053	5.14191030023382	6.33272738076704
H	7.31092829840059	6.52041158755628	7.45693541718092
H	8.63629847879751	6.11965129722199	6.31498165401145
C	6.91705914488392	7.12657526397858	5.38862001570984
H	7.34235973146674	8.13402297290386	5.57487277580942
C	5.39063770403650	7.24647772423550	5.55410027024192
H	4.97085351800868	7.97734325661282	4.83471852714305
H	5.12927672348713	7.58317019809827	6.57814005335620
H	4.88951544922143	6.27256748963341	5.37647023801221
C	12.15147690203633	4.10699625826337	-3.41491076485836
H	11.26041853592203	3.85916226653670	-4.02770890801296
H	12.64281213670738	3.15242946778998	-3.13229826275022
H	12.86096174090913	4.67334334730851	-4.05047942237892
C	11.75482485481333	4.91595265079070	-2.16490244895541
H	11.26491801370374	5.85305435229196	-2.50834604687424
C	10.73579464810871	4.15238677192912	-1.30217372001885
H	10.43126455172137	4.75223583829710	-0.42312672946869
H	11.15183555872422	3.19252407715609	-0.93101114693966
H	9.82550632475462	3.91122031762657	-1.88888925694150

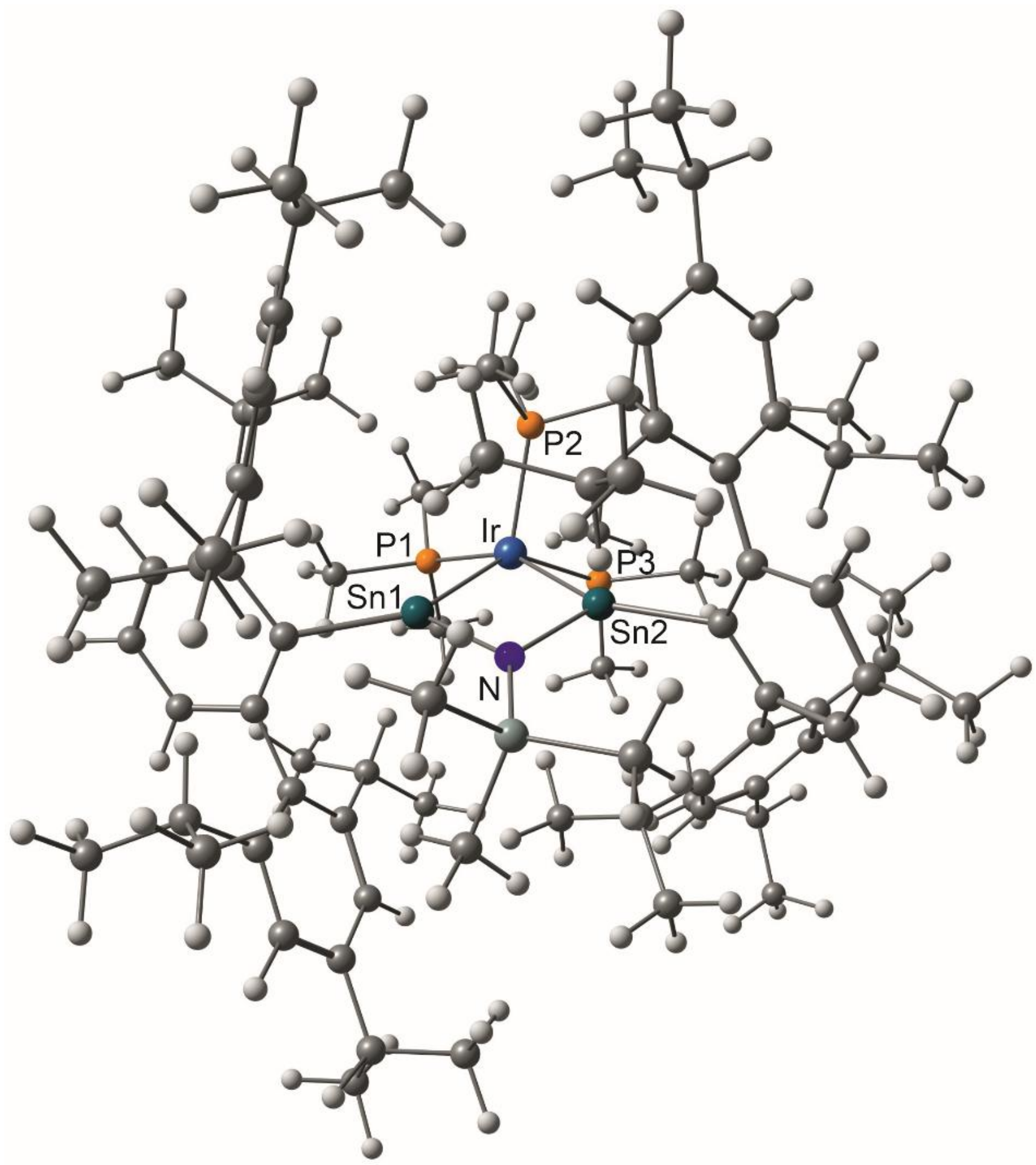

Figure SI49. Optimized structure of cation of **3**.

Table SI5. Coordinates of optimized structure of cation of **3**.

Ir	15.47056944731936	10.81219353452620	20.13308844077991
Sn	16.07267654676478	9.13587311175255	22.04949531629793
Sn	13.36080640404344	10.35652574191793	21.53420918186099
Si	13.57426379624599	8.43844083845101	24.52512409716904
P	14.83323717624547	9.74557288207237	18.16940815619884
P	17.65703686554356	11.45597589469046	19.75815530221043
P	14.65978627809643	12.92692410912808	19.74935229665182
N	14.17059437560852	9.08570309411328	22.98383478447824
C	18.46145561367003	7.13803243169851	21.96217607489730
C	17.88250364039600	8.12796791418682	22.80837440026340
C	17.10738982238442	5.32792036842767	18.87762453736190
H	17.26779620739969	5.32517591847803	17.78752121097904
C	19.85250675744160	6.89973809268728	22.02579781131253
H	20.28472718334977	6.11516029207065	21.38733050807418
C	8.80746433362729	10.03077276841384	23.24567332443901
H	7.88175427338981	9.79923425941386	23.79283160958964
C	11.72346540032609	8.70854892856297	24.79674271722331
H	11.09551064325830	8.20735157253912	24.03721175942063
H	11.42591454968680	9.77185999828528	24.82909966414423
H	11.47678371935256	8.26291785339383	25.78334900513616
C	17.93163750384954	8.70972641758396	26.15205070086817
C	20.66865433551042	7.60694101642709	22.91315206966785
H	21.75270405738130	7.42183343083500	22.94101374194936
C	13.06667875333076	12.25069399383699	24.60497402866076
H	12.81867570557896	11.23468909710218	24.23193814256650
C	11.36861236317491	7.43562321904874	20.22541041041366
C	18.04077002913013	10.92069263752323	25.06535079788664
C	18.68972596919242	8.75386453726257	23.79891864736847
C	17.85573128696888	6.21401179632530	19.66913005737749
C	12.38375148938112	13.26501574621988	23.70256117116655
C	17.57858379826641	9.34375520476546	27.35636916750165
H	17.40692465630228	8.73267418718625	28.25593109595550
C	18.15784791722762	9.50285310238247	24.98945630373138
C	18.92436232483825	7.05086591694204	18.97576595460316

H	19.40847820565222	7.68345382535409	19.74434288913918
C	9.24502557418944	9.18841205119550	22.21428784646785
H	8.63480870258239	8.32776224579159	21.90104758324042
C	9.50325907433577	11.21784090667345	23.51219255352808
H	9.09900469413102	11.94710242021567	24.22978384708433
C	16.18904457123280	4.42148814139654	19.42858147983949
C	17.65398405152447	6.23477278428507	21.07910109307076
C	10.71207344485659	11.51461297196505	22.84861595747754
C	20.07817820285270	8.49174307093109	23.82100769107527
H	20.68982075398011	8.97443849595520	24.59732170899136
C	10.73187827914721	8.70159031622662	20.26352501703093
C	18.11445073534807	7.19288419697810	26.14754608633146
H	18.15222893941639	6.86150074269227	25.09253409540181
C	11.24584716653622	10.54805941559683	21.95905976490646
C	17.41102971172560	10.73079469336165	27.44327130566204
C	16.08562081305777	4.38098971031778	20.82720289158561
H	15.43387283459085	3.62970644271050	21.28548723754626
C	11.25430766424092	12.91275951031896	22.92335886746555
C	13.85958117062836	6.58047168188140	24.66615285723726
H	13.17452679775354	5.99438433947024	24.02745605362536
H	13.69275978633138	6.26228823329255	25.71555214587233
H	14.89766357143265	6.31914956135610	24.39600058821938
C	18.40999437537430	11.83311960522665	23.89112971505150
H	17.89986891707778	11.42254396675476	22.98588994493214
C	17.64209066766832	11.49351393570193	26.28592002993558
H	17.52434773918282	12.58258741958519	26.34952357527369
C	11.96446025084976	6.80385139515544	21.47475293671235
H	12.10481095260816	7.61060377975407	22.22246655211118
C	16.81275338127550	5.24097247228626	21.67022911987247
C	10.98472783432880	5.77872051065765	22.07553458201104
H	10.79341910512905	4.94882492603707	21.36418821510005
H	10.00993774900619	6.24661840704056	22.31708270481837
H	11.39167506625301	5.34069105463113	23.00981515682845
C	14.49437099873436	9.20703580810056	25.98089864172533
H	15.56632327950917	9.34475939264772	25.75407392613255
H	14.42250147521843	8.54953221733391	26.87074377574693
H	14.07813670618904	10.18931490897140	26.26131209982308
C	10.16908408745348	9.24530025001775	19.06534504173240
C	16.94547073479546	6.44762245369038	26.81106720254835
H	15.97803063091443	6.72601435936482	26.35536656533682
H	16.88106592370566	6.65341548126874	27.89912281224421
H	17.07547068756910	5.35222304376887	26.69616966717626
C	10.77383610600441	7.18893875333383	17.85026575743825
C	20.02468759375917	6.15561296817978	18.37603425424905
H	19.62524113084780	5.51008075611944	17.56725897086208
H	20.46631093647290	5.48721991098040	19.14206146034040
H	20.84043305948611	6.77126254167439	17.94445236207373
C	14.59125171895603	12.39056827436758	24.59480145472499
H	14.99093468921004	12.32003826726895	23.56211517788233
H	15.07910778987088	11.59745484255308	25.18715311800105
H	14.91117706658713	13.36033308255306	25.02543847760601
C	19.45446755700727	6.80347351682888	26.80023602865485
H	19.60391070702354	5.70483508377438	26.76275998252845
H	19.48255614134795	7.11567278510753	27.86483523132829
H	20.31160561412042	7.28102990932357	26.28713710380466
C	10.19630708326899	8.46906626705298	17.89627903670465
H	9.73805627488232	8.86808614531824	16.97886362551696
C	16.81785517286052	4.97294690242237	23.17837802888929
H	16.66915386809730	5.94577832522692	23.69619132123693
C	10.45773971873830	9.44798141598999	21.54111450947730
C	12.79833145843677	14.60827169667491	23.74937611725203
H	13.65780454945275	14.87442720909558	24.38206663818325
C	11.38156190842467	6.70839526910144	19.01870068869819
H	11.84989041191977	5.71441052848181	19.00954216162317
C	18.98549557837578	10.40448666330789	20.44270260012306
H	18.82416606383186	9.35764237976761	20.14417807020098
H	18.97035054924100	10.44479893136547	21.54456552129301
H	19.97689068753873	10.74308989671149	20.08353617376491
C	13.34098987956244	6.18228063144158	21.21163321932133
H	13.29980087922315	5.36176571359365	20.46948748349496
H	13.76538938110822	5.75781329981733	22.13704175203108
H	14.06717251216484	6.93107550582469	20.84172110863732
C	14.69845963017980	7.92517409147320	18.22070440674764
H	14.50112822699500	7.51137647488485	17.21261637278193
H	13.84558990473212	7.66950620968237	18.87523791607023
H	15.60916594161527	7.46827693514613	18.64422842304943
C	18.32259647914406	7.98391594347343	17.91955210510973
H	19.11055523053814	8.60721042829298	17.44883265115865
H	17.57194561517995	8.65347566209678	18.38366839421525
H	17.81861842694251	7.41750450131685	17.11024612308543
C	13.15345881441668	10.17548554753216	17.58360934753361
H	13.12227479652368	11.23748587731226	17.27667294646801
H	12.42578966333105	10.01009971227319	18.39740937638991
H	12.85766478180354	9.54697528478539	16.72054224155819

C	16.96249807053074	11.39068160264701	28.73860053371892
H	16.97873517104180	10.60338862225891	29.52363112949541
C	19.93146594498166	11.84473870696802	23.63595994802852
H	20.32866172844176	10.84794843766991	23.37607297607920
H	20.46550255240282	12.19844428039952	24.54151370455975
H	20.18104956138723	12.53582116738151	22.80486499313848
C	18.19023176086013	13.09456126717861	20.39160183794517
H	19.28530776016306	13.21293348716247	20.27203774729561
H	17.92754606951405	13.17956902528483	21.45855159740410
H	17.69010565588044	13.90917405442672	19.83720022830199
C	15.72515079583885	10.02018281431506	16.58203415875460
H	15.17745799025665	9.51433295723187	15.76138806820513
H	16.75062317194045	9.61922963765947	16.61778531958453
H	15.77693436582089	11.10218743114386	16.36087869568183
C	12.47023591000255	12.37333545496617	26.02089379866118
H	11.37356942703914	12.21455481801929	26.00474479207765
H	12.65793288959312	13.38540490157396	26.43450148398383
H	12.91262396674912	11.63397171548762	26.71675235612732
C	15.19930780074889	13.72797757744691	18.18502811056375
H	14.93012775059463	13.07874601803350	17.33039105801020
H	16.29438859318965	13.87421176112405	18.17604703882836
H	14.71075102961842	14.71433045362509	18.05750670127111
C	12.84216704243692	13.11466940632858	19.59848761567295
H	12.59308148755439	14.13917116468579	19.25961310665784
H	12.37106274739065	12.96628173705609	20.58887137847294
H	12.41866942988204	12.37895250683946	18.89477690094007
C	12.12550675594333	15.62329208224184	23.05053722502767
C	15.72748419459176	3.99664735776145	23.64403273045117
H	14.71744154767334	4.27393326190198	23.29001953405426
H	15.69529129061841	3.96748056378475	24.75048283524135
H	15.94036308511067	2.96575632013442	23.29403698158191
C	15.38405058103868	3.50732428722670	18.51042615982321
H	16.12201645195857	2.99014778490804	17.85530231754643
C	9.49940306027905	10.61913363325724	19.03090981221540
H	9.98321797542216	11.23394013335082	19.81783346054699
C	17.94233196059205	13.28577637171176	24.06574284920148
H	18.21598708938070	13.89017404967445	23.17894764922221
H	18.44522287397062	13.76765012116864	24.92826132656637
H	16.85169494172492	13.36736590084022	24.21064893779082
C	10.56213393966007	13.92359738895224	22.19229305740358
C	17.91501961770898	12.51483420577877	29.18184583327638
H	17.91187588606047	13.35835346339564	28.46019485930221
H	18.95781970039837	12.14959522043369	29.26945135639481
H	17.60890223538834	12.92344219640454	30.16632939475917
C	18.18528914408920	4.41544557540679	23.63235660121016
H	18.41636106082742	3.47514667392180	23.09083757650797
H	18.16079548653372	4.18571981865883	24.71675080919522
H	19.01466004650434	5.12320915025428	23.45883403145848
C	11.01920741301932	15.25183456559050	22.27150540412886
H	10.47815688444631	16.03750801043603	21.72122822674126
C	12.53016145020604	17.08384513730314	23.19415472176516
H	11.87925610418963	17.66848034199117	22.50780370822240
C	9.68153395065247	11.35333004101039	17.69183911204304
H	9.11725088017691	10.86591575621295	16.87090663078611
H	10.74320407837122	11.40582507491089	17.38243357000464
H	9.29747696788414	12.38818131107696	17.77228443290331
C	14.46627948057995	4.33895915916514	17.59294614512141
H	15.03799844813993	5.07499304853152	16.99447877626752
H	13.72732203495801	4.90678957065649	18.19400882036355
H	13.91101039256204	3.68794816205427	16.88797184012927
C	18.23178542548536	11.63728099480135	18.02059225917436
H	18.27171327389548	10.65206795175482	17.53019724750307
H	19.24583112220976	12.08356080001401	18.00556059140063
H	17.54497078532594	12.28534719347908	17.44928615890948
C	14.97900828468198	14.22822889604085	20.99308689621370
H	16.04873795258256	14.29155117397335	21.24723521969283
H	14.41832852876077	13.96938058469152	21.91041464033162
H	14.62268923358119	15.21321986740833	20.63168370819025
C	15.51196063283069	11.89578034335628	28.61523047061159
H	15.42672794068288	12.67000775406202	27.82405165753187
H	15.16350033227026	12.34548535120981	29.56746898038218
H	14.82368269395158	11.06935654709009	28.34982625819644
C	14.57938698095981	2.42998497473602	19.24677771369612
H	14.06819898144365	1.76652538742268	18.52137777110814
H	13.79534803710787	2.87946875474883	19.89205619853022
H	15.22311159867886	1.79501612729112	19.88735966208042
C	9.33590569043627	14.26415328924966	19.97568710405635
H	10.22327778134621	13.93931876713140	19.39925382647633
H	9.34566859577555	15.37229230118285	20.01923893201216
H	8.43171674775858	13.97251481072419	19.40419091301707
C	12.25792949344029	17.57844765256072	24.62873152867576
H	12.89342659214803	17.04153080767154	25.36356607719858
H	11.20041242325489	17.41407882859929	24.91634335601062
H	12.47749684898340	18.66164027082645	24.72198766714823

```
C	13.99046079638060	17.33948236171218	22.78381812158485
H	14.16753595544162	17.06370007506422	21.72519484835443
H	14.69769127726379	16.75466526303008	23.40837724852389
H	14.25023083908379	18.41090058827309	22.90290767897311
C	7.99800209547034	10.52357107568992	19.36911859922375
H	7.53202018796877	11.53023836282009	19.36241702178089
H	7.82755724667950	10.07853872136764	20.36641374593378
H	7.47054879989915	9.89833171284472	18.61944365691477
C	9.30453545680750	13.63074455448906	21.37631951530727
H	9.23537885159718	12.53350468522210	21.24245616680598
C	8.04487261606443	14.08205162933823	22.14196383294761
H	7.12724938070111	13.82367756599351	21.57480742018495
H	8.05041840690245	15.18040944454787	22.30017694061504
H	7.97725844919314	13.60251364687976	23.13730795702191
C	11.99407978269190	6.67840382476588	15.69847706700771
H	11.93856970665254	6.15220191913956	14.72376816100064
H	12.91125150153869	6.33510926004337	16.21822045992694
H	12.10336014294414	7.76292870563573	15.49391554244013
C	10.74405119443001	6.38991001788762	16.55377800608640
H	9.86309445823986	6.75711185665225	15.98244358947203
C	10.55999271360913	4.88094220202227	16.77627687818845
H	10.42346456960975	4.35961785678434	15.80758798332202
H	9.67490869847814	4.66884168373609	17.40859547927600
H	11.44545207833132	4.42818761326202	17.26693917999674
```

Coordinates of compounds for mechanistic study

* xyz [(PhSn)$_2$Ir(PMe$_3$)$_2$] PMe$_3$, Me$_3$SiN$_3$

```
C	-2.014729000	-0.857028000	-4.076398000
C	2.621659000	-1.052063000	-4.376288000
C	1.829096000	-1.025942000	-3.233026000
C	0.137933000	-5.405497000	-4.334754000
C	-0.047094000	-4.423280000	-3.366299000
C	2.878775000	0.130211000	-5.069166000
C	-3.626092000	-3.002180000	-3.045514000
C	0.095590000	-6.751308000	-3.977099000
C	-4.459877000	-0.269155000	-2.771618000
C	1.289760000	0.178284000	-2.764460000
C	-0.272672000	-4.776813000	-2.029317000
C	2.357363000	1.335959000	-4.608840000
C	-0.121850000	-7.115271000	-2.651565000
C	1.576961000	1.362519000	-3.455678000
C	-0.297117000	-6.133188000	-1.680272000
C	-5.236843000	-2.136312000	0.760515000
C	-4.599342000	0.674096000	0.942409000
C	-3.318921000	-1.215736000	2.681550000
P	-2.976206000	-1.344583000	-2.585283000
P	-3.809544000	-0.986913000	0.921861000
Sn	0.002036000	0.202390000	-1.015808000
Sn	-0.604049000	-3.280261000	-0.490545000
Ir	-2.024956000	-1.244284000	-0.571704000
H	-2.604525000	-1.032392000	-4.983451000
H	3.041046000	-1.993133000	-4.724745000
```

H	-1.081013000	-1.421307000	-4.133981000
H	-1.757574000	0.205604000	-4.019867000
H	0.315481000	-5.121966000	-5.368427000
H	-4.175520000	-2.946779000	-3.992451000
H	-2.799423000	-3.711460000	-3.151754000
H	-4.854527000	-0.338741000	-3.791753000
H	3.493519000	0.109281000	-5.964226000
H	0.241289000	-7.519070000	-4.731428000
H	-4.191478000	0.770887000	-2.564783000
H	-4.295045000	-3.367203000	-2.260796000
H	-5.242224000	-0.574926000	-2.071861000
H	2.565150000	2.257428000	-5.145085000
H	-0.148573000	-8.164521000	-2.372008000
H	-5.707012000	-2.027021000	-0.220891000
H	-5.050556000	0.894215000	-0.028627000
H	-4.886495000	-3.167872000	0.860517000
H	-3.839128000	1.433376000	1.148617000
H	-5.985037000	-1.933813000	1.534971000
H	-2.932746000	-2.229441000	2.822498000
H	-2.523408000	-0.509765000	2.937366000
H	-5.373909000	0.721825000	1.716175000
H	-4.173068000	-1.054493000	3.349176000
H	-0.010203000	-3.377890000	-3.664199000
H	-0.453273000	-6.435621000	-0.646319000
H	1.644077000	-1.958350000	-2.699982000
H	1.194218000	2.318574000	-3.101213000
N	6.786115000	-0.416296000	8.311560000
N	6.742182000	-1.524441000	8.562804000
N	6.599739000	-2.709793000	8.792872000
Si	7.844594000	-3.732963000	9.545402000
C	7.058447000	-5.421846000	9.615164000
H	6.811447000	-5.779878000	8.610351000
H	7.733243000	-6.149704000	10.078917000
H	6.133982000	-5.396865000	10.201612000
C	9.372390000	-3.704077000	8.463717000
H	9.158573000	-4.101328000	7.465957000
H	9.755198000	-2.683495000	8.346546000
H	10.174767000	-4.307328000	8.904241000
C	8.223567000	-3.056942000	11.249631000
H	8.607728000	-2.031940000	11.194464000

H	7.327352000	-3.047813000	11.878865000
H	8.983255000	-3.667926000	11.751059000
P	-3.579822000	11.158080000	-3.102594000
C	-4.214198000	12.702589000	-2.290227000
H	-5.230583000	12.535354000	-1.920040000
H	-3.583115000	12.952365000	-1.431376000
H	-4.223746000	13.554070000	-2.981734000
C	-4.646652000	11.140792000	-4.622707000
H	-4.621591000	12.102884000	-5.149059000
H	-4.305059000	10.355404000	-5.304358000
H	-5.681145000	10.915708000	-4.344790000
C	-2.002462000	11.812970000	-3.832170000
H	-1.289562000	12.032062000	-3.030947000
H	-1.552484000	11.055166000	-4.481265000
H	-2.172084000	12.726117000	-4.416070000

*

Product of 3+3 Cycloaddition

* xyz 1 1

C	2.441463000	3.653782000	-0.262872000
C	3.779869000	2.278605000	-4.455208000
C	2.827756000	1.884350000	-3.519852000
C	-2.880428000	-3.689765000	-3.275005000
C	-1.926457000	-3.009451000	-2.522827000
C	5.041024000	1.688258000	-4.454655000
C	-0.196461000	4.355659000	-1.069121000
C	-4.235497000	-3.502038000	-3.018499000
C	0.599749000	4.530768000	1.677026000
C	3.131651000	0.891440000	-2.582404000
C	-2.324016000	-2.127944000	-1.511630000
C	5.345213000	0.695750000	-3.527162000
C	-4.638074000	-2.625949000	-2.014329000
C	4.394364000	0.291111000	-2.593520000
C	-3.686931000	-1.937505000	-1.266185000
C	-3.360704000	1.891905000	-0.594624000
C	-2.781353000	3.310920000	1.796335000
C	-3.260275000	0.539152000	1.930373000
P	0.687813000	3.389755000	0.227797000
P	-2.311006000	1.756551000	0.916666000
Sn	1.662474000	0.234922000	-1.177251000
Sn	-0.771462000	-1.125854000	-0.419227000

Ir	-0.072380000	1.217319000	0.490081000
H	2.681409000	4.722565000	-0.238399000
H	3.535588000	3.042540000	-5.187865000
H	2.620438000	3.273890000	-1.272221000
H	3.111464000	3.120556000	0.417471000
H	-2.562954000	-4.369250000	-4.060862000
H	0.253028000	5.347549000	-1.190905000
H	-0.145249000	3.814524000	-2.018270000
H	0.855998000	5.549880000	1.367743000
H	5.785192000	1.998035000	-5.182340000
H	-4.978473000	-4.038348000	-3.601158000
H	1.317994000	4.215354000	2.439589000
H	-1.250544000	4.476749000	-0.804276000
H	-0.396105000	4.536135000	2.122358000
H	6.326291000	0.229472000	-3.531563000
H	-5.695009000	-2.479825000	-1.810729000
H	-2.986235000	2.702651000	-1.226508000
H	-2.463809000	4.191435000	1.231397000
H	-3.299828000	0.967608000	-1.176784000
H	-2.319320000	3.341839000	2.787501000
H	-4.407034000	2.091501000	-0.335955000
H	-3.175923000	-0.461876000	1.497828000
H	-2.847785000	0.495709000	2.942777000
H	-3.869192000	3.356298000	1.916646000
H	-4.317905000	0.818603000	1.993440000
H	-0.870327000	-3.172069000	-2.726041000
H	-4.026223000	-1.264922000	-0.482305000
H	1.841427000	2.345294000	-3.540239000
H	4.635268000	-0.496625000	-1.884779000
N	2.500342000	-1.670904000	-0.670265000
N	1.897855000	-2.630848000	-0.207899000
N	0.578704000	-2.808559000	-0.189640000
Si	0.097169000	-4.396616000	0.532144000
C	1.247341000	-4.790573000	1.953127000
H	1.193257000	-4.032251000	2.741879000
H	0.971764000	-5.753209000	2.399608000
H	2.286442000	-4.857193000	1.617740000
C	-1.657715000	-4.197542000	1.168054000
H	-1.762733000	-3.341106000	1.847001000
H	-2.395658000	-4.097482000	0.367043000

H	-1.919134000	-5.091501000	1.747533000
C	0.189093000	-5.711446000	-0.791472000
H	-0.535445000	-5.528151000	-1.591688000
H	1.189773000	-5.745676000	-1.236010000
H	-0.022584000	-6.700413000	-0.368917000
P	0.825733000	0.827505000	2.552454000
C	2.590472000	1.288553000	2.812303000
H	3.212984000	0.743890000	2.096180000
H	2.740703000	2.360177000	2.656764000
H	2.906839000	1.036707000	3.831072000
C	0.853659000	-0.938693000	3.067874000
H	1.313602000	-1.047247000	4.057393000
H	-0.167581000	-1.329858000	3.095857000
H	1.425907000	-1.516834000	2.336252000
C	0.030312000	1.623420000	4.013621000
H	0.040682000	2.711562000	3.908281000
H	-1.009052000	1.294534000	4.099357000
H	0.558785000	1.352761000	4.934871000

*

Stannaimine and N2

* xyz 1 1

C	-1.065565000	2.644354000	-2.588521000
C	6.071252000	-0.302700000	0.974016000
C	4.795722000	0.193983000	0.717416000
C	-4.605575000	-2.795731000	-2.192477000
C	-3.701083000	-1.807964000	-1.811785000
C	6.254278000	-1.661364000	1.215424000
C	-1.757769000	4.518534000	-0.575620000
C	-4.323484000	-4.134302000	-1.934510000
C	0.972696000	4.097097000	-1.266849000
C	3.698464000	-0.671024000	0.688095000
C	-2.502743000	-2.158413000	-1.182610000
C	5.163715000	-2.527224000	1.196109000
C	-3.134705000	-4.489290000	-1.301551000
C	3.889493000	-2.035692000	0.928462000
C	-2.222503000	-3.505809000	-0.931288000
C	-3.912986000	1.160150000	0.673294000
C	-3.005961000	2.862207000	2.735987000
C	-2.724467000	0.041304000	2.992073000
P	-0.562385000	3.143595000	-0.882543000

P	-2.443414000	1.393051000	1.767350000
Sn	1.704561000	0.001899000	0.252275000
Sn	-1.043364000	-0.691832000	-0.616841000
Ir	-0.390623000	1.448467000	0.698442000
H	-1.091867000	3.504695000	-3.267619000
H	6.923296000	0.371038000	0.982370000
H	-2.057407000	2.182593000	-2.556626000
H	-0.354254000	1.905836000	-2.971160000
H	-5.529193000	-2.520771000	-2.693904000
H	-1.740336000	5.246110000	-1.395128000
H	-2.771534000	4.115236000	-0.488548000
H	0.778113000	4.884121000	-2.004611000
H	7.249413000	-2.046909000	1.416931000
H	-5.030671000	-4.903460000	-2.230234000
H	1.726695000	3.410223000	-1.663132000
H	-1.512618000	5.034024000	0.357238000
H	1.377245000	4.557582000	-0.360984000
H	5.305831000	-3.587482000	1.385034000
H	-2.914947000	-5.533931000	-1.101227000
H	-3.900221000	1.893142000	-0.138804000
H	-3.014892000	3.753058000	2.101582000
H	-3.893318000	0.157170000	0.235458000
H	-2.349771000	3.046507000	3.590228000
H	-4.843483000	1.268706000	1.241559000
H	-2.668004000	-0.922513000	2.475973000
H	-1.941013000	0.055271000	3.754443000
H	-4.018786000	2.690130000	3.115558000
H	-3.705010000	0.135567000	3.473397000
H	-3.934549000	-0.767043000	-2.028052000
H	-1.288866000	-3.793839000	-0.452805000
H	4.672415000	1.258029000	0.525627000
H	3.045962000	-2.722424000	0.901902000
N	-2.538723000	-9.458016000	-2.742667000
N	-2.884962000	-8.956248000	-1.833998000
N	0.838385000	-1.461816000	-0.961214000
Si	1.475896000	-2.460991000	-2.248367000
C	3.112586000	-1.735917000	-2.826236000
H	3.866363000	-1.740781000	-2.030977000
H	3.515980000	-2.316770000	-3.663881000
H	2.990592000	-0.702197000	-3.172811000

C	1.756734000	-4.236220000	-1.694691000
H	2.486856000	-4.299478000	-0.880819000
H	0.829661000	-4.709331000	-1.353136000
H	2.142612000	-4.836728000	-2.527027000
C	0.255491000	-2.459263000	-3.681406000
H	-0.710629000	-2.894267000	-3.402097000
H	0.075944000	-1.442152000	-4.052141000
H	0.648411000	-3.046643000	-4.519497000
P	0.671415000	2.584183000	2.407858000
C	2.508021000	2.724412000	2.301080000
H	2.969676000	1.739986000	2.428821000
H	2.795027000	3.122069000	1.322888000
H	2.887029000	3.390211000	3.084387000
C	0.495980000	1.915434000	4.117751000
H	1.090701000	2.501199000	4.827674000
H	-0.548781000	1.934077000	4.438667000
H	0.837008000	0.875952000	4.128030000
C	0.249776000	4.365895000	2.658906000
H	0.575850000	4.944978000	1.789966000
H	-0.825390000	4.507817000	2.780371000
H	0.760705000	4.756643000	3.545754000

*

References

[1] C. P. Sindlinger, A. Stasch, H. F. Bettinger, L. Wesemann, "A nitrogen-base catalyzed generation of organotin(II) hydride from an organotin trihydride under reductive dihydrogen elimination," *Chemical Science* **6**, (2015): 4737–4751.
[2] L. G. Holzapfel, J. Manegold, S. F. Clewing, H. Schubert, K. Eichele, L. Wesemann, "Metathetical Exchange, Synthesis, and Carbon Dioxide Addition of Higher Homologues of Group 9 Metal Carbynes," *Angewandte Chemie International Edition* **64**, (2025): e202503930.
[3] S. R. Bahr, P. Boudjouk, "Trityl Tetrakis(3,5-Bis(Trifluoromethyl)Phenyl)Borate - a New Hydride Abstraction Reagent," *J. Org. Chem.* **57**, (1992): 5545–5547.
[4] I. Krossing, H. Brands, R. Feuerhake, S. Koenig, "New reagents to introduce weakly coordinating anions of type $Al(OR_F)_4^-$: synthesis, structure and characterization of Cs and trityl salts," *Journal of Fluorine Chemistry* **112**, (2001): 83–90.
[5] R. K. Harris, E. D. Becker, S. M. C. d. Menezes, R. Goodfellow, P. Granger, "NMR nomenclature. Nuclear spin properties and conventions for chemical shifts(IUPAC Recommendations 2001)," *Pure and Applied Chemistry* **73**, (2001): 1795–1818.
[6] G. Sheldrick, "Crystal structure refinement with SHELXL," *Acta Crystallographica Section C* **71**, (2015): 3–8.
[7] L. J. Farrugia, "ORTEP-3 for Windows - a version of ORTEP-III with a Graphical User Interface (GUI)," *Journal of Applied Crystallography* **30**, (1997): 565.
[8] L. J. Farrugia, "WinGX suite for small-molecule single-crystal crystallography," *Journal of Applied Crystallography* **32**, (1999): 837–838.
[9] L. J. Farrugia, "WinGX and ORTEP for Windows: an update," *Journal of Applied Crystallography* **45**, (2012): 849–854.
[10] C. B. Hübschle, G. M. Sheldrick, B. Dittrich, "*ShelXle* : a Qt graphical user interface for *SHELXL*," *Journal of Applied Crystallography* **44**, (2011): 1281–1284.
[11] G. M. Sheldrick, SHELXL-97, Program of Crystal Structure Refinement, University of Göttingen, Göttingen, (Germany), 1997.
[12] G. M. Sheldrick, "A short history of SHELX," *Acta Crystallographica Section A* **64**, (2008): 112–122.
[13] G. M. Sheldrick, Program for Empirical Absorption Correction of Area Detector Data, University Göttingen, Göttingen (Germany), 1996.
[14] G. M. Sheldrick, SHELXS-96, Program for the Solution of Crystal Structures, University of Göttingen, Göttingen, (Germany), 1996.
[15] G. Sheldrick, "SHELXT - Integrated space-group and crystal-structure determination," *Acta Crystallographica Section A* **71**, (2015): 3–8.
[16] D. Kratzert, I. Krossing, "Recent improvements in DSR," *Journal of Applied Crystallography* **51**, (2018): 928–934.
[17] F. Neese, "Software update: the ORCA program system, version 4.0," *Wiley Interdiscip. Rev. Comput. Mol. Sci.* **8**, (2018): e1327.
[18] F. Neese, "The ORCA program system," *Wiley Interdisciplinary Reviews: Computational Molecular Science* **2**, (2012): 73–78.
[19] F. Neese, "Software update: The ORCA program system—Version 5.0," *WIREs Computational Molecular Science* **12**, (2022): e1606.
[20] S. Grimme, S. Ehrlich, L. Goerigk, "Effect of the damping function in dispersion corrected density functional theory," *Journal of Computational Chemistry* **32**, (2011): 1456–1465.

[21] S. Grimme, J. Antony, S. Ehrlich, H. Krieg, "A consistent and accurate ab initio parametrization of density functional dispersion correction (DFT-D) for the 94 elements H-Pu," *The Journal of Chemical Physics* **132**, (2010): 154104–154119.
[22] F. Weigend, "Accurate Coulomb-fitting basis sets for H to Rn," *Physical Chemistry Chemical Physics* **8**, (2006): 1057–1065.
[23] F. Weigend, R. Ahlrichs, "Balanced basis sets of split valence, triple zeta valence and quadruple zeta valence quality for H to Rn: Design and assessment of accuracy," *Physical Chemistry Chemical Physics* **7**, (2005): 3297–3305.
[24] B. Metz, H. Stoll, M. Dolg, "Small-core multiconfiguration-Dirac–Hartree–Fock-adjusted pseudopotentials for post-d main group elements: Application to PbH and PbO," *The Journal of Chemical Physics* **113**, (2000): 2563–2569.
[25] D. Andrae, U. Häussermann, M. Dolg, H. Stoll, H. Preuß, "Energy-adjusted ab initio pseudopotentials for the second and third row transition elements," *Theoretica chimica acta* **77**, (1990): 123–141.
[26] A. D. Becke, "Density-functional exchange-energy approximation with correct asymptotic behavior," *Physical Review A* **38**, (1988): 3098–3100.
[27] J. P. Perdew, W. Yue, "Accurate and simple density functional for the electronic exchange energy: Generalized gradient approximation," *Physical Review B* **33**, (1986): 8800–8802.
[28] U. Ekström, L. Visscher, R. Bast, A. J. Thorvaldsen, K. Ruud, "Arbitrary-Order Density Functional Response Theory from Automatic Differentiation," *Journal of Chemical Theory and Computation* **6**, (2010): 1971–1980.
[29] G. A. Zhurko, Chemcraft - graphical software for visualization of quantum chemistry computations. Version 1.8, build 682. https://www.chemcraftprog.com.
[30] E. D. Glendening, J. K. Badenhoop, A. E. Reed, J. E. Carpenter, J. A. Bohmann, C. M. Morales, P. Karafiloglou, C. R. Landis, F. Weinhold, NBO 7.0*,* Theoretical Chemical Institute, University of Wisconsin, Madison, 2018.
[31] G. Knizia, "Intrinsic Atomic Orbitals: An Unbiased Bridge between Quantum Theory and Chemical Concepts," *Journal of Chemical Theory and Computation* **9**, (2013): 4834–4843.
[32] M. P. Mitoraj, A. Michalak, T. Ziegler, "A Combined Charge and Energy Decomposition Scheme for Bond Analysis," *Journal of Chemical Theory and Computation* **5**, (2009): 962–975.
[33] E. Van Lenthe, E. J. Baerends, "Optimized Slater-type basis sets for the elements 1–118," *Journal of Computational Chemistry* **24**, (2003): 1142–1156.
[34] G. te Velde, F. M. Bickelhaupt, E. J. Baerends, C. Fonseca Guerra, S. J. A. van Gisbergen, J. G. Snijders, T. Ziegler, "Chemistry with ADF," *Journal of Computational Chemistry* **22**, (2001): 931–967.
[35] SCM, ADF2022*,* Theoretical Chemistry, Vrije Universiteit, Amsterdam, The Netherlands, 2022.
[36] "ADF2019.3,," *www scm.com, ADF2019.3, Vrije Universiteit Amsterdam*, (2019).
[37] S. K. Wolff, T. Ziegler, "Calculation of DFT-GIAO NMR shifts with the inclusion of spin-orbit coupling," *The Journal of Chemical Physics* **109**, (1998): 895–905.
[38] G. Schreckenbach, T. Ziegler, "Calculation of NMR Shielding Tensors Using Gauge-Including Atomic Orbitals and Modern Density Functional Theory," *The Journal of Physical Chemistry* **99**, (1995): 606–611.

[39] J. Autschbach, E. Zurek, "Relativistic Density-Functional Computations of the Chemical Shift of ^{129}Xe in Xe@C60," *The Journal of Physical Chemistry A* **107**, (2003): 4967–4972.
[40] M. Krykunov, T. Ziegler, E. v. Lenthe, "Hybrid density functional calculations of nuclear magnetic shieldings using Slater-type orbitals and the zeroth-order regular approximation," *Int. J. Quant. Chem.* **109**, (2009): 1676–1683.
[41] S. K. Wolff, T. Ziegler, E. van Lenthe, E. J. Baerends, "Density functional calculations of nuclear magnetic shieldings using the zeroth-order regular approximation (ZORA) for relativistic effects: ZORA nuclear magnetic resonance," *The Journal of Chemical Physics* **110**, (1999): 7689–7698.
[42] J. Autschbach, "The role of the exchange-correlation response kernel and scaling corrections in relativistic density functional nuclear magnetic shielding calculations with the zeroth-order regular approximation," *Molecular Physics* **111**, (2013): 2544–2554.
[43] V. Ásgeirsson, B. O. Birgisson, R. Bjornsson, U. Becker, F. Neese, C. Riplinger, H. Jónsson, "Nudged Elastic Band Method for Molecular Reactions Using Energy-Weighted Springs Combined with Eigenvector Following," *Journal of Chemical Theory and Computation* **17**, (2021): 4929–4945.
[44] S. Grimme, A. Hansen, S. Ehlert, J.-M. Mewes, "r2SCAN-3c: A “Swiss army knife” composite electronic-structure method," *The Journal of Chemical Physics* **154**, (2021): 064103.
[45] F. Neese, F. Wennmohs, U. Becker, C. Riplinger, "The ORCA quantum chemistry program package," *The Journal of Chemical Physics* **152**, (2020): 224108.
[46] E. Caldeweyher, C. Bannwarth, S. Grimme, "Extension of the D3 dispersion coefficient model," *The Journal of Chemical Physics* **147**, (2017): 034112.
[47] E. Caldeweyher, S. Ehlert, A. Hansen, H. Neugebauer, S. Spicher, C. Bannwarth, S. Grimme, "A generally applicable atomic-charge dependent London dispersion correction," *The Journal of Chemical Physics* **150**, (2019): 154122.
[48] H. Kruse, S. Grimme, "A geometrical correction for the inter- and intra-molecular basis set superposition error in Hartree-Fock and density functional theory calculations for large systems," *The Journal of Chemical Physics* **136**, (2012): 154101.
